# Estimating Growth in Height from Limited Longitudinal Growth Data Using Full-Curves Training Dataset: A Comparison of Two Procedures of Curve Optimization—Functional Principal Component Analysis and SITAR

**DOI:** 10.3390/children8100934

**Published:** 2021-10-18

**Authors:** Miroslav Králík, Ondřej Klíma, Martin Čuta, Robert M. Malina, Sławomir Kozieł, Lenka Polcerová, Anna Škultétyová, Michal Španěl, Lubomír Kukla, Pavel Zemčík

**Affiliations:** 1 Department of Anthropology, Faculty of Science, Masaryk University, 611 37 Brno, Czech Republic; cuta@sci.muni.cz (M.Č.); polcerova@muni.cz (L.P.); skultetyova@mail.muni.cz (A.Š.); 2 IT4Innovations Centre of Excellence, Brno University of Technology, 612 00 Brno, Czech Republic; iklima@fit.vutbr.cz (O.K.); spanel@fit.vut.cz (M.Š.); zemcik@fit.vut.cz (P.Z.); 3 Department of Kinesiology and Health Education, The University of Texas at Austin, Austin, TX 78712-1415, USA; rmalina@1skyconnect.net; 4 School of Public Health and Information Sciences, University of Louisville, Louisville, KY 40202, USA; 5 Department of Anthropology, Hirszfeld Institute of Immunology and Experimental Therapy, Polish Academy of Sciences, 53-114 Wrocław, Poland; slawomir.koziel@hirszfeld.pl; 6 Outpatient Primary Care Pediatric Center, 625 00 Brno, Czech Republic; lubomir.kukla@recetox.muni.cz

**Keywords:** human growth, growth modelling, functional data analysis, Sitar

## Abstract

A variety of models are available for the estimation of parameters of the human growth curve. Several have been widely and successfully used with longitudinal data that are reasonably complete. On the other hand, the modeling of data for a limited number of observation points is problematic and requires the interpolation of the interval between points and often an extrapolation of the growth trajectory beyond the range of empirical limits (prediction). This study tested a new approach for fitting a relatively limited number of longitudinal data using the normal variation of human empirical growth curves. First, functional principal components analysis was done for curve phase and amplitude using complete and dense data sets for a reference sample (Brno Growth Study). Subsequently, artificial curves were generated with a combination of 12 of the principal components and applied for fitting to the newly analyzed data with the Levenberg–Marquardt optimization algorithm. The approach was tested on seven 5-points/year longitudinal data samples of adolescents extracted from the reference sample. The samples differed in their distance from the mean age at peak velocity for the sample and were tested by a permutation leave-one-out approach. The results indicated the potential of this method for growth modeling as a user-friendly application for practical applications in pediatrics, auxology and youth sport.

## 1. Introduction

During the past century or so, knowledge about human growth has led to the development of various approaches to the modeling of growth data. The human growth curve spans birth to adulthood, but its description and modeling cannot be adequately performed by simple mathematical means, e.g., one single logistic curve or a single Gompertz curve which are sufficient for some growth processes.

A longitudinal growth study optimally represents a cohort of children which is measured repeatedly annually or semi-annually across a long time interval, ideally from birth to adulthood. For subjects who are represented in the sample for the entire interval from birth/infancy to 18 years, the growth data are relatively dense and complete. For modeling of such data, i.e., fitting a growth curve and extracting/estimating growth parameters, a variety of models have been developed for this task. The methodological principles range from polynomials/complex parametric models [1,2,3] through composite models of several additive curves [4,5,6,7,8], population average B-spline fitting [9,10,11], and functional data analysis [12,13,14]. Although the methods differ in many respects, including mathematical background and biological plausibility of the extracted coefficients/parameters, many work very well and have been used successfully for the description of human growth trajectories and the study of various factors affecting growth, providing the data are complete and without gross measurement errors.

In contrast, difficulties arise when the growth data are relatively sparse and/or limited to a relatively low number of observations with significant gaps between measurements. This is often noted in short time-span pediatric records and in short longitudinal studies of youth athletes [15,16]. For practical or financial reasons, and age at entry into specialized sport programs, observations are often limited, e.g., 4 to 7 for each subject. In these instances, the applicability of the above-mentioned growth models is limited. In this situation, the task of modelling is not only *descriptive*, but also includes *interpolation*—fitting a reliable curve into the limited data points, estimating the growth trajectory in between them, and estimating the growth parameters from the resulting curve. Growth modeling with a limited number of observations is also difficult due to measurement variability (noise). The SITAR protocol (a method proposed and used by Cole et al., based on superimposition by translation and rotation of analyzed curves) [11] represents a remarkable advantage and allows relatively reliable estimates of growth curve parameters with a small and variable number of measurement points. The method creates a model curve by superimposing and averaging splines fitted to the individual data (i.e., from the data itself) and the average model spline is then used to fit the original individual data.

An additional problem involves *calendar* age *per se*. This is apparent in short term studies that begin relatively late, for example, at 11–12 years and conclude relatively early, 15-16 years. The interval includes considerable variation in growth velocity and maturity timing among subjects so that there is a high probability that an important growth milestone of adolescent growth will not be captured in the empirical data of some or many subjects [see 16]. In addition to description and interpolation, such short-term studies require *extrapolation*—a lengthening of the model (curve) to the past (in early maturing subjects) or to the future (in late maturing subjects) beyond the empirical range of the data. Depending on modeling methods, this can be done from the data *per se* (which allows only simple mathematical models to be considered due to the limited number of empirical values), or by a type of external “training” of the model by information on an appropriate growth curve, e.g., in the form of an equation statistically extracted by an abstraction from a sample of complete growth curves, by a mathematical model accommodated to them (e.g., Preece–Baines model), or by a sample-based curve extracted from registered and averaged incomplete individual curves and uniformly warped back to model individual trajectories as in SITAR. Nevertheless, even the SITAR method may have difficulties with short term and incomplete data. Although the SITAR procedure allows extrapolation beyond the limits of the range of empirical data (i.e., it covers the age range as wide as the sum of all data but wider than each individual record), it is questionable for application at the individual level.

Even if an indicator of the current biological age of a prepubertal child is available, any prediction of the future trajectory of growth and its final status is difficult. For example, predicted maturity offset, defined as the time before/after peak height velocity (PHV) (in pubertal phase), and predicted age at PHV, estimated as calendar age (CA) minus predicted offset [17,18], have been proposed as an indicator of maturity. Unfortunately, the precision of the predictions is not optimal or satisfactory [19].

The present study describes an approach for fitting human growth curves for height to a relatively limited number of longitudinal observations and compares the fitted curves to estimates based on the SITAR method. The approach is based on functional data analysis (FDA) and functional principal component analysis (FPCA) [12]. The study drew from artificial samples using incomplete empirical height records during the adolescent spurt that require extrapolation for estimates of age at PHV. The focus is on longitudinal data (time series) of repeated measurements at the individual level and does not address the cross-sectional research design.

## 2. Materials and Methods

### 2.1. General Description of the Approach

Traditional models rarely represent empirical growth curves for height that include all aspects of variation in growth; rather, they provide mathematically defined ideal curves, i.e., a simplification or an approximation. The mathematical simplicity of the model may or may not reflect the biologically plausible process at the expense of the empirical data which may be influenced by noise and random error. In situations with a small number of measurement points within a limited interval of postnatal growth, such an approach may be sufficient by statistical criteria, i.e., the simple/ideal curve fits a small number of points well. However, it does not necessarily mean that the model is the best possible from a biological perspective which may become evident when compared with a model of the same growth trajectory based on a full dataset. The SITAR approach provided a breakthrough [11], but the model operates with uniform deformation of a single curve which may be different in details for each of the individual growth curves from which it is derived (represented by B-splines).

Using an extreme example, if two measurement points only are available, the most parsimonious model would be a straight line through the two points. The line, however, is not the best model of the true growth apparent in the two points. Growth is more complex and use of the best fitting model has limitations. Rather, the model should fit the data points well, but at the same time should be realistic from an empirical point of view, i.e., look like the growth curve for height. The issue of concern is the weight which should be applied for each of the two criteria.

**Figure 1:**
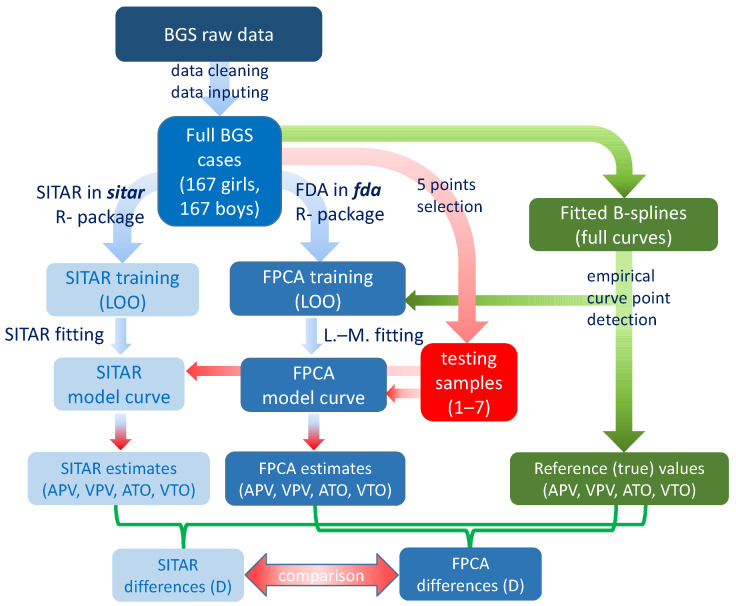
Scheme of the procedure of this research; BGS—Brno Growth Study, SITAR—a method based on superimposition by translation and rotation, FDA—Functional Data Analysis, FPCA—Functional Principal Component Analysis (FPCA), LOO—leave-one-out permutation procedure, L.-M.—Levenberg–Marquardt optimization algorithm, APV—age at peak velocity in puberty, VPV—peak velocity in puberty, ATO—age at take-off, VTO—velocity at take-off, D—difference between an estimate and respective reference.

Contrary to traditional models, the proposed approach in the present study is based on the tracing of empirical growth curves for individuals (Figure 1). Empirical variation in individual growth trajectories in a large data set (reference sample) based on complete longitudinal data is initially considered. The data were modeled by fitting complete growth curves ranging from birth to 18 years. The empirical curves serve as models to fit to incomplete data. Nevertheless, even with a large reference sample, the pool of empirical curves does not cover all possible growth trajectories. Given the limited variation of available curves and using a limited number of empirical curves, modeling new data may not always result in an optimal fit. Therefore, variation among empirical curves based on the functional data analysis (FDA) model in combination with principal component analyses (PCA) of artificial growth curves reflecting empirical variation was generated. Using an iterative procedure, the generated empirical curves were used as growth models and those which were best fitted were selected by means of advanced optimization criteria.

### 2.2. Reference Sample—The Brno Growth Study

An archived database collected within the frame of the Brno Growth Study (BGS) was used as a reference sample applied for the testing [20]. This cohort study began in 1961 under the leadership of Bouchalová at the Department of Social Medicine, Faculty of Medicine, University of Jan Evangelista Purkyně and the Pediatric Research Institute in Brno, Czech Republic. A three-birth-years cohort was recruited during the period between January 1961 and June 1964 [20,21,22,23] and continued until 1980. A total of 555 participants were measured longitudinally (278 boys and 277 girls), and 334 remained until the end of the study (up to 18 years of age). Body dimensions were measured at birth and subsequently every three months during the first year and every six months in subsequent years with few exceptions within a window of ±14 days. Detailed raw data plots are available in Supplementary Materials (Figure S1–S2).

The present study is limited to the 334 individuals with complete or almost complete growth curves. The remaining participants (*N* = 221) were followed across variable intervals, and many dropped out before the interval of puberty and adolescence (*n* = 112). Drop-out was largely explained by the parents as due to loss of interest (if an explanation was provided) and at times due to the family moving from the Brno region. Differences in body height between those who persisted in the study and those who dropped out were negligible and not statistically significant in girls or boys at birth and one year of age (Supplementary Materials, Table S1).

### 2.3. New Computational Approach

The raw data of body height from paper forms of the Brno Growth Study transcribed into MS Excel were initially scanned numerically and visually for outliers and errors in transcription of the raw data. After cleaning and correction, if necessary, all cases were largely complete, i.e., included 39 measurements spanning birth to 18 years. Occasionally, missing values were interpolated using the *na_interpolation* function available in the R-package *imputeTS* [24] and the Stineman interpolation from the *stinepack* package [25,26]. Complete raw data in individual plots, including visualization of subsequent processing and final estimates of growth milestones at the individual level are available in Supplementary Materials (Figure S3).

**Figure 2:**
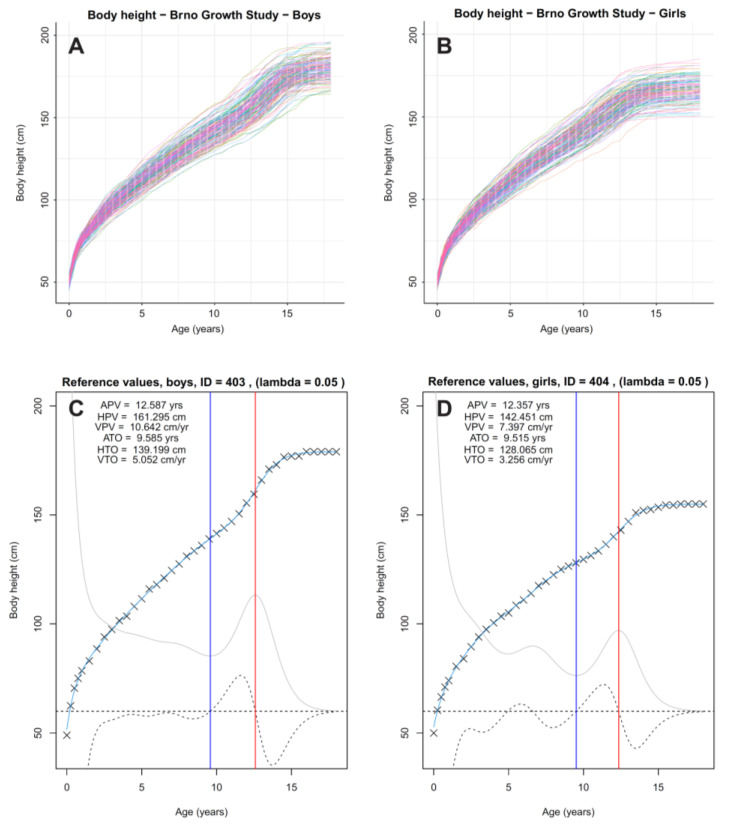
Individual longitudinal data (points are connected by segments creating an impression of curves) for height of individual subjects from the Brno Growth Study for boys (**A**) and girls (**B**); two examples (**C**) a boy, (**D**) a girl of estimated growth milestones used as reference values: crosses—raw measurement, light blue solid line—distance curve (fitted B-spline), solid grey line—velocity curve (1st derivative of the distance curve), dashed grey curve—acceleration curve (2nd derivative of the distance curve), dashed horizontal line—zero value for velocity and acceleration curves (at value 60 of the *y*-axis), blue vertical—Age at Take-off (ATO), red vertical—Age at Peak Velocity (APV); to both velocity and acceleration values a constant (+60) was added and simultaneously they were multiplied by another constant (+5) to be visible in one data-dense plot and readable against the same *y*-axis of the distance curve.

The computational approach used for curve fitting with FDA is described in Ramsay and Silverman [12,14]. The computational routines and scripts that were applied, including the R-package *fda*, are available in Ramsay et al. [13,27]. B-spline curves were fitted to the raw data for each individual raw data (with some extrapolated missing values) using the *smooth monotone* function with the setting for number of basic points on 43, based on the number of measurement points (39) plus the order of the spline (6) minus 2, and lambda = 0.05 (smoothing parameter; the value was set empirically to be optimal for the given data type). The 334 individual spline curves were used to develop individual growth milestones during puberty and adolescence by computing the velocity curve (mathematically: first derivative of the growth curve spline) and the acceleration curve (mathematically: second derivative of the growth curve spline), and subsequently detecting the local maximum and/or minimum on the curves. The following were detected: age at take-off (ATO), age at peak velocity (APV) and respective velocities and heights at take-off and peak velocity (see Figure 2, those for all 334 subjects are presented in Supplementary Materials, Figure S3).

**Figure 3:**
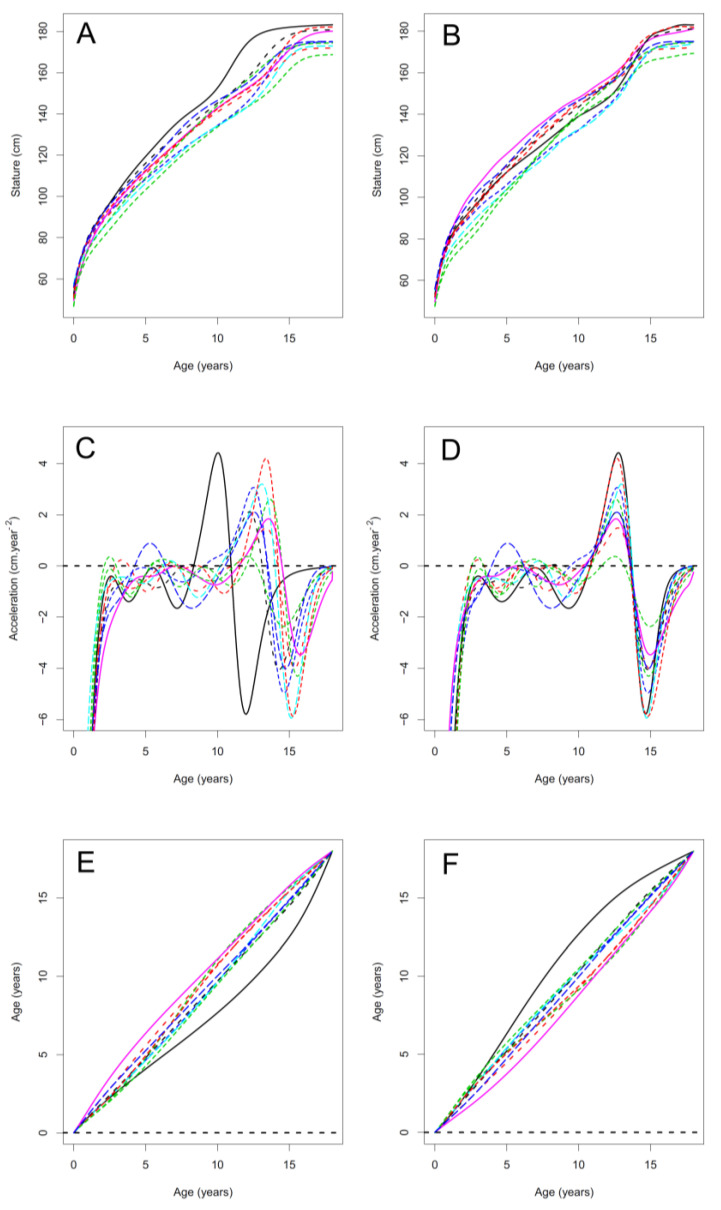
Analytical procedure of the first Functional Data Analysis in an example of te n individual trajectories: (**A**) original distance curves (B-splines), (**B**) individual distance curves after registration (warping) to identical phase (warped to identical APV, i.e., mean population APV), (**C**) individual acceleration curves corresponding to the distance curves in the plot A, (**D**) identical acceleration curves after registration of the phase corresponding to the distance curves in the plot B, (**E**) time-warping functions, (**F**) inverse time-warping functions. The course of the analysis can be described: from (**A**), (**C**) is determined, this is registered to (**D**), then E is extracted and applied to A to be warped to B. (**F**) (inverse time-warping function) is used in the reverse process for generating artificial curves applied in the fitting and growth estimation of new data.

At the same time, these curves with B-spline bases were used as the input for creating the Functional Data Analysis (FDA) model [12]. In the recently applied version of the procedure, a crucial aspect of the model creation is the separation of growth curve *phase* and growth curve *amplitude* [13]. The key step for the separation was determining the correspondences of individual growth periods in all investigated curves. The correspondence between curves was determined by registering (alignment of salient or selected features) individual curves to the average growth curve in a given population. The registration was a two-step procedure. In the first step, we used the previously detected APV and calculated the average APV for the entire population, and subsequently, made the landmark registration of individual curves at this point. After registration, all curves were deformed so that their APV equaled the age of population average APV. As a numerical output (a record of the procedure) of the registration we received so-called *time-warping functions*, which determined the shift of the phase of each curve compared to the average. These *time-warping functions* again took the form of FDA splines (Figure 3). Subsequently, during the second phase of the registration, the *time-warping functions* were slightly optimized by a continuous registration, which no longer requires any other inputs (in the form of landmarks) apart from the growth curves themselves and are thus automatic from this point of view. Technical note: it can only be used to refine the previous landmark registration, as it is based on *local* (or fine) numerical optimization. Using solely this automatic local numerical optimization on raw data without previous substantial point registration, it would in many cases diverge from the original growth curves. The *time-warping functions* obtained during the registration can be used to deform the individual growth curves so that after their application the individual growth phases correspond (Figure 3).

**Figure 4:**
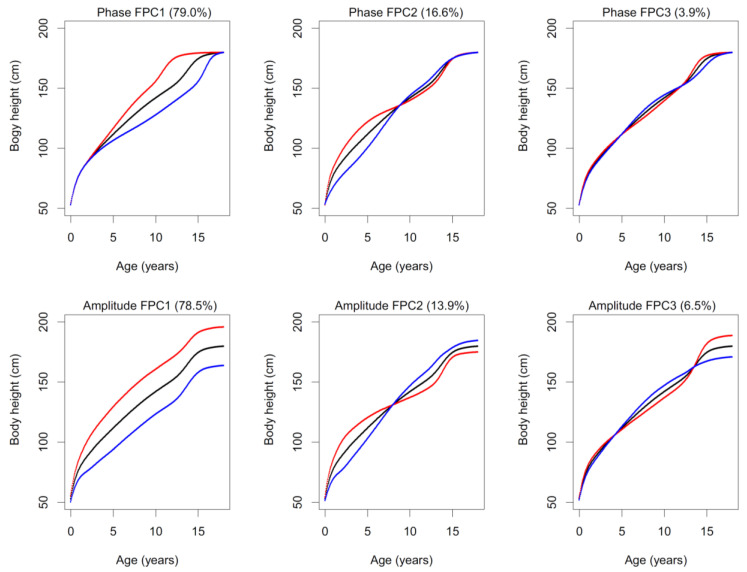
Functional Principal Component Analysis of the height data for boys in the Brno Growth Study: FPCA for phase (upper row) and FPCA for amplitude (lower row) variation—black line represents mean curve and color lines represent +3 Standard Deviations (red) and −3 Standard Deviations (blue); percentages of variance are indicated in parentheses; FPC1–3 are Functional Principal Component 1–3).

The FDA model is made up of two Functional Principal Components models (FPCA). The *first FPCA* is applied to the *inverse* time-warping functions (Figure 3) and describes the deformation of the average population curve to each original individual curve. After applying FPCA to a set of *inverse* time-warping functions, we obtained a generative model that was able to deform the average growth curve in terms of growth phase to new cases, created on the basis of specified model parameters. Given the statistical nature of the model, it can be assumed that if the parameters of the model are entered within certain/realistic intervals (range of plus or minus three standard deviations), then the resulting curves modeling new individuals will be biologically plausible (i.e., based on empirically recorded variation).The meaning of the first two main components of the model, i.e., of the main two harmonic functions of the model, is visualized in Figure 4. The second part of the FDA model involves also a Functional Principal Component Analysis (*second FPCA*) modeling of the amplitude of the curves. This is obtained by applying FPCA to the aligned growth curves; the resulting variation described by the second FPCA is illustrated in Figure 4 (complete set of components is visualized in Figure S6 for boys and Figure S7 for girls).

Generating a growth curve for a newly modeled case (several measurements of an individual subject) is based on the combination of both FPCA models. The parameters for the second FPCA model, describing the amplitude, are initially entered. The resulting curve has the same growth phase as the average curve but can differ in amplitude. Subsequently, a time-warping function is generated from the first FPCA model based on the entered parameters; the function will deform the obtained curve to adjust its phase, i.e., growth timing.

Based on the preceeding procedure, a completely new, artificially generated growth curve can be obtained; however, the curve always (a) represents a biologically possible human growth trajectory and (b) respects the statistical properties of the population data set. In other words, the generated curves respect both the possible shapes of the curves empirically recorded in the population and also the distribution of the shapes within the population. In this particular setting, we propose to use six parameters, i.e., harmonic functions (principal components), for modeling the growth phase, and the other six parameters for modeling the growth amplitude. The 12 components are thus used to generate model curves (for plots of variations in each of the 12 components see Supplementary Materials). Although most of the growth variation is described by the first three components of both FPCA models (Figure 4); preliminary testing indicated three other components of each FPCA, which could be interpreted as local effects and represented circa 1% of the variation (i.e., can be interpreted as noise). Nevertheless, they have a positive effect during registration/fitting (described further in text), where they increased the flexibility of the growth curve and reduced the residues between the measured values and the model curve.

### 2.4. Application of the Model to Newly Analyzed Cases

The proposed model can be used to fit new data, i.e., to interpolate or even to extrapolate the growth trajectory in a case with low number of measurements and/or with measurements distributed across variable time intervals, and in turn to estimate ATO and APV from the curve. The FPCA model described above generates artificial curves (a large number of curves) and the best fitting curve—the one best modeling the new data—is selected by means of an optimization procedure.

The registration/fitting of artificial curves to newly analyzed points is formulated as a problem of nonlinear least squares, which are solved as a local numerical optimization using the Levenberg–Marquardt algorithm [28]. During this optimization, the linking of two sets of residuals into one vector is minimized. The first set includes the residuals of heights at each observation, i.e., the differences between measured values and respective values in the generated FPCA model curves. This part of the optimization minimizes the distance of the model curve from the measured values and ensures that the generated model curve precisely intersects the measured values. The second set of residuals includes those of the parameters of the model. Since there is a normal probability distribution in the growth curves, the average curve, which corresponds to the zero parameters of the FPCA models, is also the most probable. Thus, the second set of residuals in the optimization serves to penalize less probable curves and increases the probability of the finally selected curve. During the registration, a compromise is sought between the exact intersection of the measured values and the population probability of the selected curve. This is important because the measured data naturally contain error or noise as measurements are not absolutely precise. Therefore, it is not expected that the resulting curve will exactly pass through the empirically measured points; this could lead to unlikely, unnatural deformations of the curve far removed from the real biological nature of growth.

**Figure 5:**
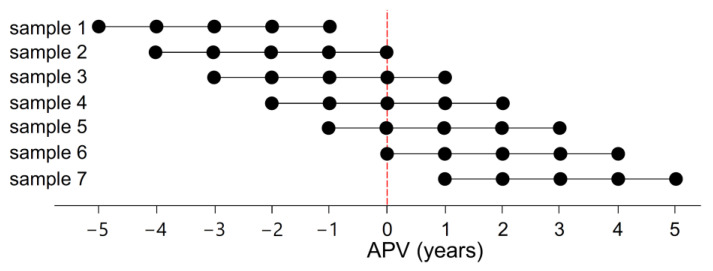
Sampling scheme of the testing samples 1–7. The individual five-point horizontal lines represent 7 permuted samples (all with five measurements one year apart) and the *x*-axis indicates the distance of each measurement from the population Age at Peak Velocity (APV) for the given sex. Thus, for example, the first measurement of sample 1 corresponds to an age 5 years before the APV and the fifth measurement of sample 1 corresponds to an age 1 year before the APV.

To demonstrate the applicability of the FPCA growth model, a permutation test of its performance on artificially prepared testing samples was performed. Measurement points from the reference sample were artificially selected to represent situations which may present themselves to pediatricians and sport anthropologists, i.e., a limited number of observations spanning various intervals and chronological ages during the interval of the growth spurt. Each test sample was represented by five measurements (with one year between adjacent points) in a different position relative to the average APV for the population by setting a specific range of chronological ages. From this selection, seven test samples differing by 1 year in each step resulted (Figure 5), from sample 1 in which the average of the last measurement ended 1 year before the average APV, to sample 4 in which the middle measurement average age equals APV, to sample 7 in which the average of the first measurement age was 1 year after the population average APV.

The testing round represented, in fact, a leave-one-out permutation procedure. In each testing round, each individual was separated from the rest of the sample, his/her data were limited/selected to the testing sample of 5 points, and on the remaining data (without the selected individual) the FPCA model was trained (established) using the above-mentioned procedure. At the same time, the testing sets were not comprised of the original raw measurements. Since children were measured at regular intervals spanning half a year of calendar age, ages of the raw measurement were not in the whole year difference relative to the average APV. The testing samples were, therefore, computed using B-spline base curve fitted to the full data and interpolation values on these curves for each required age. Since the B-splines are already smoothed models, we added a random Gaussian noise on *y*-axis (with parameters mean = 0 days and SD = 20 days) to each testing point to slightly de-regularize the imputing testing samples.

### 2.5. Comparison with an Alternative Fitting Method

To demonstrate the properties of the newly proposed approach, its results were compared with an analogically processed SITAR model [11] as available in the R-package sitar [29]. To provide a fair comparison with the new approach, the SITAR model was computed analogically as the leave-one-out procedure described above: first, the SITAR model was fitted on the full data, i.e., a longitudinal record of 39 measurements (of 166 boys or 166 girls), excluding (leaving-out) the tested case. When applied to the entire age range (0–18 years), the growth curve was variable in the first 3–5 years, and it was hard to find convergence in the model. Therefore, the age span was limited to a range from 6 to 18 years and the degrees of freedom of the B-spline were set to 30. In the second step, the model was used to fit the test data (5 points) of each test case to estimate the growth parameters.

In all test procedures, correlations and differences between reference values and estimates of growth milestones (APV, VPV, ATO, VTO) were used as criteria of the model’s performance. The differences were expressed in scatter plots (estimates plotted against references) and Bland-Altman plots of differences plotted by routines available in the R-package *BlandAltmanLeh* [30]. The standard deviations were also plotted relative to the mean values of all differences to visualize the overall differences between testing samples and both methods. Outliers and extremes in the estimates (i.e., cases incorrectly predicted) were detected and evaluated relative to their growth variations.

To test the effect of the shift in the coverage of APV within the testing sample (i.e., the effect of differences between testing samples on their estimates of population APV) a linear mixed effect model of the R-package *nlme* [31] was used, where D (difference between an estimate and reference computed as estimate minus reference) was the dependent variable, the testing sample (1–7) was the studied factor both at the population and individual level, and the data were grouped (random effect) by subjects. The mixed models were computed on the sample of all results and the effects of sex (males, females), method (FPCA, SITAR) and true APV on the estimate differences (D) were included.

## 3. Results

### 3.1. Description of the Source Sample

Complete data for height of all complete cases in the Brno Growth Study are illustrated separately for boys and girls in Figure 2 along with two examples of individual curves augmented with velocity and acceleration curves and the ATO and APV milestones. Individual distance curves for heights for all cases are available in Supplementary Materials. Descriptive statistics for ATO and APV, and the respective velocities at each milestone in the reference sample are summarized in Table 1. ATO was approximately 1.5 years earlier in girls compared to boys, but the decrease in growth velocity was not as pronounced in girls as in boys. Boys reached APV approximately 2 years later and peak velocities were, on average, higher in boys than in girls. The observed differences are consistent with the noted sex differences in the timing of the adolescent growth spurt and maximum growth velocities in height. This difference is also reflected in the differences in the growth curves for height. Descriptive plots and more comprehensive statistical descriptions of the BGS sample are also available in Supplementary Materials (Figure S4–S5, Tables S2-S5).

### 3.2. Functional Principal Component Analysis

Shape changes in the functional principal components of the first and the second FPCA are illustrated for boys in Figure 4, along with the percentages of explained variance. In both FPCAs, the first principal component extracted about 79% of variance in boys and the first three principal components explained more than 99% of variance in boys. Among girls, the corresponding estimate for the first principal component was 76%, while the first three principal components explained more than 95% of the variance (see Supplementary Materials). The FPCs thus extracted a substantial proportion of the original variation of the fitted B-splines. FPC1 represents the variation in growth timing (early through late) for most of the growth curve (except for the first 2–3 years). FPC2 represents a shift in timing of the trajectories of the younger and older part of the curve with change point at a prepubertal age; it is associated with major variation in the prepubertal decline in growth velocity. FPC3 represents the corresponding variation in the growth curve phase here divided into three periods with opposing timing trends. The amplitude PCA represents a similar pattern in the growth amplitude, i.e., size for a given age. Amplitude PC1 reflects a uniform shift in size almost across the entire growth trajectory. Amplitude PC2 represents opposite trends in size in the younger and older parts of the trajectory, and amplitude PC3 reflects the opposite changes in each third of the curve. The resulting PCs showed a similar pattern of variations in girls; the plots are available in Supplementary Materials.

### 3.3. Testing Results

**Figure 6:**
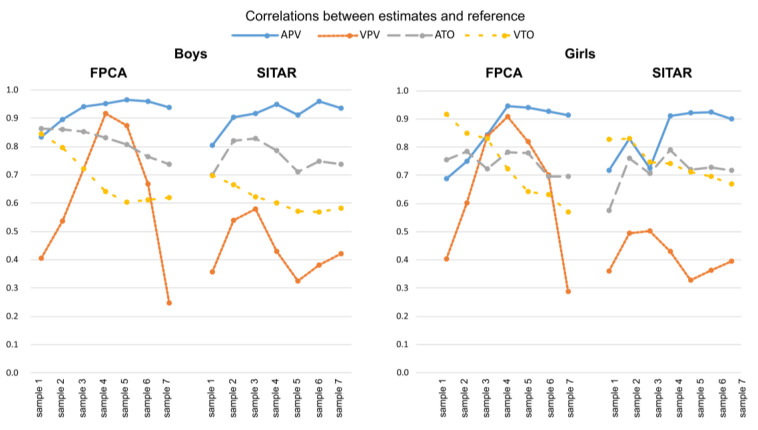
Sex-specific Pearson product moment correlations between estimates and the reference for growth milestones (APV, VPV, ATO, VTO) for each method and testing sample.

The complete set of descriptive statistics for all samples, methods and growth milestones are available in Supplementary Materials. Correlations between estimates and the reference for growth milestones (ATO, VTO, APV, VPV) are illustrated in Figure 6 (for individual values see Figures S8–S9). Correlations are higher for age estimates (APV, ATO) than for velocity estimates (VPV, VTO) among boys, while correlations are more variable among girls. Correlations between estimates of APV and the reference are high with both methods in each sex; the highest correlations are apparent in samples 4–6 for both methods. Variation in correlations between samples is greatest for VPV in samples 3, 4, and 5 for the FPCA method and samples 2, 3, and 4 for the SITAR method. The correlations with both methods are relatively comparable in all milestones except for estimated VPV, which showed the largest differences between methods in favor of FPCA method.

**Figure 7:**
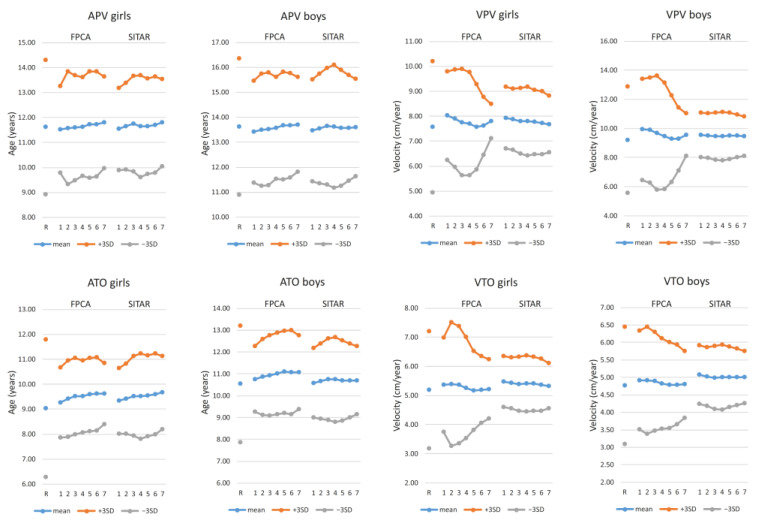
Descriptive statistics for estimates of the growth milestones (APV, VPV, ATO, VTO) for each method (FPCA, SITAR) among girls and boys and by testing sample (1–7) relative to the reference values (R) in the column of each plot.

**Figure 8:**
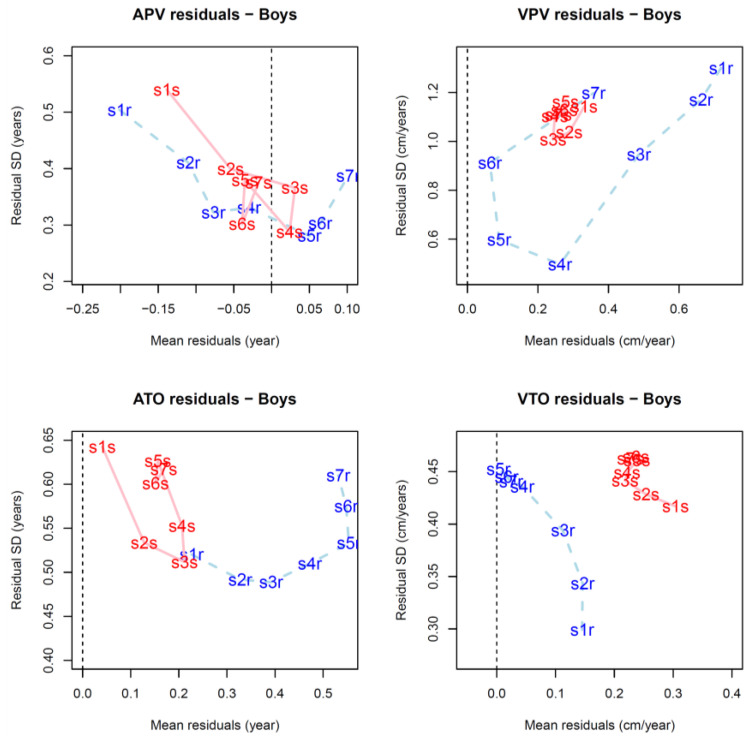
Residuals of the estimates of growth milestones between the FPCA (blue, dashed line) and SITAR (red, solid line) methods among boys; the standard deviations of residuals (*y*-axis) are plotted relative to the mean residuals (*x*-axis). Samples 1 to 7 are coded s1r to s7r (blue) for FPCA method and s1s to s7s (red) for SITAR method.

**Figure 9:**
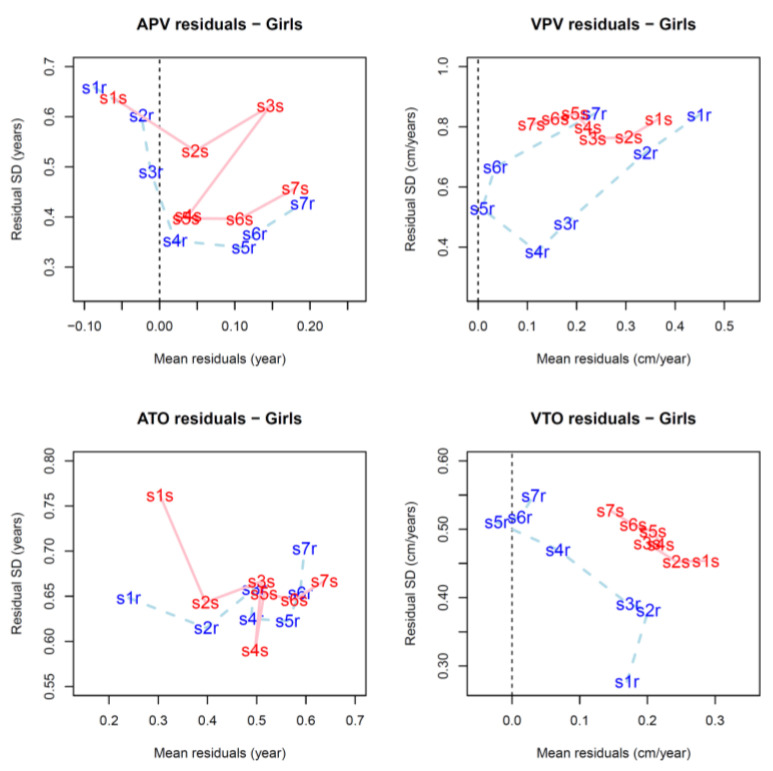
Residuals of the estimates of growth milestones between the FPCA (blue, dashed line) and SITAR (red, solid line) methods among girls; the standard deviations of residuals (*y*-axis) are plotted relative to the mean residuals (*x*-axis). Samples 1 to 7 are coded s1r to s7r (blue) for FPCA method and s1s to s7s (red) for SITAR method.

Descriptive statistics compared with the reference values are illustrated in Figure 7 (for numerical values see Tables S6–S9), while differences (D) between the estimates and reference values are summarized in Table 2 (for individual values in Bland-Altman plots see Figures S10–S11). The mean values are plotted relative to the residual standard deviations in Figure 8 and Figure 9, while the results of the linear mixed models are summarized in Table 3 and Table 4 and Tables S10–S12.

Average values of D for APV are close to zero for all testing samples and vary slightly, at most by about 0.2 year (see Figures S12–S13 for individual trends). For the FPCA method, there is a trend to slight underestimation in the low-numbered samples (1, 2, and 3) and overestimation in the high numbered samples. Standard deviations of the D for APV are also comparable for both methods and vary from 0.66 years (sample 1, girls FPCA method) to 0.28 years (sample 5, boys FPCA method). In general, there is a tendency for higher variance of D in girls than in boys (Table 2). Sample, sex and reference APV have significant single effects on the estimates, but method does not (Table 3). However, there are significant interactions between sample and method (i.e., significantly different performances of each method on different samples), and among method, sample and reference APV; significant variations are associated with differences in pubertal timing.

For ATO, overestimation is apparent in all samples and with both methods, spanning from 0.04 years (sample 1, boys, SITAR method) up to 0.64 years (sample 7, girls, SITAR method). The effects of all factors and most interactions are significant in the mixed model; the interaction between sample and reference ATO and among sample, sex, and reference ATO are exceptions (Table 4). By inference and contrary to the estimates of APV, the D values of ATO do not differ among the samples relative to reference ATO and sex (Table 4).

Both velocities (VPV and VTO) are generally overestimated by the two methods; for some samples (samples 5–6) the performance of FPCA method is better and the average estimates are much closer to the reference values than for the SITAR method (Figure 7). Mean values of the differences (D) resulting from the SITAR procedure are more similar among the different samples (e.g., VPV in boys in Figure 7), while those resulting from FPCA method are more variable. The differences between methods are also apparent in the results of the mixed model for VTO (see values of F-statistics for the factor of method and interactions with this factor in Tables S11–S12) but are less evident for the VPV results.

In general, both methods underestimate variation in all of the tested growth milestones. The tendencies are slight for ages (APV, ATO) and much higher for estimated velocities, and higher for the SITAR than for the FPCA method (Figure 7), i.e., standard deviations of VPV residuals are much lower for the SITAR than the FPCA method. While the reference value for the standard deviation of VTO was 0.67 cm/year for girls and 0.56 cm/year in boys, SITAR estimates (among samples) varied between 0.26 and 0.32 cm/year in girls and between 0.25 and 0.31 cm/year in boys which were in some samples less than one-half of the references value (see Supplementary Materials for detailed descriptive statistics of all variables). This reduction in variance is the most evident in estimates of VPV in boys computed by the SITAR method.

Variances of the random effects in the mixed effects models for D values of all tested variables are relatively small (Supplementary Materials). This applies both for the total random variance and the separately estimated intercept and slope variances (D against sample); in models for ATO and VTO, the slope variances are numerically zero. ICCs are moderate to high which indicates relatively similar estimates for all samples and high inter-individual differences in D values. The model for VPV is an exception; it has a relatively low intraclass correlation (ICC = 0.13) and a relatively high proportion of random variance (σ^2^ = 0.27) which indicates a high intra-individual influence of testing sample on the model performance.

## 4. Discussion

### 4.1. General Aspects of the Approach

The analysis followed the general idea of fitting a model to a complete (training) dataset, developing the model, and then applying the model to estimate growth milestones with new data. This idea is not new. Hermanussen and Meigen [32] for example, proposed an approach based on a discrete Ramsay and Bock [33] growth model separating phase and amplitude variation with principal components analysis [34]. Growth curves of the reference population were brought into correspondence using a shifting algorithm (Ramsay, Silverman 1997) and maximum likelihood principle and Hooke-Jeeves [35] algorithms with linear interpolation were used for regressing the model into an arbitrary set of measurements of an individual child.

In contrast to the previous approaches, the present analysis was focused on continuous data analysis. Growth curves of the reference population were described using splines, registered using landmark-based and continuous methods, and finally processed using functional principal component analysis (FPCA). The regression model was formulated as a non-linear least squares problem and optimized using the Levenberg–Marquardt solver. The performance of two methods was tested by fitting growth curves to 7 samples of sparse data (5-point by1 year distances) which differed in their relationship to the population mean APV. As the first step of the procedure with both methods, a model was trained on the complete dataset of full growth curves (0–18 years, 39 points), and subsequently was applied to the 5-point testing samples to fit and estimate growth milestones. It should be noted that with both the FPCA and SITAR methods the procedure was designed as leave-one-out approach, i.e., for each subject a new individually specific model was created applying reference data without the currently estimated subject. In other words, the subject was never included in the model by which his/her testing samples were estimated. Thus, all models were based on 166 full reference curves (both in boys and girls), against 167 for the total sample. The differences between the FPCA and SITAR methods were only in how the final model was created and fitted to the testing samples. With the SITAR method, the model curve represented an average B-spline of superimposed curves uniformly deformed to the data of the testing sample and the deformation was optimized by means of the Maximum Likelihood method (since sitar package uses the optimization method from *nlme* R-package [31]). With the FPCA method, the model represented a combination of 12 functional PCs (6 amplitude and 6 phase PCs) permuted and fitted to the testing samples by means of the Levenberg–Marquardt algorithm [28]. We did not follow the original SITAR application which would be applied only to each of the testing samples (i.e., sitar would load and be trained only on the 5-point data as a population set) since the testing samples were composed of many cases covering growth before or after APV, and not having an S-shape) and most of the attempts to fit the sitar model were not successful.

### 4.2. Comparison between FPCA and SITAR

Estimates of both methods were similar especially in mean values which varied close to the reference values. The APV estimates of the FPCA method changed with increasing order of the testing sample with the best approaching the reference in the middle order testing samples (3–5); this may have reflected the registration procedure in FDA—the curves were explicitly registered by means of individual APV values. This may be the reason why FPCA estimates were closest to the reference in samples subsuming population APV in the majority of cases (samples 3 to 5). FPCA method also overestimated ATO values more than the SITAR method. Both methods overestimated all mean values of growth velocities (except for VTO in sample 5 in girls estimated by FPCA method), which was, in contrast, higher for the SITAR than for the FPCA method. The SITAR method showed considerably more even estimates within all testing samples both for ages and velocities. This likely reflected the universal average B-spline method which did not satisfactorily address the whole variation range.

The decreased variation in estimates with both methods was a concern. The decrease was relatively moderate and similar for both methods for age estimates (APV, ATO), but the variation was generally much lower than in the reference values for velocity estimates (VPV, VTO); the latter was especially evident for the SITAR method. This may have been related to the use of one universal fitting B-spline curve which could not handle all variations in curve slopes during the most intensive interval of pubertal growth. If the true (reference) variation in the velocity of the most intensive growth interval would be higher compared to other parts of the growth curve, the uniform morphing of the entire curve applied in the SITAR procedure could not reflect the values of maximum growth velocities in a sufficient range (whole range of maximum slopes), despite the fact that it could perfectly detect APV. The lowest reduction in variation of velocities (both for VPV and VTO) was recorded for estimates with the FPCA method in the first four samples in both boys and girls, where the ranges of velocity estimates were almost as high as in the reference sample. Thus, for reliable detection of the whole range of velocities with the FPCA method, knowledge of the growth trajectory *before* the velocity point (acceleration part of curve) is more important than knowledge of the trajectory *after* the point (deceleration part of the curve).

### 4.3. Strengths of the Method and Comparisons with Alternative Approaches

Allowing for the preceding limitations, the tested approach—fitting trained models derived from full empirical curves to a small number of sparse data points—showed potentially applicable estimates, not only for the task of interpolation but also as a predictive means of extrapolation for future growth. Methods for prediction of future growth are based on a combination of chronological age at the time of measurement and biological age. Since X-ray based methods for the assessment of biological age are increasingly not possible outside of medical diagnostics, predicted estimates based on chronological age and anthropometric dimensions are developed [17,18]. When comparing the differences between predictions and true values of the Mirwald method (which requires chronological age, sitting height, estimated leg length, height and weight) with methods of the present study (both FPCA and SITAR) applied to sample 1 (i.e., 5 points with maximum age 1 year before population APV), the standard deviations for the differences are similar (Mirwald method: 0.49 years and 0.65 years for two samples of boys, and 0.49 years and 0.68 years for two samples of girls, FPCA method on sample 1: 0.50 years for BGS boys, and 0.66 years for BGS girls). Although there was a substantial difference in the inputs of the respective methods (5 height measurements in our tests and only one measurement point in Mirwald method), the latter incorporated several anthropometric dimensions and the proportion of estimated leg length to sitting height.

One challenge for new, computer intensive methods like those tested in this study is the possibility of using them for practical applications. Neither the computational procedure nor trained models can be easily shared, e.g., as a simple equation, and then simply applied by other members of the scientific community. Therefore, an on-line application based on the FPCA estimates accessible on the Internet was developed.

## 5. Conclusions

A method for description, interpolation and prediction of human postnatal growth trajectory was developed and tested. The principle of the approach was based on functional data analysis, training the functional principal component model on the full data set and the generation of artificial fitting curves by a combination of principal components, while at the same time respecting the natural variation in the shape of the human growth curve and optimizing the fit to the sparse data by means of the Levenberg–Marquardt algorithm. This FPCA procedure of generating, optimizing and fitting the model curve into sparse and non-numerous data provided comparable results with the SITAR method (applied in an identical leave-one-out procedure) when estimating ages at growth milestones (APV, ATO), but had more realistic results in terms of variance in estimates of growth velocities (VPV, VTO).

The FPCA procedure should be further tested on different data sets, especially for different populations. The computational nature of the procedure allows the method to be complemented and/or combined with data for indicators of biological age (skeletal age, stage of puberty) that could be used to refine the resulting estimates.

We also believe that our proposed method could have a more general application and could be applied not only to other growth processes in the human body, but also to other types of growth data (e.g., in demography or economics); however, a must-have input condition is always a sufficiently robust sample of complete, empirically recorded data, which will be used to train a model on which to generate artifactual/test curves.

## Figures and Tables

**Table 1:** Descriptive statistics for the parameters of the adolescent spurt in height—reference values—for girls and boys in the Brno Growth Study; units: years (for APV and ATO) and cm per year (for VPV and VTO).

			GIRLS						BOYS		
	n	Mean	sd	min	Max		n	Mean	sd	Min	Max
APV	167	11.61	0.90	9.09	13.75		167	13.61	0.91	10.95	16.57
VPV	167	7.57	0.88	5.19	10.80		167	9.21	1.22	6.15	11.96
ATO	167	9.03	0.92	6.4	11.23		167	10.54	0.89	7.99	13.02
VTO	167	5.19	0.67	3.26	7.23		167	4.77	0.56	3.49	6.31

**Table 2:** Descriptive statistics of the residuals (differences between estimates and reference values) of growth milestones (APV, VPV, ATO, VTO) for all testing samples (1–7), both sexes (girls, boys) and both tested methods (FPCA, SITAR); units of age estimates (APV, ATO) are years, units of velocity estimates are cm per year.

						GIRLS								BOYS			
				FPCA				SITAR				FPCA				SITAR	
			Mean	sd	Median		Mean	sd	Median		Mean	sd	Median		Mean	sd	Median
	sample 1		−0.09	0.66	−0.06		−0.06	0.64	−0.05		−0.20	0.50	−0.10		−0.14	0.54	−0.08
	sample 2		−0.02	0.60	−0.01		0.05	0.53	0.04		−0.11	0.41	−0.01		−0.05	0.40	−0.02
	sample 3		−0.01	0.49	0.03		0.15	0.62	0.07		−0.08	0.32	0.01		0.03	0.37	0.06
APV	sample 4		0.02	0.35	0.05		0.04	0.40	0.03		−0.03	0.33	0.03		0.02	0.29	0.04
	sample 5		0.11	0.34	0.09		0.04	0.40	0.05		0.05	0.28	0.08		−0.04	0.38	0.00
	sample 6		0.13	0.37	0.11		0.11	0.40	0.11		0.06	0.30	0.09		−0.04	0.30	−0.02
	sample 7		0.19	0.43	0.14		0.18	0.46	0.14		0.10	0.39	0.10		−0.02	0.37	−0.05
	sample 1		0.45	0.84	0.42		0.37	0.83	0.30		0.72	1.30	0.59		0.33	1.14	0.38
	sample 2		0.34	0.71	0.25		0.31	0.77	0.31		0.66	1.17	0.45		0.29	1.04	0.34
	sample 3		0.18	0.48	0.18		0.23	0.76	0.25		0.49	0.95	0.37		0.24	1.01	0.24
VPV	sample 4		0.12	0.38	0.15		0.22	0.80	0.25		0.26	0.50	0.30		0.25	1.10	0.19
	sample 5		0.01	0.53	0.11		0.20	0.84	0.18		0.09	0.60	0.27		0.28	1.16	0.24
	sample 6		0.03	0.67	0.15		0.16	0.83	0.15		0.06	0.91	0.27		0.28	1.13	0.23
	sample 7		0.24	0.84	0.29		0.11	0.81	0.14		0.36	1.20	0.51		0.26	1.11	0.26
	sample 1		0.24	0.65	0.28		0.30	0.76	0.31		0.23	0.52	0.23		0.04	0.64	0.06
	sample 2		0.40	0.61	0.29		0.40	0.64	0.38		0.33	0.49	0.27		0.13	0.53	0.09
	sample 3		0.49	0.66	0.42		0.51	0.67	0.45		0.39	0.49	0.33		0.21	0.51	0.13
ATO	sample 4		0.49	0.63	0.45		0.50	0.59	0.46		0.47	0.51	0.40		0.21	0.55	0.17
	sample 5		0.56	0.62	0.49		0.51	0.65	0.51		0.55	0.53	0.48		0.16	0.63	0.15
	sample 6		0.59	0.65	0.54		0.58	0.65	0.58		0.55	0.58	0.46		0.15	0.60	0.11
	sample 7		0.60	0.70	0.55		0.64	0.67	0.61		0.53	0.61	0.44		0.17	0.62	0.09
	sample 1		0.17	0.28	0.16		0.29	0.45	0.33		0.15	0.30	0.12		0.30	0.42	0.27
	sample 2		0.20	0.38	0.16		0.24	0.45	0.33		0.15	0.34	0.11		0.25	0.43	0.24
	sample 3		0.17	0.39	0.11		0.20	0.48	0.30		0.11	0.39	0.08		0.22	0.44	0.21
VTO	sample 4		0.07	0.47	0.07		0.22	0.48	0.28		0.04	0.44	0.02		0.22	0.45	0.24
	sample 5		−0.02	0.51	−0.01		0.21	0.50	0.28		0.00	0.45	0.00		0.24	0.46	0.29
	sample 6		0.01	0.52	0.01		0.18	0.51	0.27		0.02	0.45	0.04		0.24	0.46	0.26
	sample 7		0.03	0.55	0.05		0.15	0.53	0.22		0.02	0.44	0.05		0.23	0.46	0.26

**Table 3:** Analysis of Variance of the Linear Mixed Effects model for differences (D) between estimates and references values of Age at Peak Velocity (APV) with effects of sample (samp, 1–7), sex (sex, males, females), estimation method (met, FPCA, SITAR), and reference APV (apv.ref, age in years), including all interactions.

	numDF	denDF	F-Value	*p*-Value
(Intercept)	1	4330	17.5932	<0.0001
samp	1	4330	56.6251	<0.0001
met	1	4330	3.2439	0.07
sex	1	330	20.0942	<0.0001
apv.ref	1	330	735.1555	<0.0001
samp:met	1	4330	103.4955	<0.0001
samp:sex	1	4330	0.3127	0.6
met:sex	1	4330	6.2683	0.012
samp:apv.ref	1	4330	11.9666	0.0005
met:apv.ref	1	4330	9.5817	0.002
sex:apv.ref	1	330	17.0984	<0.0001
samp:met:sex	1	4330	11.7618	0.0006
samp:met:apv.ref	1	4330	1.5042	0.22
samp:sex:apv.ref	1	4330	5.9837	0.015
met:sex:apv.ref	1	4330	46.0603	<0.0001
samp:met:sex:apv.ref	1	4330	0.7064	0.4

**Table 4:** Analysis of Variance of the Linear Mixed Effects model for differences (D) between estimates and references values of Age at Take-off (ATO) with effects of sample (samp, 1–7), sex (sex, males, females), estimation method (met, FPCA, SITAR), and reference ATO (ato.ref, age in years), including all interactions.

	numDF	denDF	F-Value	*p*-Value
(Intercept)	1	4330	496.6572	<0.0001
samp	1	4330	159.4303	<0.0001
met	1	4330	676.2191	<0.0001
sex	1	330	31.8098	<0.0001
ato.ref	1	330	1110.021	<0.0001
samp:met	1	4330	76.7323	<0.0001
samp:sex	1	4330	6.9282	0.0085
met:sex	1	4330	773.0021	<0.0001
samp:ato.ref	1	4330	0.0824	0.8
met:ato.ref	1	4330	9.1492	0.0025
sex:ato.ref	1	330	17.4343	<0.0001
samp:met:sex	1	4330	44.7276	<0.0001
samp:met:ato.ref	1	4330	16.5127	<0.0001
samp:sex:ato.ref	1	4330	1.1218	0.3
met:sex:ato.ref	1	4330	20.0348	<0.0001
samp:met:sex:ato.ref	1	4330	20.7452	<0.0001

## Data Availability

The data presented in this study are available on request from the corresponding author. The data are not publicly available due to the authors of the study are only supervisors and are not at liberty to share the raw data. However, the data are available in Supplementary Materials in the form of plots and are being prepared for use in a freely available web application based on the methodological approaches of this paper.

## Supplementary Materials

The following are available online at https://www.mdpi.com/article/10.3390/children8100934/s1, Figure S1: Raw data for boys, Figure S2: Raw data for girls, Figure S3: Detection plots of growth milestones for all subjects, Figure S4: Descriptive plot of APV and VPV references, Figure S5: Descriptive plot of ATO and VTO references, Figure S6: FPCA for boys, Figure S7: FPCA for girls, Figure S8: Scatterplots of estimates against references of APV, Figure9: Scatterplots of estimates against references of ATO, Figure S10: Bland-Altman plots of APV, Figure S11: Bland-Altman plots of ATO, Figure S12: Individual differences in APV by testing samples, Figure S13: Individual differences in ATO by testing samples, Table S1: Test of differences in height between the tested sample and drop-out, Table S2: Descriptive statistics of the references and estimates of growth milestones by FPCA method for girls, Table S3: Descriptive statistics of the references and estimates of growth milestones by FPCA method for boys, Table S4: Descriptive statistics of the references and estimates of growth milestones by SITAR method for girls, Table S5: Descriptive statistics of the references and estimates of growth milestones by SITAR method for boys, Table S6: Descriptive statistics of D values by FPCA method for girls, Table S7: Descriptive statistics of D values by FPCA method for boys, Table S8: Descriptive statistics of D values by SITAR method for girls, Table S9: Descriptive statistics of D values by SITAR method for boys, Table S10: Results of four Mixed Effects Linear Models analysis of D-vales, Table S11: Analysis of Variance for differences (D) of VPV, Table S12: Analysis of Variance for differences (D) of VTO.
