# Supplementary material for: Estimating Growth in Height from Limited Longitudinal Growth Data Using Full-Curves Training Dataset: A Comparison of Two Procedures of Curve Optimization—Functional Principal Component Analysis and SITAR
Source: Children (Basel). 2021 Oct 18;8(10):934. doi: 10.3390/children8100934 (PMC8535004; doi:10.3390/children8100934)
Supplement: Supplementary file 1 [file children-08-00934-s001.zip › Suplementary_materials/Figure_S13_Individual_differences_by_maturation_age.pdf]

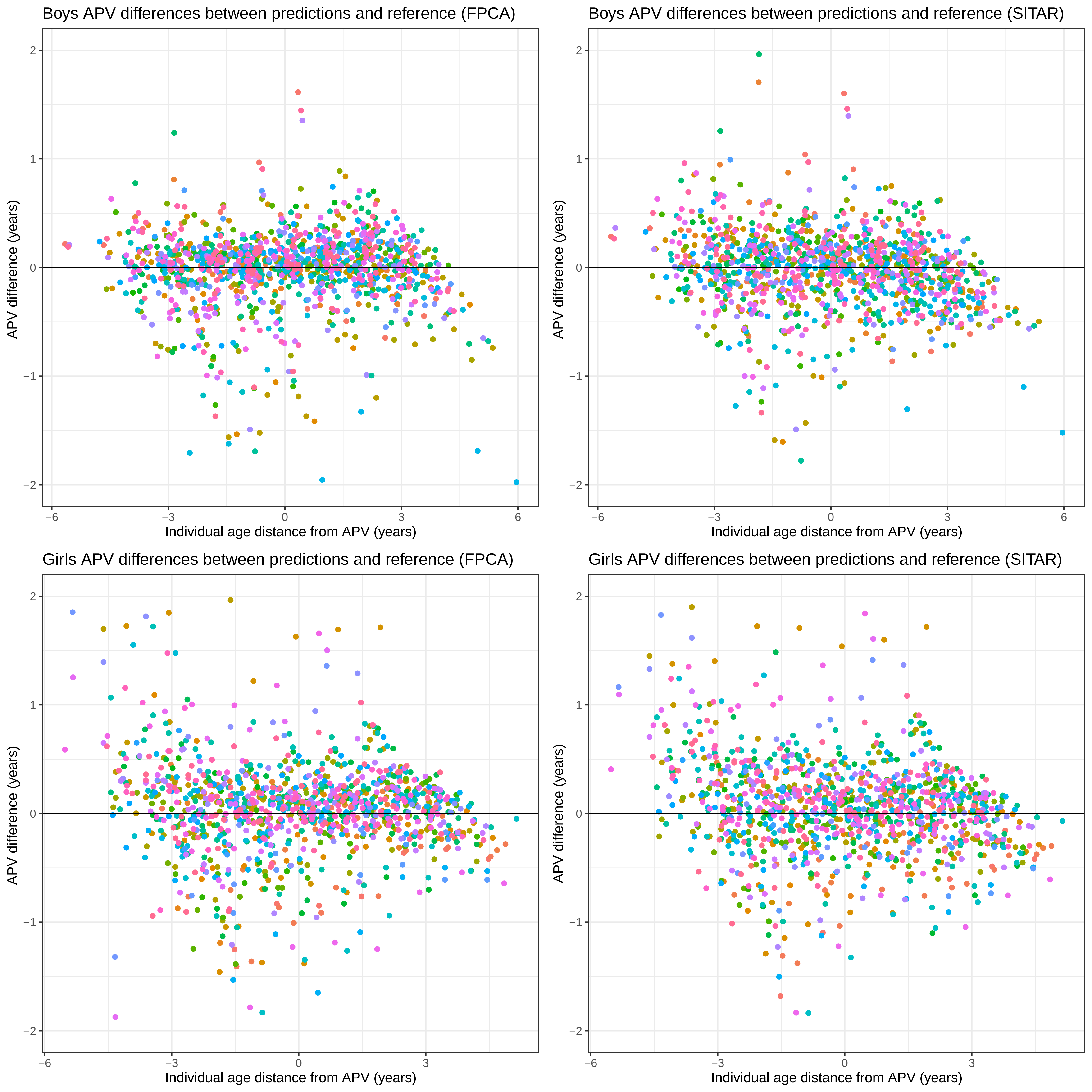

Boys APV differences between predictions and reference (FPCA)
Boys APV differences between predictions and reference (SITAR)
Girls APV differences between predictions and reference (FPCA)
Girls APV differences between predictions and reference (SITAR)
APV difference (years)
Individual age distance from APV (years)
2
1
0
−1
−2
−6
−3
0
3
6
