# Supplementary material for: Estimating Growth in Height from Limited Longitudinal Growth Data Using Full-Curves Training Dataset: A Comparison of Two Procedures of Curve Optimization—Functional Principal Component Analysis and SITAR
Source: Children (Basel). 2021 Oct 18;8(10):934. doi: 10.3390/children8100934 (PMC8535004; doi:10.3390/children8100934)
Supplement: Supplementary file 1 [file children-08-00934-s001.zip › Suplementary_materials/Supplementary_Figures_captions.pdf]

Supplementary Figures captions

Figure S1: Raw data for boys, line plot (points connected by segments) of 167 full cases of boys from Brno Growth Study used in the analysis.

Figure S2: Raw data for girls, line plot (points connected by segments) of 167 full cases of girls from Brno Growth Study used in the analysis.

Figure S3: Detection plots of growth milestones for all subjects, complete set of 334 plots of all full cases used in the analysis; crosses—raw measurement, light blue solid curve—distance curve (fitted B-spline), solid grey curve—velocity curve (1st derivative of the distance curve), dashed grey curve—acceleration curve 2nd derivative of the distance curve), dashed horizontal line – zero value for velocity and acceleration curves (at value 60 of the y-axis), blue vertical—Age at Take-off (ATO), red vertical—Age at Peak Velocity (APV), green vertical—age at maximum acceleration, violet vertical—age at reaching 98% of adult height; both velocity and acceleration curves were shifted by plus 60 and multiplied by 5 to be visible in one plot and readable against the same y-axis of the distance curve.

Figure S4: Descriptive plot of APV and VPV references, girls—red dots, boys—blue triangles.

Figure S5: Descriptive plot of ATO and VTO references, girls—red dots, boys—blue triangles.

Figure S6: FPCA for boys, Functional Principal Component Analyses of body height data with all 12 extracted principal components, black solid line—mean form (at 0,0 position of FPCA coordinate system), red and blue solid line – variation of curve form represented by the component (+/–3SD).

Figure S7: FPCA for girls, Functional Principal Component Analyses of body height data with all 12 extracted principal components, black solid line—mean form (at 0,0 position of FPCA coordinate system), red and blue solid line—variation of curve form represented by the component (+/–3SD).

Figure S8: Scatterplots of estimates against references of Age at Peak Velocity (APV), red dots—girls, blue triangles—boys, augmented with smoothed spline with 95%CI.

Figure S9: Scatterplots of estimates against references of Age at Take-off (ATO), red dots—girls, blue triangles—boys, augmented with smoothed spline with 95%CI.

Figure S10: Bland–Altman plots of APV, plotted for each sample (1–7) and sex separately with variants of full samples of differences and trimmed sample of differences.

Figure S11: Bland–Altman plots of ATO, plotted for each sample (1–7) and sex separately with variants of full samples of differences and trimmed sample of differences.

Figure S12: Individual differences in APV by testing samples, individual values of differences for each sample are connected by thin lines of different color for each individual, thick yellow line—mean values connected by line.

Figure S13: Individual differences in ATO by testing samples, individual values of differences for each sample are connected by thin lines of different color for each individual, thick yellow line—mean values connected by line.
