# Supplementary material for: Estimating Growth in Height from Limited Longitudinal Growth Data Using Full-Curves Training Dataset: A Comparison of Two Procedures of Curve Optimization—Functional Principal Component Analysis and SITAR
Source: Children (Basel). 2021 Oct 18;8(10):934. doi: 10.3390/children8100934 (PMC8535004; doi:10.3390/children8100934)
Supplement: Supplementary file 1 [file children-08-00934-s001.zip › Suplementary_materials/Supplementary_Tables.pdf]

Supplementary Tables

	Boys							
	Drop-out			Studied sample			Test of the difference	
	N	mean	sd	N	mean	sd	W	p-value
**at birth**	51	51.1	1.6	167	50.8	2.1	4414	0.69
**at age 1**	46	76.8	2.9	163	77.1	2.5	3446	0.40

	Girls							
	Drop-out			Studied sample			Test of the difference	
	N	mean	sd	N	mean	sd	W	p-value
**at birth**	60	49.5	2.1	167	50.0	2.0	4564	0.30
**at age 1**	57	74.7	2.6	162	75.4	2.5	4040	0.16

Table S1 Test of differences in body length/height between the tested sample and drop-out of the Brno Growth Study, for testing their differences Wilcoxon rank sum test with continuity correction was used.

variable	n	mean	sd	median	trimmed	min	max	range	skew	kurtosis
apv.reference	167	11.61	0.9	11.7	11.61	9.09	13.75	4.66	-0.18	-0.31
apv.fpca.s1	167	11.52	0.58	11.59	11.55	9.67	12.77	3.1	-0.46	-0.33
apv.fpca.s2	167	11.58	0.75	11.66	11.64	7.4	12.95	5.55	-1.76	6.94
apv.fpca.s3	167	11.59	0.7	11.63	11.61	9.57	13.31	3.74	-0.21	-0.14
apv.fpca.s4	167	11.63	0.66	11.75	11.65	9.66	13.17	3.51	-0.42	-0.21
apv.fpca.s5	167	11.72	0.71	11.77	11.74	9.66	13.44	3.79	-0.29	-0.17
apv.fpca.s6	167	11.73	0.7	11.81	11.74	9.98	13.69	3.71	-0.09	-0.39
apv.fpca.s7	167	11.8	0.61	11.79	11.79	10.57	13.7	3.13	0.14	-0.45
vpv.reference	167	7.57	0.88	7.54	7.57	5.19	10.8	5.62	0.2	0.74
vpv.fpca.s1	167	8.02	0.59	7.96	8	6.58	10.11	3.53	0.47	0.55
vpv.fpca.s2	167	7.91	0.65	7.87	7.9	6.21	10.61	4.4	0.41	1.04
vpv.fpca.s3	167	7.75	0.71	7.75	7.75	5.85	10.55	4.7	0.3	1.22
vpv.fpca.s4	167	7.69	0.69	7.66	7.69	5.86	10.28	4.42	0.25	1.05
vpv.fpca.s5	167	7.58	0.57	7.57	7.58	5.92	9.46	3.55	0.04	0.59
vpv.fpca.s6	167	7.61	0.39	7.59	7.61	6.34	9.07	2.73	-0.19	2.12
vpv.fpca.s7	167	7.81	0.23	7.77	7.79	7.28	9.18	1.9	1.63	7.05
ato.reference	167	9.03	0.92	9.11	9.03	6.4	11.23	4.82	-0.09	-0.38
ato.fpca.s1	167	9.26	0.47	9.37	9.31	7.31	10	2.69	-1.12	1.67
ato.fpca.s2	167	9.42	0.51	9.46	9.45	7.22	10.37	3.14	-0.99	2.53
ato.fpca.s3	167	9.52	0.51	9.51	9.52	7.91	10.79	2.88	-0.19	0.54
ato.fpca.s4	167	9.51	0.48	9.58	9.54	7.92	10.69	2.77	-0.51	0.08
ato.fpca.s5	167	9.59	0.49	9.59	9.59	8.12	10.68	2.56	-0.13	-0.18
ato.fpca.s6	167	9.61	0.49	9.63	9.61	8.33	10.89	2.56	0.05	-0.22
ato.fpca.s7	167	9.62	0.41	9.65	9.61	8.77	10.9	2.13	0.23	-0.13
vto.reference	167	5.19	0.67	5.06	5.17	3.26	7.23	3.97	0.35	0.32
vto.fpca.s1	167	5.36	0.54	5.27	5.32	4.09	7.87	3.78	1.04	2.44
vto.fpca.s2	167	5.39	0.71	5.27	5.33	3.92	8.48	4.56	1.28	3.21
vto.fpca.s3	167	5.36	0.67	5.25	5.33	3.9	7.39	3.49	0.5	0.25
vto.fpca.s4	167	5.26	0.58	5.24	5.24	3.83	6.92	3.09	0.25	0.05
vto.fpca.s5	167	5.17	0.45	5.19	5.16	3.95	6.55	2.6	0.1	-0.15
vto.fpca.s6	167	5.2	0.38	5.19	5.2	4.25	6.21	1.96	0.1	-0.42
vto.fpca.s7	167	5.22	0.34	5.25	5.22	4.38	6.02	1.64	0.07	-0.38

Table S2 Girls: Descriptive statistics of the reference values of all milestones and their estimates by FPCA method for each testing sample (s1–s7) separately.

variable	n	mean	sd	median	trimmed	min	max	range	skew	kurtosis
apv.reference	167	13.61	0.91	13.62	13.59	10.95	16.57	5.62	0.09	0.76
apv.fpca.s1	167	13.41	0.68	13.5	13.44	11.16	14.76	3.6	-0.62	0.47
apv.fpca.s2	167	13.5	0.75	13.55	13.51	11.15	15.09	3.95	-0.36	0.16
apv.fpca.s3	167	13.53	0.75	13.55	13.51	11.19	15.48	4.29	0.05	0.77
apv.fpca.s4	167	13.58	0.68	13.65	13.6	11.19	14.87	3.68	-0.52	0.52
apv.fpca.s5	167	13.66	0.72	13.69	13.67	11.24	15.42	4.18	-0.3	0.54
apv.fpca.s6	167	13.67	0.7	13.68	13.67	11.72	15.43	3.71	-0.02	-0.1
apvfpca.s7	167	13.71	0.63	13.71	13.7	12.41	15.22	2.81	0.08	-0.65
vpv.ref	167	9.21	1.22	9.21	9.25	6.15	11.96	5.81	-0.17	-0.34
vpv.fpca.s1	167	9.93	1.16	9.8	9.92	7.32	13.07	5.75	0.17	-0.21
vpv.fpca.s2	167	9.88	1.21	9.84	9.88	7.16	13.01	5.85	0.01	-0.38
vpv.fpca.s3	167	9.7	1.31	9.83	9.72	6.57	12.68	6.11	-0.13	-0.32
vpv.fpca.s4	167	9.48	1.22	9.53	9.52	5.78	12.73	6.94	-0.26	-0.21
vpv.fpca.s5	167	9.3	0.99	9.25	9.32	6.28	12.27	5.99	-0.09	0.25
vpv.fpca.s6	167	9.28	0.72	9.32	9.27	6.95	11.66	4.7	0.07	0.9
vpv.fpca.s7	167	9.57	0.49	9.52	9.56	7.8	11.8	4	0.44	2.85
ato.reference	167	10.54	0.89	10.52	10.54	7.99	13.02	5.02	-0.07	0.27
ato.fpca.s1	167	10.76	0.5	10.88	10.8	9.15	11.59	2.44	-0.76	0.13
ato.fpca.s2	167	10.86	0.58	10.9	10.88	9.16	12.18	3.01	-0.35	-0.16
ato.fpca.s3	167	10.93	0.61	10.92	10.93	9.3	12.63	3.33	0.03	-0.15
ato.fpca.s4	167	11.01	0.62	10.98	10.99	9.43	12.76	3.33	0.3	0.03
ato.fpca.s5	167	11.09	0.63	11.01	11.06	9.38	12.84	3.45	0.41	0.32
ato.fpca.s6	167	11.08	0.64	11.01	11.04	9.53	13.04	3.51	0.66	0.68
ato.fpca.s7	167	11.07	0.56	11.02	11.03	9.95	12.84	2.88	0.7	0.62
vto.reference	167	4.77	0.56	4.83	4.77	3.49	6.31	2.82	0.07	-0.32
vto.fpca.s1	167	4.92	0.47	4.87	4.9	4.01	7.04	3.03	1.02	2.86
vto.fpca.s2	167	4.92	0.51	4.89	4.9	3.8	6.87	3.08	0.71	1.71
vto.fpca.s3	167	4.89	0.47	4.87	4.88	3.69	6.49	2.8	0.41	1.09
vto.fpca.s4	167	4.82	0.43	4.8	4.82	3.55	6.4	2.85	0.17	1.82
vto.fpca.s5	167	4.78	0.41	4.75	4.76	3.67	6.45	2.77	0.59	2.7
vto.fpca.s6	167	4.79	0.38	4.76	4.78	3.66	6.06	2.4	0.3	1.25
vto.fpca.s7	167	4.8	0.32	4.77	4.79	3.97	5.66	1.69	0.24	0.1

Table S3 Boys: Descriptive statistics of the reference values of all milestones and their estimates by FPCA method for each testing sample (s1–s7) separately.

variable	n	mean	sd	median	trimmed	min	max	range	skew	kurtosis
apv.reference	167	11.61	0.9	11.7	11.61	9.09	13.75	4.66	-0.18	-0.31
apv.sitar.s1	167	11.54	0.55	11.64	11.59	9.49	12.66	3.17	-0.81	0.43
apv.sitar.s2	167	11.65	0.58	11.74	11.68	9.9	12.9	3	-0.49	-0.24
apv.sitar.s3	167	11.75	0.64	11.77	11.76	10.08	13.1	3.02	-0.15	-0.53
apv.sitar.s4	167	11.64	0.68	11.73	11.65	9.66	13.39	3.73	-0.21	-0.01
apv.sitar.s5	167	11.64	0.64	11.76	11.67	9.8	12.93	3.13	-0.4	-0.37
apv.sitar.s6	167	11.71	0.64	11.77	11.72	10.13	13.38	3.25	-0.07	-0.53
apv.sitar.s7	167	11.79	0.58	11.78	11.77	10.65	13.68	3.03	0.33	-0.28
vpv.reference	167	7.57	0.88	7.54	7.57	5.19	10.8	5.62	0.2	0.74
vpv.sitar.s1	167	7.94	0.41	7.91	7.92	7.09	9.46	2.38	0.59	0.18
vpv.sitar.s2	167	7.88	0.41	7.85	7.86	6.98	9.23	2.25	0.5	-0.05
vpv.sitar.s3	167	7.81	0.44	7.78	7.79	6.85	9.12	2.26	0.34	-0.45
vpv.sitar.s4	167	7.8	0.46	7.77	7.78	6.76	9.07	2.31	0.25	-0.36
vpv.sitar.s5	167	7.77	0.43	7.73	7.75	6.74	8.99	2.25	0.33	-0.4
vpv.sitar.s6	167	7.73	0.42	7.69	7.72	6.69	8.77	2.09	0.1	-0.57
vpv.sitar.s7	167	7.68	0.38	7.68	7.69	6.72	8.46	1.74	-0.2	-0.55
ato.reference	167	9.03	0.92	9.11	9.03	6.4	11.23	4.82	-0.09	-0.38
ato.sitar.s1	167	9.33	0.44	9.43	9.37	7.62	10.23	2.61	-0.92	0.68
ato.sitar.s2	167	9.42	0.47	9.52	9.45	7.99	10.45	2.46	-0.49	-0.25
ato.sitar.s3	167	9.53	0.53	9.57	9.54	8.16	10.65	2.49	-0.15	-0.5
ato.sitar.s4	167	9.52	0.57	9.6	9.53	7.78	10.94	3.16	-0.27	0.1
ato.sitar.s5	167	9.54	0.54	9.64	9.57	7.92	10.64	2.71	-0.46	-0.32
ato.sitar.s6	167	9.6	0.54	9.66	9.61	8.2	11.03	2.83	-0.11	-0.49
ato.sitar.s7	167	9.66	0.49	9.67	9.65	8.67	11.3	2.63	0.3	-0.24
vto.reference	167	5.19	0.67	5.06	5.17	3.26	7.23	3.97	0.35	0.32
vto.sitar.s1	167	5.48	0.29	5.45	5.46	4.88	6.56	1.68	0.62	0.21
vto.sitar.s2	167	5.43	0.29	5.41	5.41	4.8	6.39	1.58	0.51	-0.06
vto.sitar.s3	167	5.39	0.31	5.37	5.37	4.71	6.31	1.59	0.35	-0.45
vto.sitar.s4	167	5.41	0.32	5.39	5.4	4.68	6.3	1.62	0.26	-0.36
vto.sitar.s5	167	5.4	0.31	5.36	5.38	4.67	6.25	1.57	0.34	-0.4
vto.sitar.s6	167	5.37	0.3	5.34	5.36	4.64	6.1	1.46	0.12	-0.57
vto.sitar.s7	167	5.33	0.26	5.33	5.34	4.66	5.88	1.23	-0.19	-0.55

Table S4 Girls: Descriptive statistics of the reference values of all milestones and their estimates by SITAR method for each testing sample (s1–s7) separately.

variable	n	mean	sd	median	trimmed	min	max	range	skew	kurtosis
apv.reference	167	13.61	0.91	13.62	13.59	10.95	16.57	5.62	0.09	0.76
apv.sitar.s1	167	13.47	0.68	13.56	13.49	11.23	15.04	3.81	-0.53	0.63
apv.sitar.s2	167	13.55	0.73	13.59	13.57	11.22	15.24	4.02	-0.35	0.31
apv.sitar.s3	167	13.64	0.78	13.61	13.63	11.11	15.57	4.47	-0.06	0.36
apv.sitar.s4	167	13.63	0.82	13.62	13.62	11.1	15.85	4.75	0.1	0.66
apv.sitar.s5	167	13.57	0.77	13.56	13.56	11.25	16.04	4.79	0.16	0.82
apv.sitar.s6	167	13.57	0.71	13.55	13.55	11.77	15.48	3.71	0.21	-0.08
apv.sitar.s7	167	13.59	0.65	13.51	13.55	12.45	15.46	3.01	0.47	-0.35
vpv.reference	167	9.21	1.22	9.21	9.25	6.15	11.96	5.81	-0.17	-0.34
vpv.sitar.s1	167	9.54	0.51	9.49	9.53	8.36	11.25	2.89	0.51	0.68
vpv.sitar.s2	167	9.5	0.51	9.46	9.49	8.36	11.21	2.85	0.5	0.71
vpv.sitar.s3	167	9.46	0.54	9.41	9.45	8.18	11.29	3.11	0.36	0.62
vpv.sitar.s4	167	9.46	0.56	9.46	9.46	8.03	11.29	3.26	0.17	0.58
vpv.sitar.s5	167	9.49	0.53	9.48	9.49	7.92	11.16	3.24	0.06	0.67
vpv.sitar.s6	167	9.49	0.49	9.49	9.49	8.28	10.74	2.46	-0.04	-0.2
vpv.sitar.s7	167	9.47	0.45	9.51	9.49	8.29	10.35	2.06	-0.28	-0.45
ato.reference	167	10.54	0.89	10.52	10.54	7.99	13.02	5.02	-0.07	0.27
ato.sitar.s1	167	10.58	0.53	10.66	10.6	8.83	11.72	2.89	-0.64	0.75
ato.sitar.s2	167	10.66	0.57	10.67	10.68	8.75	11.96	3.21	-0.41	0.32
ato.sitar.s3	167	10.75	0.62	10.7	10.74	8.65	12.24	3.59	-0.1	0.42
ato.sitar.s4	167	10.74	0.65	10.74	10.73	8.65	12.47	3.83	0.05	0.74
ato.sitar.s5	167	10.69	0.61	10.69	10.69	8.77	12.61	3.84	0.1	0.82
ato.sitar.s6	167	10.69	0.56	10.66	10.68	9.18	12.19	3.01	0.17	-0.07
ato.sitar.s7	167	10.7	0.52	10.65	10.68	9.71	12.18	2.46	0.46	-0.34
vto.reference	167	4.77	0.56	4.83	4.77	3.49	6.31	2.82	0.07	-0.32
vto.sitar.s1	167	5.08	0.28	5.05	5.07	4.44	6.03	1.59	0.56	0.81
vto.sitar.s2	167	5.03	0.28	5	5.02	4.38	6.02	1.63	0.53	0.85
vto.sitar.s3	167	4.99	0.3	4.97	4.99	4.28	6.05	1.77	0.39	0.79
vto.sitar.s4	167	5	0.31	4.99	4.99	4.21	6.05	1.84	0.21	0.73
vto.sitar.s5	167	5.01	0.29	5	5.01	4.15	5.97	1.82	0.11	0.78
vto.sitar.s6	167	5.01	0.27	5.01	5.01	4.36	5.74	1.38	0	-0.15
vto.sitar.s7	167	5	0.25	5.02	5.01	4.36	5.48	1.12	-0.26	-0.44

Table S5 Boys: Descriptive statistics of the reference values of all milestones and their estimates by SITAR method for each testing sample (s1–s7) separately.

variable	n	mean	sd	median	trimmed	min	max	range	skew	kurtosis
apv.fpca.s1	167	-0.09	0.66	-0.06	-0.09	-1.83	1.85	3.68	0.14	0.57
apv.fpca.s2	167	-0.02	0.6	-0.01	-0.04	-1.87	2.19	4.07	0.42	1.97
apv.fpca.s3	167	-0.01	0.49	0.03	-0.01	-1.65	2.36	4.01	1.01	7.18
apv.fpca.s4	167	0.02	0.35	0.05	0.03	-1.25	1.96	3.21	0.45	6.99
apv.fpca.s5	167	0.11	0.34	0.09	0.09	-0.73	2.53	3.26	2.66	16.41
apv.fpca.s6	167	0.13	0.37	0.11	0.1	-0.7	2.67	3.38	2.49	14.47
apv.fpca.s7	167	0.19	0.43	0.14	0.15	-0.64	2.6	3.24	1.85	6.88
vpv.fpca.s1	167	0.45	0.84	0.42	0.44	-2.03	2.56	4.59	0	0.27
vpv.fpca.s2	167	0.34	0.71	0.25	0.31	-1.63	2.33	3.96	0.3	0.22
vpv.fpca.s3	167	0.18	0.48	0.18	0.16	-2	1.8	3.81	0.08	3.3
vpv.fpca.s4	167	0.12	0.38	0.15	0.15	-1.6	0.84	2.44	-1.52	4.58
vpv.fpca.s5	167	0.01	0.53	0.11	0.07	-2.49	0.91	3.4	-1.62	3.79
vpv.fpca.s6	167	0.03	0.67	0.15	0.1	-3.27	1.49	4.77	-1.25	3.12
vpv.fpca.s7	167	0.24	0.84	0.29	0.27	-3.09	2.38	5.47	-0.55	0.97
ato.fpca.s1	167	0.24	0.65	0.28	0.25	-1.8	2.25	4.06	-0.17	0.89
ato.fpca.s2	167	0.4	0.61	0.29	0.37	-1.25	2.25	3.51	0.46	0.49
ato.fpca.s3	167	0.49	0.66	0.42	0.45	-1.47	2.8	4.27	0.62	0.98
ato.fpca.s4	167	0.49	0.63	0.45	0.47	-1.22	2.26	3.48	0.26	0.23
ato.fpca.s5	167	0.56	0.62	0.49	0.54	-0.89	2.5	3.39	0.42	0.21
ato.fpca.s6	167	0.59	0.65	0.54	0.56	-0.81	2.67	3.48	0.41	0.15
ato.fpca.s7	167	0.6	0.7	0.55	0.58	-1	2.67	3.68	0.3	0.04
vto.fpca.s1	167	0.17	0.28	0.16	0.17	-0.46	0.88	1.34	0.08	-0.5
vto.fpca.s2	167	0.2	0.38	0.16	0.16	-0.53	2.7	3.23	2.38	11.47
vto.fpca.s3	167	0.17	0.39	0.11	0.15	-0.83	1.93	2.77	0.87	2.14
vto.fpca.s4	167	0.07	0.47	0.07	0.07	-1.21	1.48	2.7	-0.02	0.16
vto.fpca.s5	167	-0.02	0.51	-0.01	-0.01	-1.54	1.56	3.1	-0.2	0.44
vto.fpca.s6	167	0.01	0.52	0.01	0.02	-1.49	1.54	3.03	-0.1	0.25
vto.fpca.s7	167	0.03	0.55	0.05	0.04	-1.57	1.56	3.13	-0.13	0.23

Table S6 Girls: Descriptive statistics of D values (differences between FPCA estimates and reference values) of all milestones for each testing sample (s1–s7) separately.

variable	n	mean	sd	median	trimmed	min	max	range	skew	kurtosis
apv.fpca.s1	167	-0.2	0.5	-0.1	-0.15	-2.47	0.78	3.25	-1.34	2.69
apv.fpca.s2	167	-0.11	0.41	-0.01	-0.06	-1.96	1.24	3.2	-1.26	3.6
apv.fpca.s3	167	-0.08	0.32	0.01	-0.03	-1.42	0.7	2.12	-1.73	4.23
apv.fpca.s4	167	-0.03	0.33	0.03	0.01	-2.85	0.58	3.42	-4.3	30.88
apv.fpca.s5	167	0.05	0.28	0.08	0.07	-2.03	0.63	2.67	-2.56	16.86
apv.fpca.s6	167	0.06	0.3	0.09	0.08	-1.69	0.97	2.65	-1.09	6.17
apv.fpca.s7	167	0.1	0.39	0.1	0.1	-1.98	1.61	3.59	-0.27	6.23
vpv.fpca.s1	167	0.72	1.3	0.59	0.66	-3.11	5.27	8.39	0.57	1.05
vpv.fpca.s2	167	0.66	1.17	0.45	0.55	-3.4	5.57	8.97	1.1	3.45
vpv.fpca.s3	167	0.49	0.95	0.37	0.37	-1.59	6.1	7.69	2.84	12.79
vpv.fpca.s4	167	0.26	0.5	0.3	0.32	-2.74	1.02	3.76	-2.79	12.63
vpv.fpca.s5	167	0.09	0.6	0.27	0.17	-2.34	1.22	3.56	-1.56	2.85
vpv.fpca.s6	167	0.06	0.91	0.27	0.11	-2.47	2.24	4.71	-0.55	-0.1
vpv.fpca.s7	167	0.36	1.2	0.51	0.37	-2.22	3.05	5.27	-0.13	-0.78
ato.fpca.s1	167	0.23	0.52	0.23	0.23	-1.55	2.35	3.9	0.05	2.89
ato.fpca.s2	167	0.33	0.49	0.27	0.3	-1.13	2.41	3.54	0.82	2.46
ato.fpca.s3	167	0.39	0.49	0.33	0.36	-0.6	2.46	3.06	0.89	1.93
ato.fpca.s4	167	0.47	0.51	0.4	0.44	-1.08	2.57	3.66	0.8	1.97
ato.fpca.s5	167	0.55	0.53	0.48	0.51	-0.5	2.65	3.15	0.88	1.15
ato.fpca.s6	167	0.55	0.58	0.46	0.51	-0.55	2.59	3.14	0.72	0.49
ato.fpca.s7	167	0.53	0.61	0.44	0.49	-0.75	2.61	3.36	0.63	0.32
vto.fpca.s1	167	0.15	0.3	0.12	0.13	-0.55	1.6	2.15	1.1	3.54
vto.fpca.s2	167	0.15	0.34	0.11	0.12	-0.68	1.77	2.45	1.17	3.72
vto.fpca.s3	167	0.11	0.39	0.08	0.1	-1	1.39	2.39	0.35	0.79
vto.fpca.s4	167	0.04	0.44	0.02	0.04	-1.22	1.34	2.56	0.02	0.41
vto.fpca.s5	167	0	0.45	0	0	-1.28	1.38	2.66	-0.02	0.32
vto.fpca.s6	167	0.02	0.45	0.04	0.02	-1.25	1.12	2.37	-0.19	-0.12
vto.fpca.s7	167	0.02	0.44	0.05	0.04	-1.2	1.12	2.32	-0.29	-0.27

Table S7 Boys: Descriptive statistics of D values (differences between FPCA estimates and reference values) of all milestones for each testing sample (s1–s7) separately.

variable	n	mean	sd	median	trimmed	min	max	range	skew	kurtosis
apv.sitar.s1	167	-0.06	0.64	-0.05	-0.05	-1.84	1.45	3.29	-0.19	0.2
apv.sitar.s2	167	0.05	0.53	0.04	0.03	-1.33	1.9	3.23	0.44	1.32
apv.sitar.s3	167	0.15	0.62	0.07	0.08	-0.93	3.8	4.73	2.84	13
apv.sitar.s4	167	0.04	0.4	0.03	0.03	-1.05	2.61	3.66	1.67	10.4
apv.sitar.s5	167	0.04	0.4	0.05	0.03	-1.1	2.19	3.29	0.86	5.71
apv.sitar.s6	167	0.11	0.4	0.11	0.08	-0.75	2.43	3.18	1.6	7.43
apv.sitar.s7	167	0.18	0.46	0.14	0.13	-0.61	2.63	3.23	1.77	5.66
vpv.sitar.s1	167	0.37	0.83	0.3	0.35	-2.18	2.66	4.85	0.14	0.22
vpv.sitar.s2	167	0.31	0.77	0.31	0.31	-2.21	2.39	4.61	-0.03	0.29
vpv.sitar.s3	167	0.23	0.76	0.25	0.25	-2.42	2.06	4.48	-0.33	0.35
vpv.sitar.s4	167	0.22	0.8	0.25	0.23	-2.64	2.59	5.22	-0.17	0.58
vpv.sitar.s5	167	0.2	0.84	0.18	0.2	-2.68	2.58	5.26	-0.09	0.52
vpv.sitar.s6	167	0.16	0.83	0.15	0.17	-2.65	2.38	5.04	-0.17	0.46
vpv.sitar.s7	167	0.11	0.81	0.14	0.12	-2.73	2.23	4.96	-0.23	0.49
ato.sitar.s1	167	0.3	0.76	0.31	0.32	-1.9	2	3.9	-0.25	-0.36
ato.sitar.s2	167	0.4	0.64	0.38	0.39	-1.59	1.99	3.58	0.01	0.05
ato.sitar.s3	167	0.51	0.67	0.45	0.46	-1.19	3.62	4.81	1.2	3.64
ato.sitar.s4	167	0.5	0.59	0.46	0.47	-1.21	2.25	3.46	0.28	0.51
ato.sitar.s5	167	0.51	0.65	0.51	0.51	-1.3	2.39	3.69	0.07	0.43
ato.sitar.s6	167	0.58	0.65	0.58	0.56	-1.05	2.62	3.67	0.23	0.35
ato.sitar.s7	167	0.64	0.67	0.61	0.61	-0.91	2.77	3.68	0.38	0.19
vto.sitar.s1	167	0.29	0.45	0.33	0.29	-1.1	1.64	2.74	-0.03	0.34
vto.sitar.s2	167	0.24	0.45	0.33	0.26	-1.18	1.55	2.73	-0.29	0.34
vto.sitar.s3	167	0.2	0.48	0.3	0.22	-1.36	1.58	2.94	-0.45	0.67
vto.sitar.s4	167	0.22	0.48	0.28	0.24	-1.31	1.62	2.92	-0.34	0.61
vto.sitar.s5	167	0.21	0.5	0.28	0.22	-1.34	1.71	3.04	-0.24	0.49
vto.sitar.s6	167	0.18	0.51	0.27	0.19	-1.41	1.74	3.15	-0.23	0.55
vto.sitar.s7	167	0.15	0.53	0.22	0.16	-1.55	1.76	3.31	-0.28	0.61

Table S8 Girls: Descriptive statistics of D values (differences between SITAR estimates and reference values) of all milestones for each testing sample (s1–s7) separately.

variable	n	mean	sd	median	trimmed	min	max	range	skew	kurtosis
apv.sitar.s1	167	-0.14	0.54	-0.08	-0.09	-2.54	0.99	3.53	-1.09	2.37
apv.sitar.s2	167	-0.05	0.4	-0.02	-0.04	-2.02	1.26	3.27	-0.72	3.65
apv.sitar.s3	167	0.03	0.37	0.06	0.03	-1.3	1.96	3.27	0.88	6.7
apv.sitar.s4	167	0.02	0.29	0.04	0.03	-0.76	0.9	1.66	-0.22	0.58
apv.sitar.s5	167	-0.04	0.38	0	-0.01	-3.21	0.62	3.83	-3.59	27.49
apv.sitar.s6	167	-0.04	0.3	-0.02	-0.04	-1.1	1.04	2.14	0.12	1.48
apv.sitar.s7	167	-0.02	0.37	-0.05	-0.04	-1.52	1.6	3.12	0.9	4.33
vpv.sitar.s1	167	0.33	1.14	0.38	0.3	-2.47	3.57	6.03	0.23	-0.35
vpv.sitar.s2	167	0.29	1.04	0.34	0.26	-2.28	3.22	5.5	0.24	-0.28
vpv.sitar.s3	167	0.24	1.01	0.24	0.22	-2.35	2.78	5.14	0.16	-0.46
vpv.sitar.s4	167	0.25	1.1	0.19	0.21	-2.51	4.11	6.61	0.44	0.48
vpv.sitar.s5	167	0.28	1.16	0.24	0.22	-2.57	3.92	6.49	0.43	0.17
vpv.sitar.s6	167	0.28	1.13	0.23	0.23	-2.49	3.65	6.15	0.33	-0.06
vpv.sitar.s7	167	0.26	1.11	0.26	0.22	-2.3	3.48	5.79	0.31	-0.22
ato.sitar.s1	167	0.04	0.64	0.06	0.05	-2.04	2.16	4.2	-0.15	1.21
ato.sitar.s2	167	0.13	0.53	0.09	0.12	-1.59	2.21	3.8	0.26	1.79
ato.sitar.s3	167	0.21	0.51	0.13	0.19	-1.01	2.21	3.22	0.67	1.37
ato.sitar.s4	167	0.21	0.55	0.17	0.18	-1.47	2.19	3.67	0.57	1.11
ato.sitar.s5	167	0.16	0.63	0.15	0.13	-2.57	2.17	4.74	0.06	2.12
ato.sitar.s6	167	0.15	0.6	0.11	0.12	-1.17	2.21	3.38	0.61	0.6
ato.sitar.s7	167	0.17	0.62	0.09	0.13	-1.21	2.25	3.46	0.63	0.46
vto.sitar.s1	167	0.3	0.42	0.27	0.31	-0.94	1.19	2.13	-0.17	-0.36
vto.sitar.s2	167	0.25	0.43	0.24	0.26	-0.99	1.14	2.13	-0.22	-0.25
vto.sitar.s3	167	0.22	0.44	0.21	0.23	-1.12	1.15	2.27	-0.26	-0.07
vto.sitar.s4	167	0.22	0.45	0.24	0.23	-1.15	1.41	2.56	-0.1	-0.21
vto.sitar.s5	167	0.24	0.46	0.29	0.25	-1.14	1.44	2.58	-0.17	-0.29
vto.sitar.s6	167	0.24	0.46	0.26	0.25	-1.11	1.4	2.51	-0.19	-0.3
vto.sitar.s7	167	0.23	0.46	0.26	0.24	-1.05	1.4	2.45	-0.2	-0.23

Table S9 Boys: Descriptive statistics of D values (differences between SITAR estimates and reference values) of all milestones for each testing sample (s1–s7) separately.

Table S10 Results of four Mixed Effects Linear Models analysis of differences (D) between estimates of growth milestones (APV, VPV, ATO, VTO) and their reference values.
The mixed-effects linear models were computed using the Maximum Likelihood estimator (ML) in the *R*-package *lme4* (Bates et al., 2015). Computation of p-values was based on conditional F-tests with the Kenward-Roger approximation for the degrees of freedom as available in the R-package *pbkrtest* (Halekoh and Hojsgaard 2014). Tables were formatted with the assistance of the *sjPlot* package (Lüdecke, 2021; Nakagawa et al., 2017) and *ggplot2* (Wickham, 2016).

Abbreviations in the MLM tables are as follows:

difference (D)—difference between estimate and the reference (observe) value
samp—individual subjects
met—consecutive observations (1–5)
sex—biological sex (male, female)
apv.ref—reference value of APV
vpv.ref—reference value of VPV
ato.ref—reference value of ATO
vto.ref—reference value of VTO
CI—95% confidence intervals
p—significance of the F-test
df—degrees of freedom
Random effects
σ2—random variance estimate
τ00—random intercept variance
τ11—random slope variance
ρ01—random slope-intercept correlation
ICC—intraclass correlation coefficient (proportion of variance explained by a grouping (random) factor - subjects)
N—number of groups (subjects)
R2—coefficient of determination

References

Bates D, Maechler M, Bolker B, Walker S (2015) Fitting linear mixed-effects models using lme4. J Statistical Software 67(1), 1–48. doi:10.18637/jss.v067.i01

Halekoh U, Hojsgaard S (2014) A Kenward-Roger approximation and parametric bootstrap methods for tests in linear mixed models: The R package pbkrtest. J Statistical Software 59(9), 1–30. URL http://www.jstatsoft.org/v59/i09/

Lüdecke D (2021) sjplot: Data visualization for statistics in social science. R package version 2.8.7. URL https://CRAN.R-project.org/package=sjPlot

Nakagawa S, Johnson P, Schielzeth H (2017) The coefficient of determination R^2 and intra-class correlation coefficient from generalized linear mixed-effects models revisited and expanded. J Roy Soc, Interface 14. doi:10.1098/rsif.2017.0213

Wickham H (2016) ggplot2: Elegant graphics for data analysis. Springer-Verlag, New York.

		difference		
Predictors	*Estimates*	*CI*	*p*	*df*
(Intercept)	5.34	4.44 – 6.23	**<0.001**	375.03
samp	–0.28	–0.45 – –0.12	**0.001**	398.07
met [SITAR]	0.86	0.42 – 1.30	**<0.001**	4000.00
sex [m]	–1.62	–2.99 – –0.25	**0.021**	375.03
apv.ref	–0.47	–0.55 – –0.39	**<0.001**	375.03
samp * met [SITAR]	–0.03	–0.13 – 0.07	0.521	4000.00
samp * sex [m]	0.31	0.05 – 0.56	**0.018**	398.07
met [SITAR] * sex [m]	–0.67	–1.36 – 0.01	0.053	4000.00
samp * apv.ref	0.03	0.01 – 0.04	**<0.001**	398.07
met [SITAR] * apv.ref	–0.07	–0.10 – –0.03	**0.001**	4000.00
sex [m] * apv.ref	0.18	0.07 – 0.29	**0.001**	375.03
(samp * met [SITAR]) *sex [m]	–0.09	–0.24 – 0.06	0.239	4000.00
(samp * met [SITAR]) *apv.ref	0.00	–0.01 – 0.01	0.785	4000.00
(samp * sex [m]) *apv.ref	–0.03	–0.05 – –0.01	**0.010**	398.07
(met [SITAR] * sex [m]) *apv.ref	0.06	0.01 – 0.12	**0.022**	4000.00
(samp * met [SITAR] * sex[m]) * apv.ref	0.01	–0.01 – 0.02	0.400	4000.00
**Random Effects**				
σ^2	0.04			
$\tau_{00\ ID}$	0.18			
$\tau_{11\ ID.samp}$	0.01			
$\rho_{01\ ID}$	–0.90			
ICC	0.64			
N $_{ID}$	334			
Observations	4676			
Marginal R^2 / Conditional R^2	0.504 / 0.820			

Predictors	difference<br>Estimates	CI	p	df
(Intercept)	3.62	2.83 – 4.41	**<0.001**	609.69
samp	0.22	0.05 – 0.39	**0.010**	662.84
met [SITAR]	2.62	1.81 – 3.43	**<0.001**	4000.00
sex [m]	–0.25	–1.30 – 0.80	0.638	609.69
vpv.ref	–0.43	–0.53 – –0.32	**<0.001**	609.69
samp * met [SITAR]	–0.20	–0.39 – –0.02	**0.029**	4000.00
samp * sex [m]	0.08	–0.14 – 0.30	0.483	662.84
met [SITAR] * sex [m]	1.53	0.45 – 2.61	**0.006**	4000.00
samp * vpv.ref	–0.04	–0.06 – –0.01	**0.001**	662.84
met [SITAR] * vpv.ref	-0.35	–0.45 – –0.24	**<0.001**	4000.00
sex [m] * vpv.ref	0.14	0.02 – 0.27	**0.028**	609.69
(samp * met [SITAR]) *sex [m]	–0.02	–0.27 – 0.22	0.841	4000.00
(samp * met [SITAR]) *vpv.ref	0.03	0.00 – 0.05	**0.020**	4000.00
(samp * sex [m]) * vpv.ref	–0.01	–0.03 – 0.02	0.603	662.84
(met [SITAR] * sex [m]) *vpv.ref	–0.15	–0.28 – –0.02	**0.023**	4000.00
(samp * met [SITAR] * sex[m]) * vpv.ref	0.01	–0.02 – 0.04	0.688	4000.00
**Random Effects**				
σ^2	0.27			
$\tau_{00\ ID}$	0.16			
$\tau_{11\ ID.samp}$	0.01			
$\rho_{01\ ID}$	–0.97			
ICC	0.13			
N_{ID}	334			
Observations	4676			
Marginal R^2 / Conditional R^2	0.634 / 0.682			

Predictors	Estimates	difference CI	*p*	*df*
(Intercept)	5.42	4.92 – 5.92	**<0.001**	411.10
samp	0.14	0.04 – 0.24	**0.006**	436.03
met [SITAR]	0.85	0.53 – 1.17	**<0.001**	4000.00
sex [m]	–0.55	–1.33 – 0.23	0.168	411.10
ato.ref	–0.57	–0.63 – –0.52	**<0.001**	411.10
samp * met [SITAR]	–0.23	–0.30 – –0.16	**<0.001**	4000.00
samp * sex [m]	–0.04	–0.20 – 0.11	0.596	436.03
met [SITAR] * sex [m]	–0.58	–1.09 – –0.08	**0.023**	4000.00
samp * ato.ref	–0.01	–0.02 – 0.00	0.087	436.03
met [SITAR] * ato.ref	–0.09	–0.13 – –0.06	**<0.001**	4000.00
sex [m] * ato.ref	0.13	0.05 – 0.21	**0.001**	411.10
(samp * met [SITAR]) *sex [m]	0.21	0.09 – 0.32	**<0.001**	4000.00
(samp * met [SITAR]) *ato.ref	0.02	0.02 – 0.03	**<0.001**	4000.00
(samp * sex [m]) *ato.ref	0.01	–0.01 – 0.02	0.506	436.03
(met [SITAR] * sex [m]) *ato.ref	0.05	0.00 – 0.11	**0.038**	4000.00
(samp * met [SITAR] * sex[m]) * ato.ref	–0.03	–0.04 – –0.02	**<0.001**	4000.00
**Random Effects**				
σ^2	0.03			
$\tau_{00\ ID}$	0.09			
$\tau_{11\ ID.samp}$	0.00			
$\rho_{01\ ID}$	–0.45			
ICC	0.74			
N_{ID}	334			
Observations	4676			
Marginal R^2 / Conditional R^2	0.689 / 0.919			

Predictors	difference Estimates	CI	p	df
(Intercept)	0.19	–0.13 – 0.50	0.240	432.72
samp	0.49	0.45 – 0.54	**<0.001**	635.98
met [SITAR]	3.23	3.00 – 3.45	**<0.001**	4000.00
sex [m]	0.97	0.50 – 1.43	**<0.001**	432.72
vto.ref	0.01	–0.05 – 0.07	0.791	432.72
samp * met [SITAR]	–0.43	–0.48 – –0.38	**<0.001**	4000.00
samp * sex [m]	–0.21	–0.28 – –0.14	**<0.001**	635.98
met [SITAR] * sex [m]	–1.11	–1.44 – –0.77	**<0.001**	4000.00
samp * vto.ref	–0.10	–0.11 – –0.09	**<0.001**	635.98
met [SITAR] * vto.ref	–0.61	–0.65 – –0.57	**<0.001**	4000.00
sex [m] * vto.ref	–0.21	–0.31 – –0.12	**<0.001**	432.72
(samp * met [SITAR]) *sex [m]	0.21	0.14 – 0.29	**<0.001**	4000.00
(samp * met [SITAR]) *vto.ref	0.09	0.08 – 0.10	**<0.001**	4000.00
(samp * sex [m]) *vto.ref	0.04	0.02 – 0.05	**<0.001**	635.98
(met [SITAR] * sex [m]) *vto.ref	0.19	0.12 – 0.25	**<0.001**	4000.00
(samp * met [SITAR] * sex[m]) * vto.ref	–0.04	–0.05 – –0.02	**<0.001**	4000.00
**Random Effects**				
σ^2	0.02			
$\tau_{00\ ID}$	0.05			
$\tau_{11\ ID.samp}$	0.00			
$\rho_{01\ ID}$	–0.33			
ICC	0.67			
N_{ID}	334			
Observations	4676			
Marginal R^2 / Conditional R^2	0.644 / 0.881			

	numDF	denDF	F-value	p-value
(Intercept)	1	4330	633.755	**<0.0001**
samp	1	4330	70.842	**<0.0001**
met	1	4330	5.469	**0.02**
sex	1	330	33.383	**<0.0001**
vpv.ref	1	330	5345.838	**<0.0001**
samp:met	1	4330	42.93	**<0.0001**
samp:sex	1	4330	0.312	0.6
met:sex	1	4330	19.939	**<0.0001**
samp:vpv.ref	1	4330	20.095	**<0.0001**
met:vpv.ref	1	4330	499.773	**<0.0001**
sex:vpv.ref	1	330	6.136	**0.014**
samp:met:sex	1	4330	25.96	**<0.0001**
samp:met:vpv.ref	1	4330	20.479	**<0.0001**
samp:sex:vpv.ref	1	4330	0.13	0.7
met:sex:vpv.ref	1	4330	18.186	**<0.0001**
samp:met:sex:vpv.ref	1	4330	0.161	0.7

Table S11 Analysis of Variance of the Linear Mixed Effects model for differences (D) between estimates and references values of Peak Velocity (VPV) with effects of sample (*samp*, 1–7), sex (*sex*, males, females), estimation method (*met*, FPCA, SITAR), and reference VPV (*vpv.ref*, in cm per year), including all interactions.

	numDF	denDF	F-value	p-value
(Intercept)	1	4330	199.064	**<0.0001**
samp	1	4330	151.1493	**<0.0001**
met	1	4330	1018.19	**<0.0001**
sex	1	330	0.002	0.96
vto.ref	1	330	701.3529	**<0.0001**
samp:met	1	4330	52.1274	**<0.0001**
samp:sex	1	4330	7.3955	**0.007**
met:sex	1	4330	31.1382	**<0.0001**
samp:vto.ref	1	4330	297.9201	**<0.0001**
met:vto.ref	1	4330	1084.619	**<0.0001**
sex:vto.ref	1	330	2.2175	0.14
samp:met:sex	1	4330	0.1006	0.75
samp:met:vto.ref	1	4330	360.9978	**<0.0001**
samp:sex:vto.ref	1	4330	9.2265	**0.002**
met:sex:vto.ref	1	4330	7.868	**0.0051**
samp:met:sex:vto.ref	1	4330	22.6918	**<0.0001**

Table S12 Analysis of Variance of the Linear Mixed Effects model for differences (D) between estimates and references values of Velocity at Take-off (VTO) with effects of sample (*samp*, 1–7), sex (*sex*, males, females), estimation method (*met*, FPCA, SITAR), and reference VTO (*vto.ref*, in cm per year), including all interactions.
