# Supplementary figures and images for: Estimating Growth in Height from Limited Longitudinal Growth Data Using Full-Curves Training Dataset: A Comparison of Two Procedures of Curve Optimization—Functional Principal Component Analysis and SITAR
Source: Children (Basel). 2021 Oct 18;8(10):934. doi: 10.3390/children8100934 (PMC8535004; doi:10.3390/children8100934)

Body height – Brno Growth Study – Boys

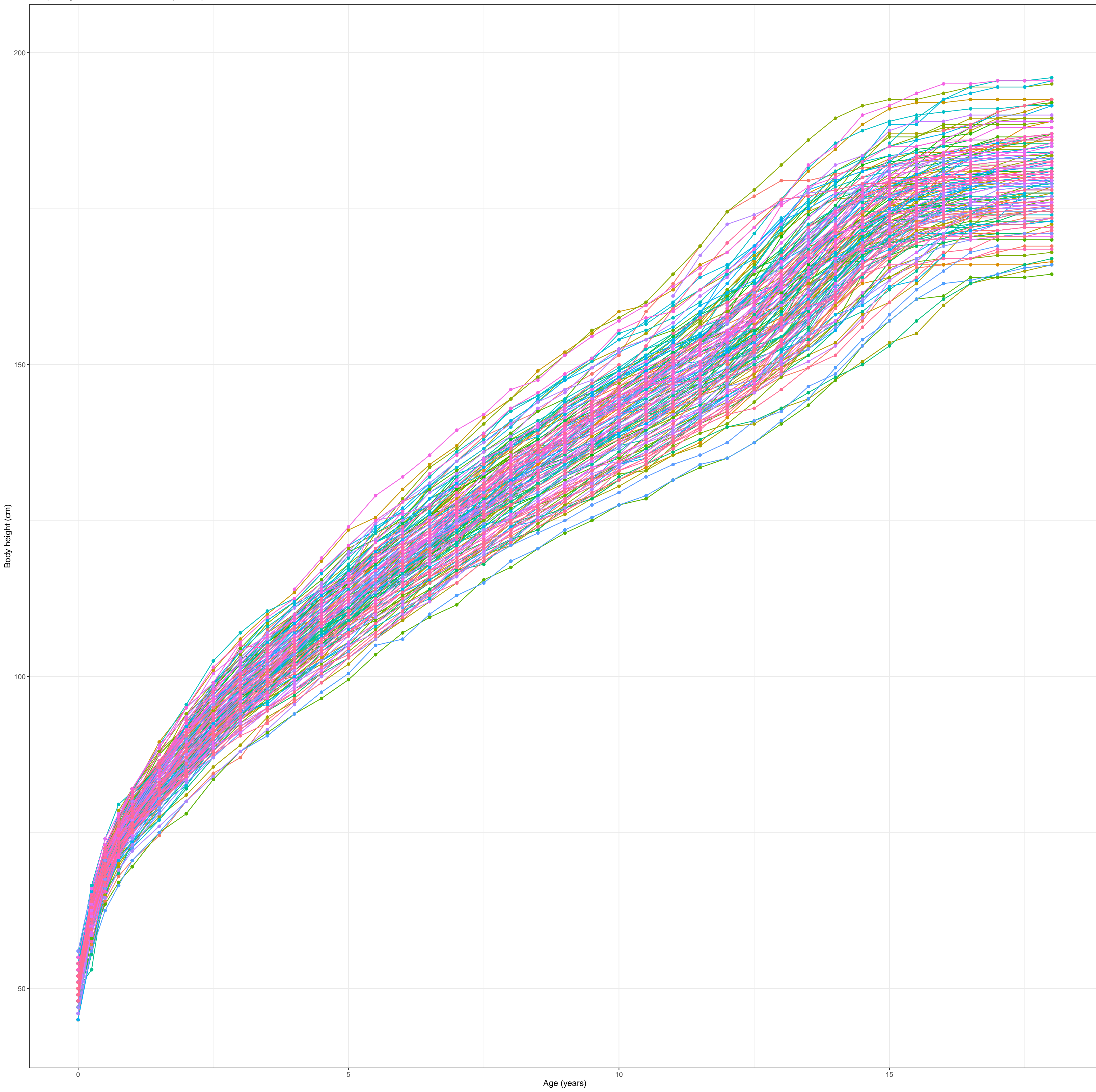

Supplement: Supplementary file 1 [file children-08-00934-s001.zip › Suplementary_materials/Figure_S01_Detailed_raw_data_boys.pdf]

Body height – Brno Growth Study – Girls

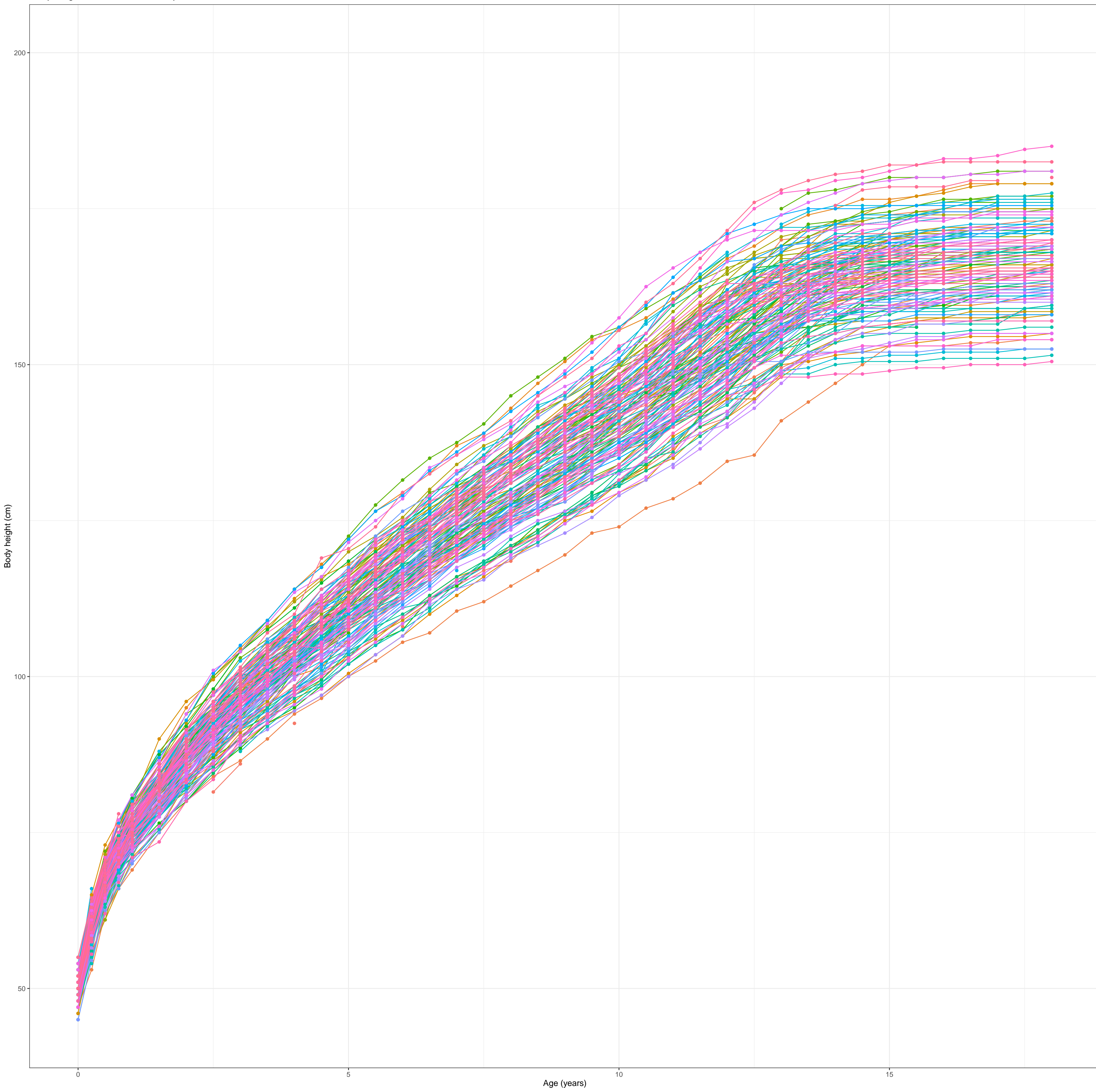

Supplement: Supplementary file 1 [file children-08-00934-s001.zip › Suplementary_materials/Figure_S02_Detailed_raw_data_girls.pdf]

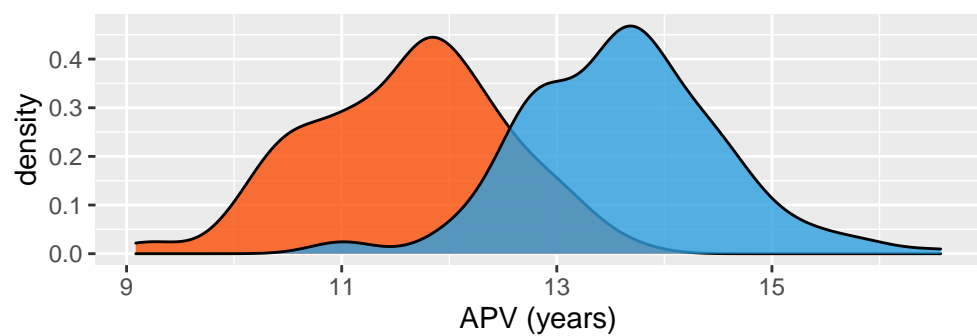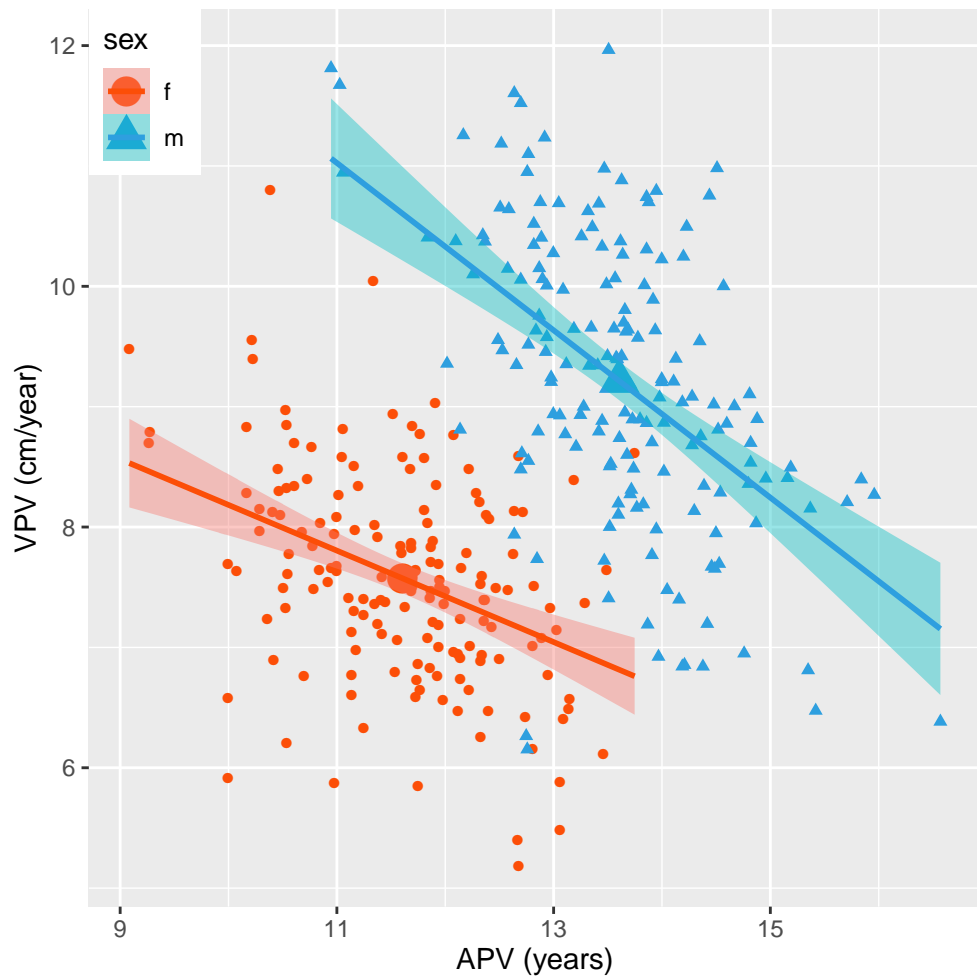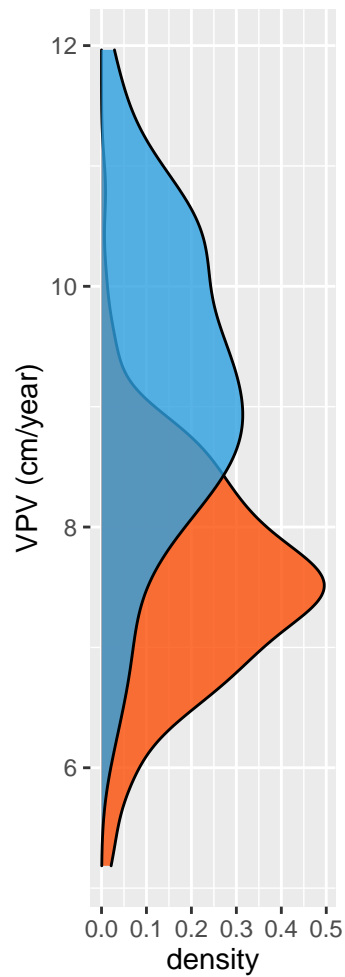

Supplement: Supplementary file 1 [file children-08-00934-s001.zip › Suplementary_materials/Figure_S04_Descriptive_plots_reference_VPV_to_APV.pdf]

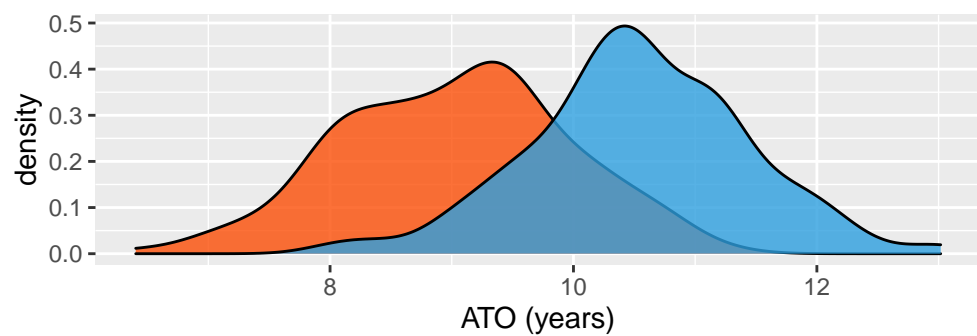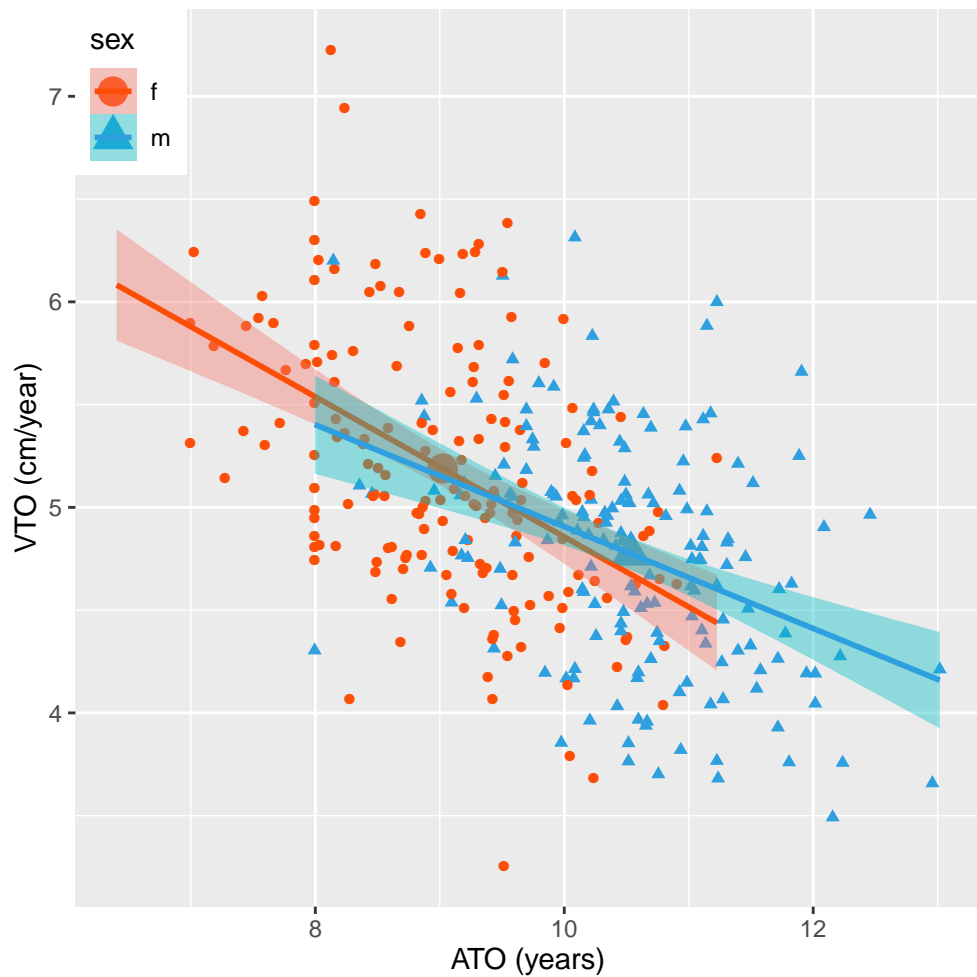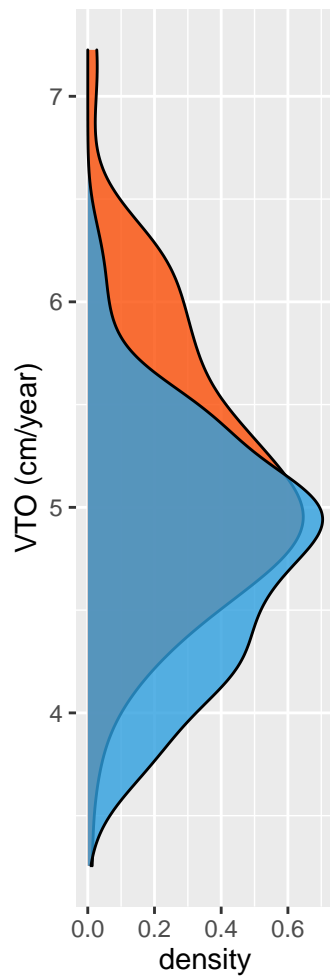

Supplement: Supplementary file 1 [file children-08-00934-s001.zip › Suplementary_materials/Figure_S05_Descriptive_plots_reference_VTO_to_ATO.pdf]

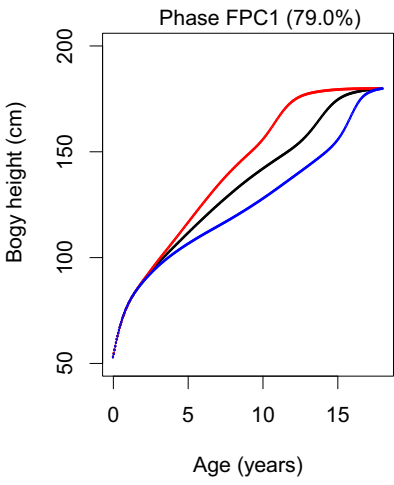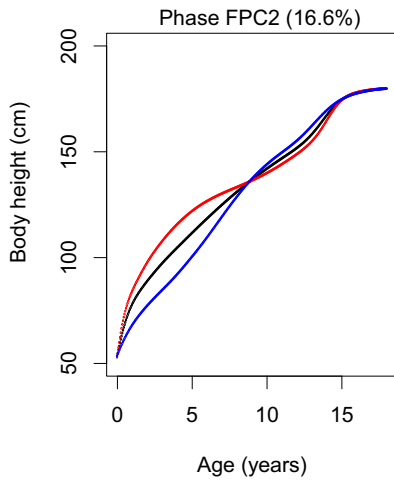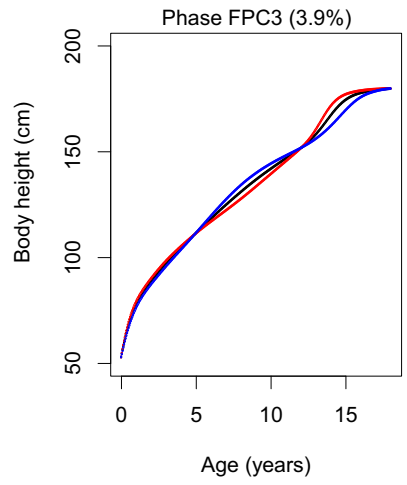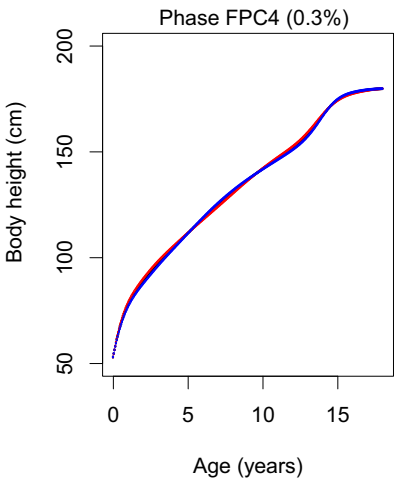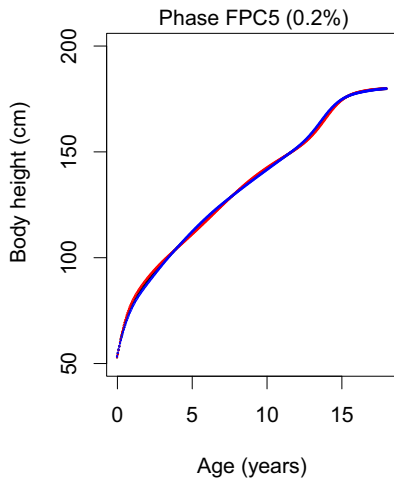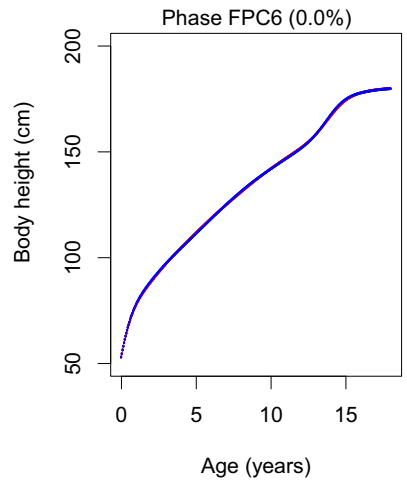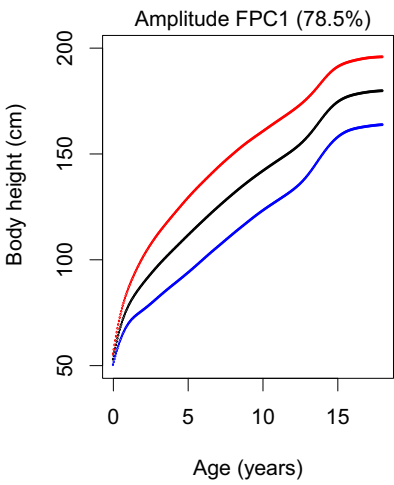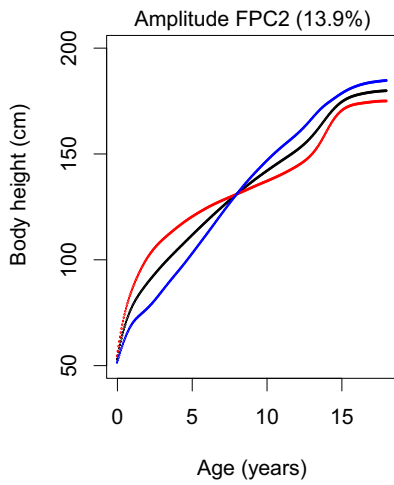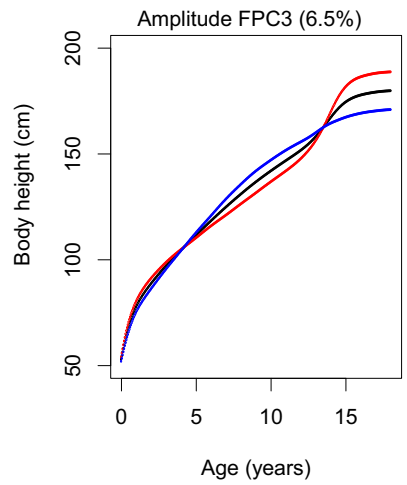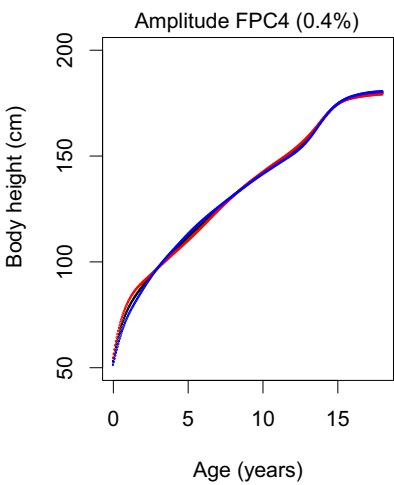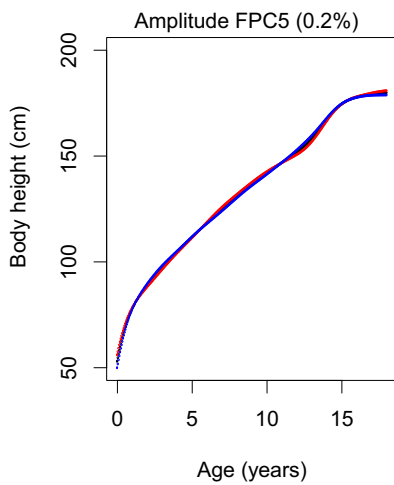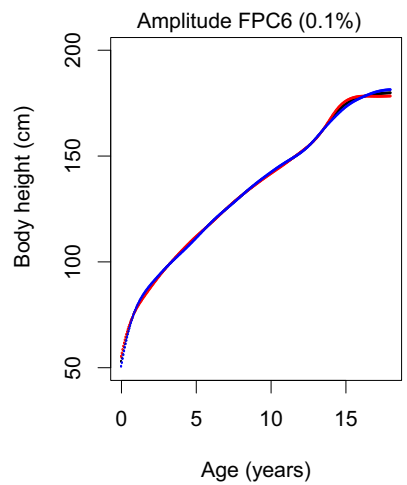

Supplement: Supplementary file 1 [file children-08-00934-s001.zip › Suplementary_materials/Figure_S06_FPCS_all12PCs_BOYS.pdf]

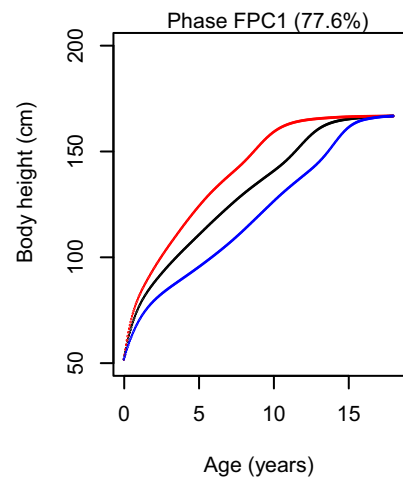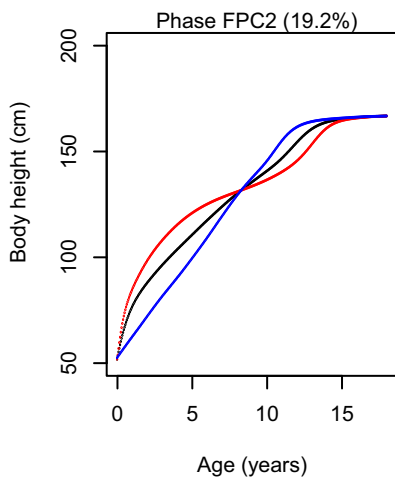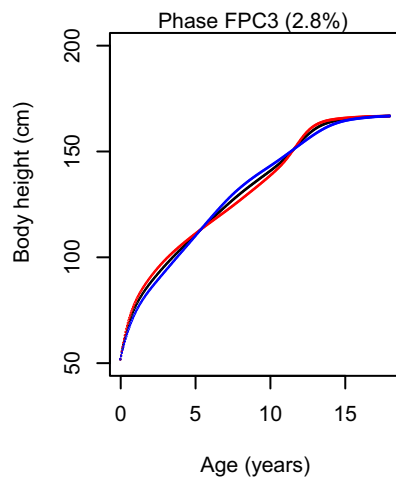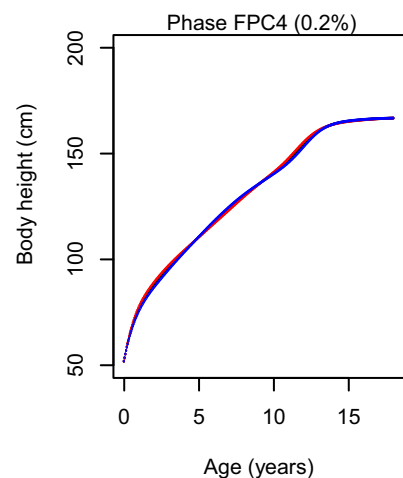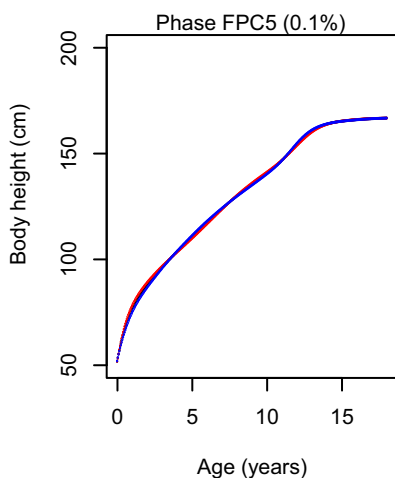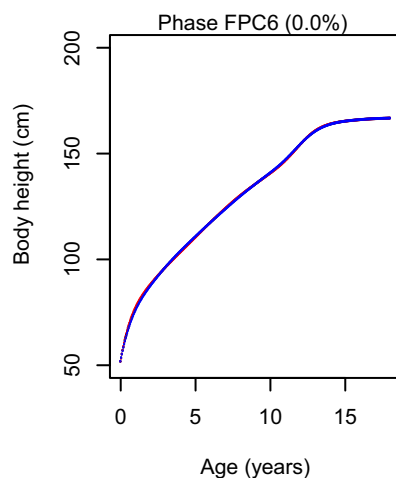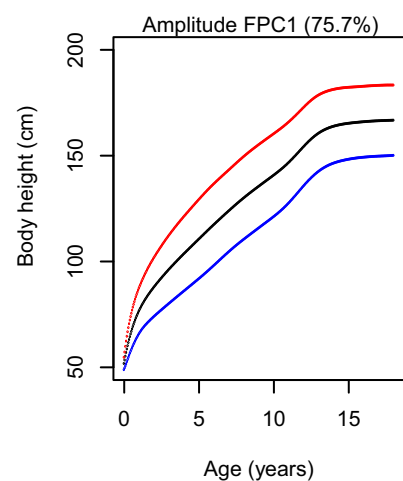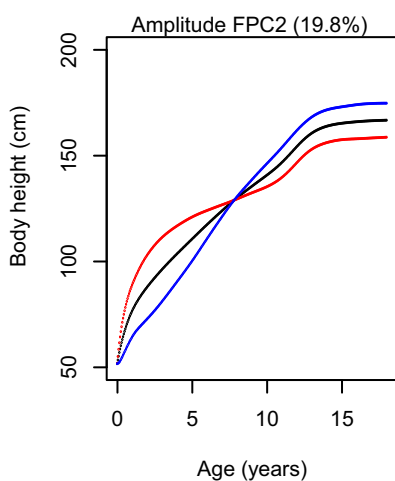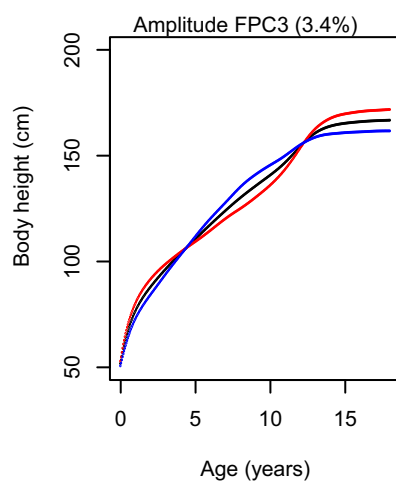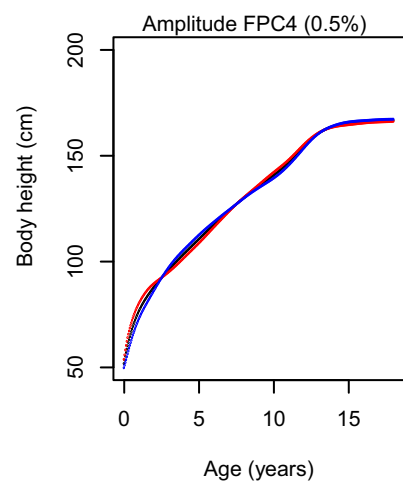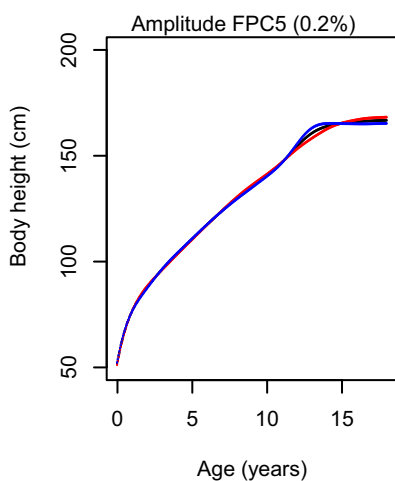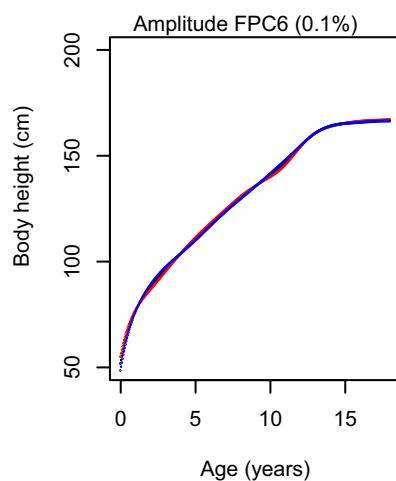

Supplement: Supplementary file 1 [file children-08-00934-s001.zip › Suplementary_materials/Figure_S07_FPCS_all12PCs_GIRLS.pdf]

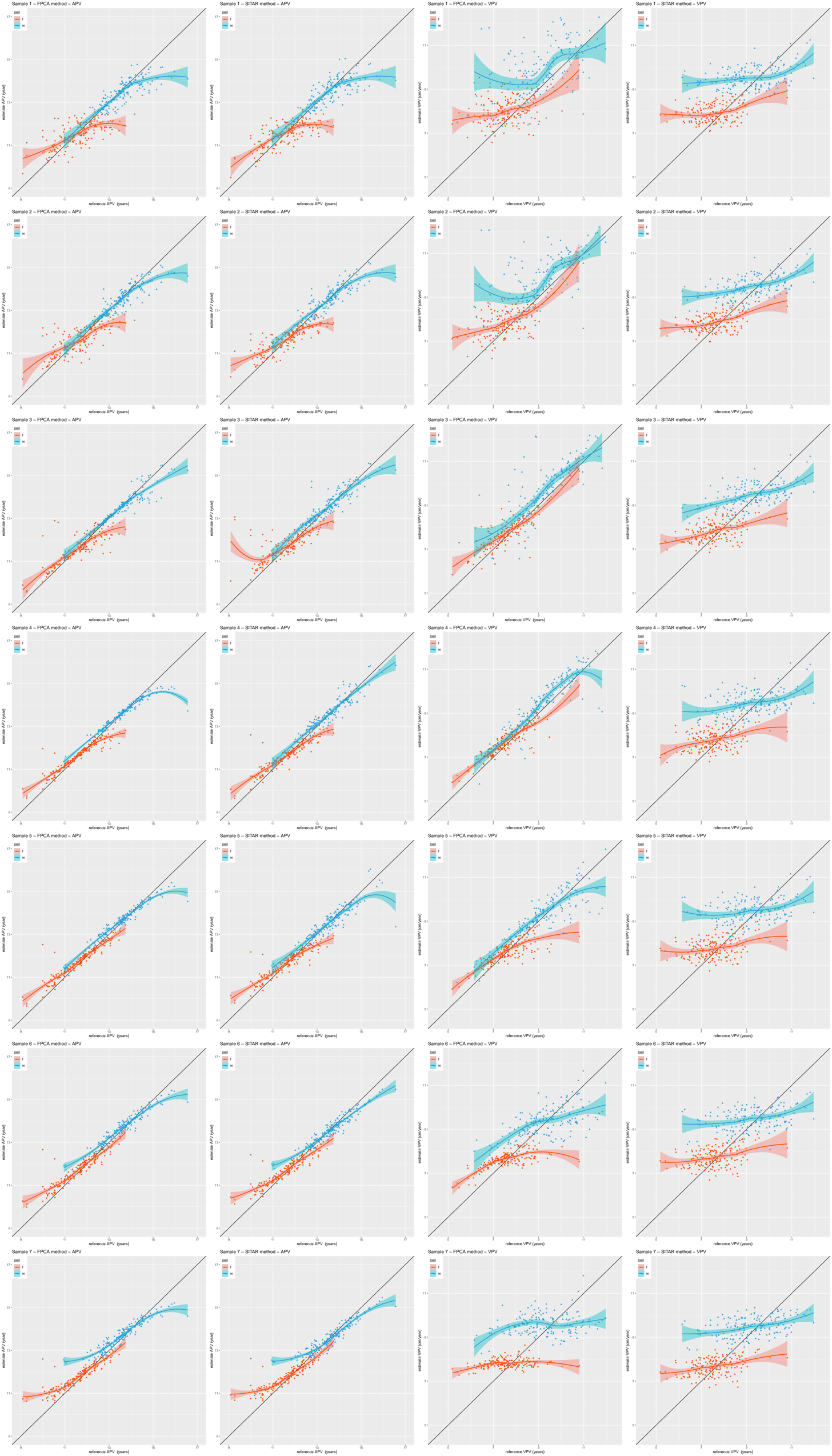

Supplement: Supplementary file 1 [file children-08-00934-s001.zip › Suplementary_materials/Figure_S08_APV_Estimates_vs_reference.pdf]

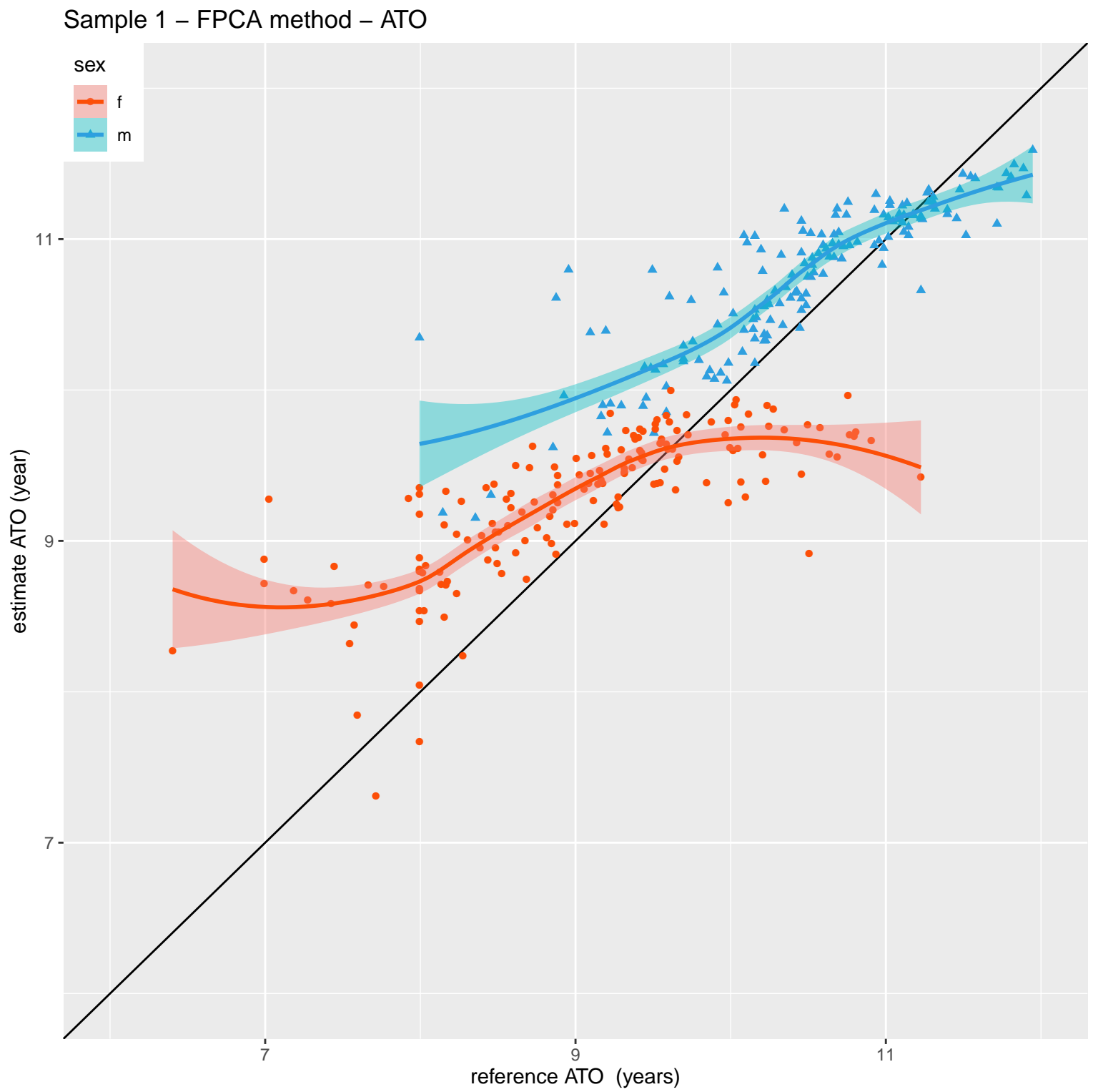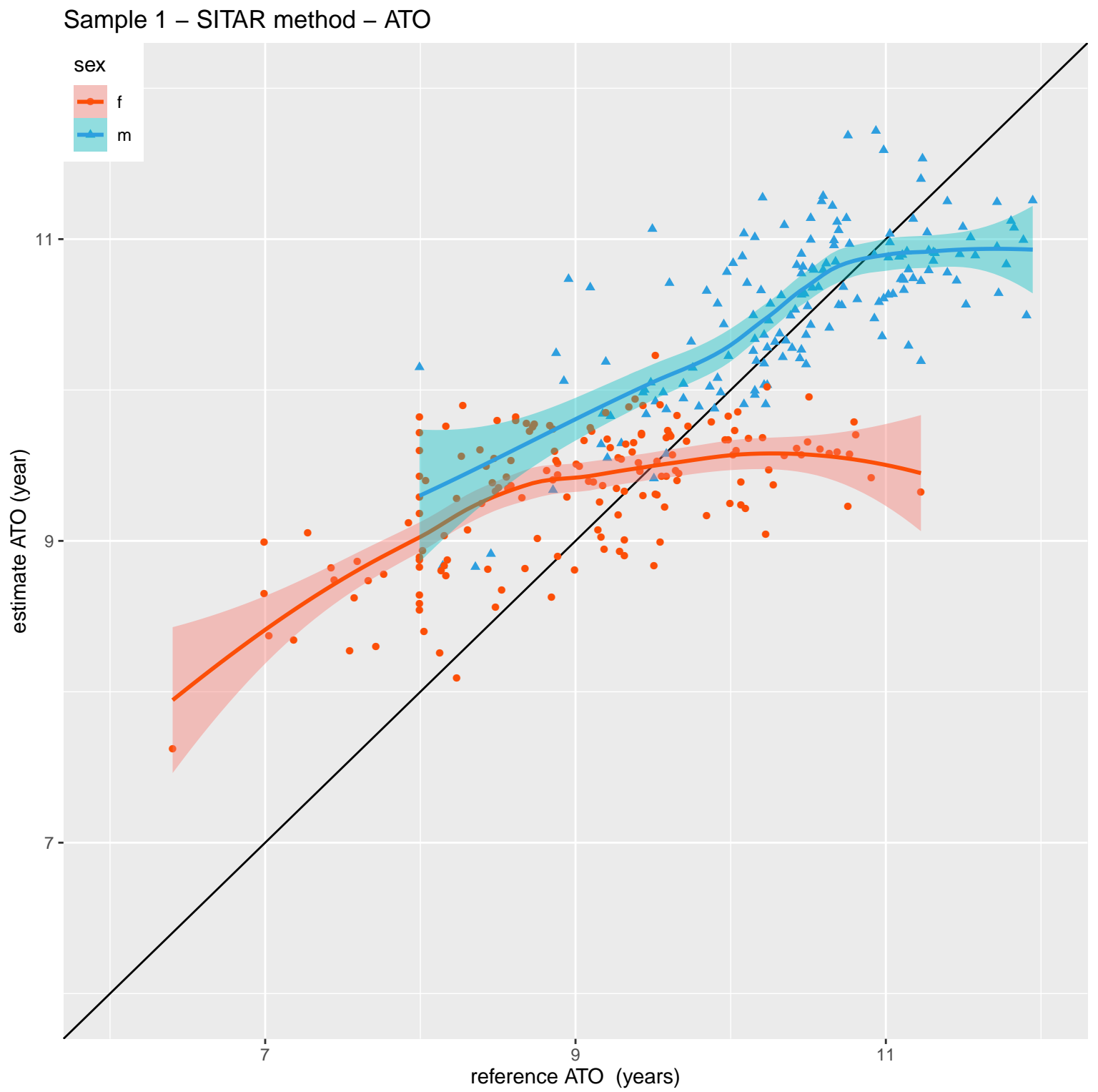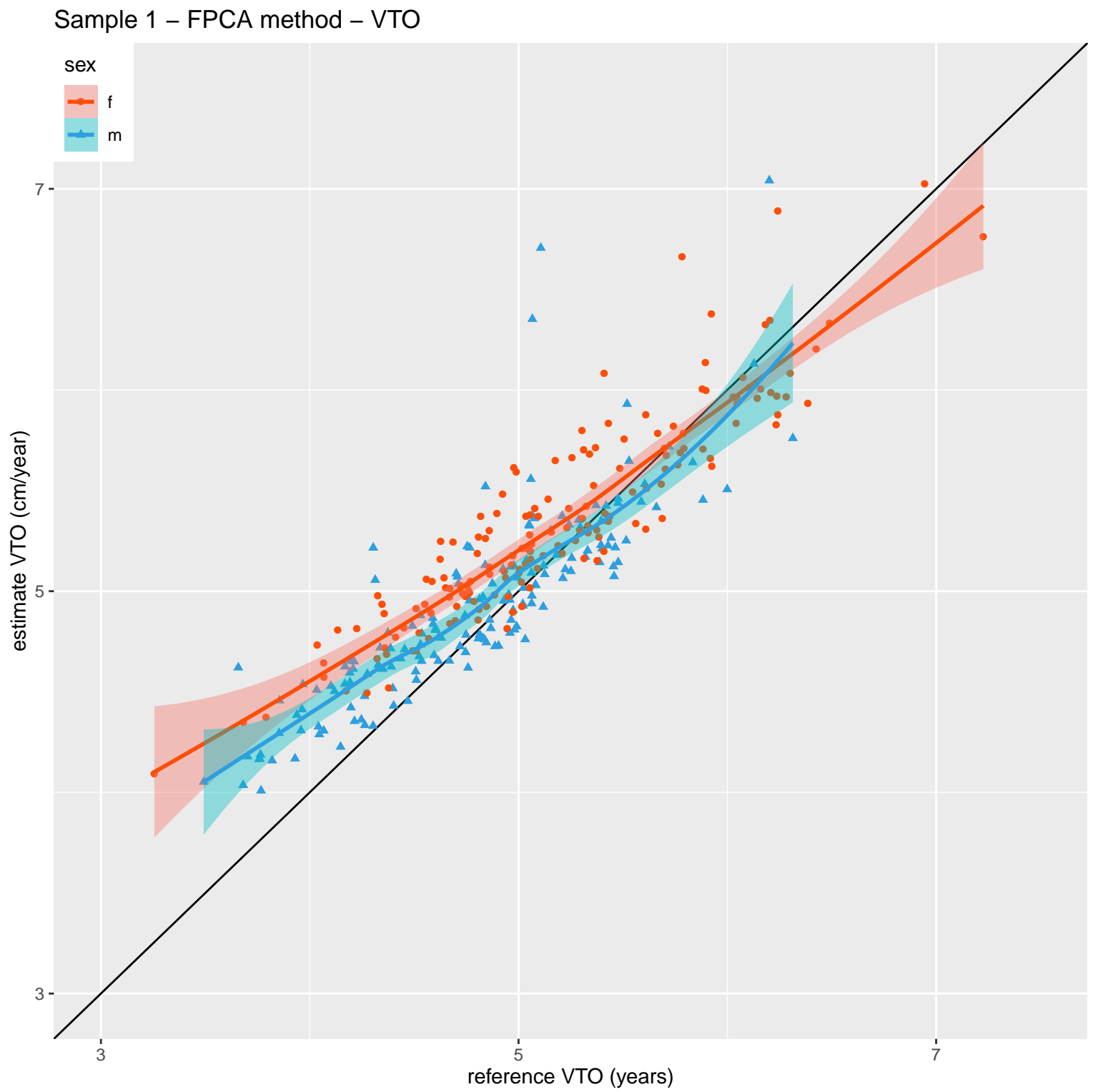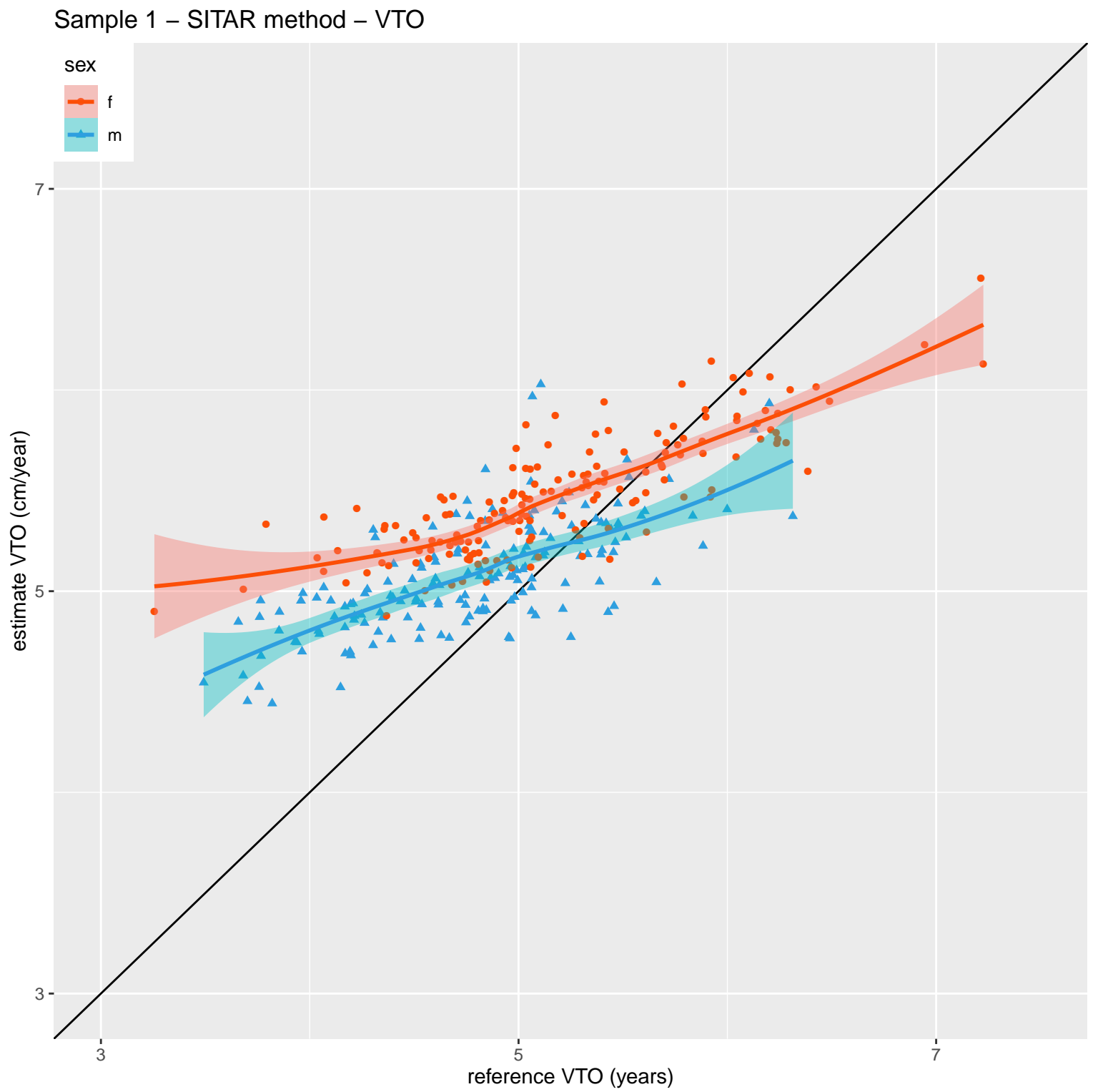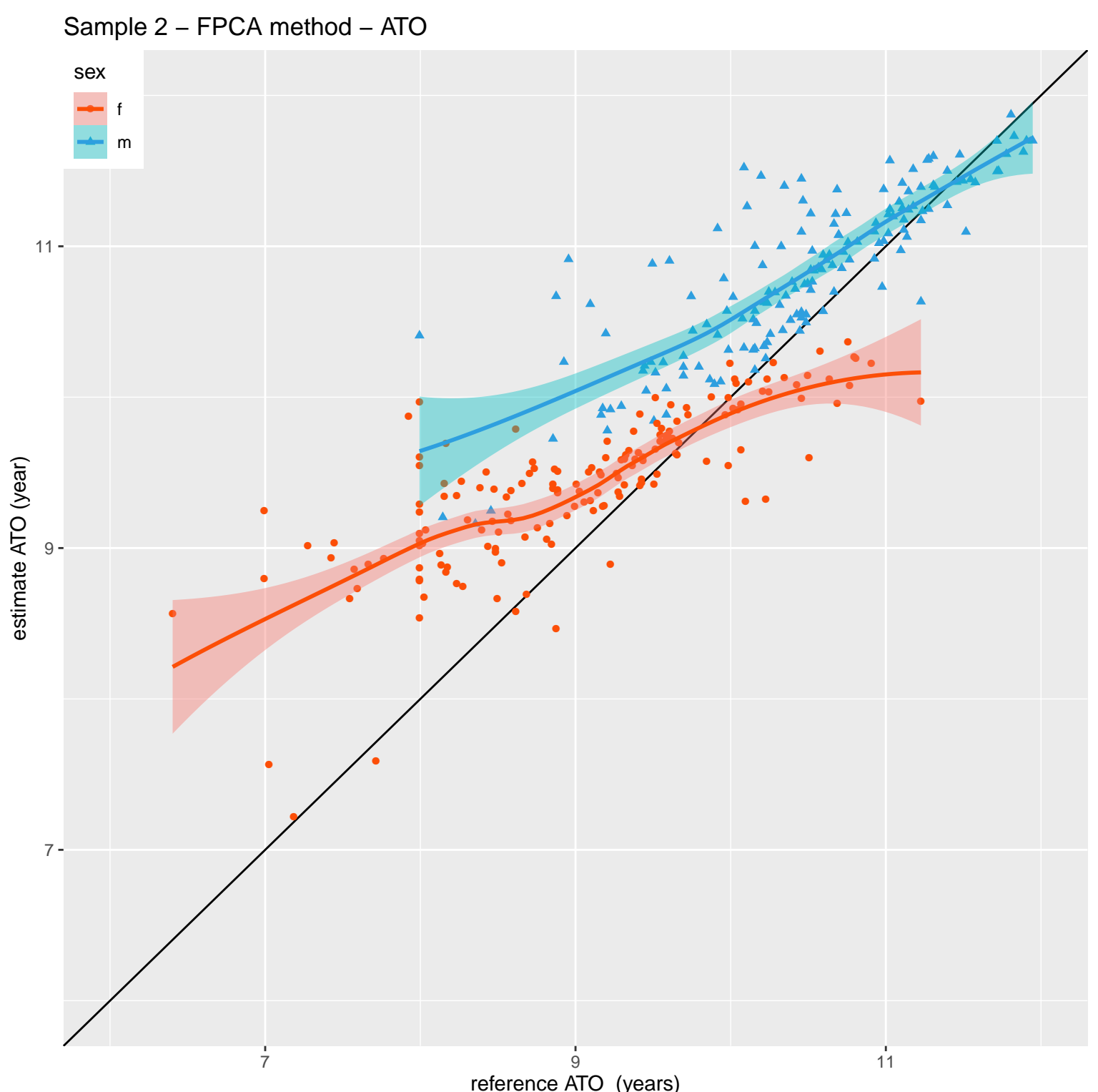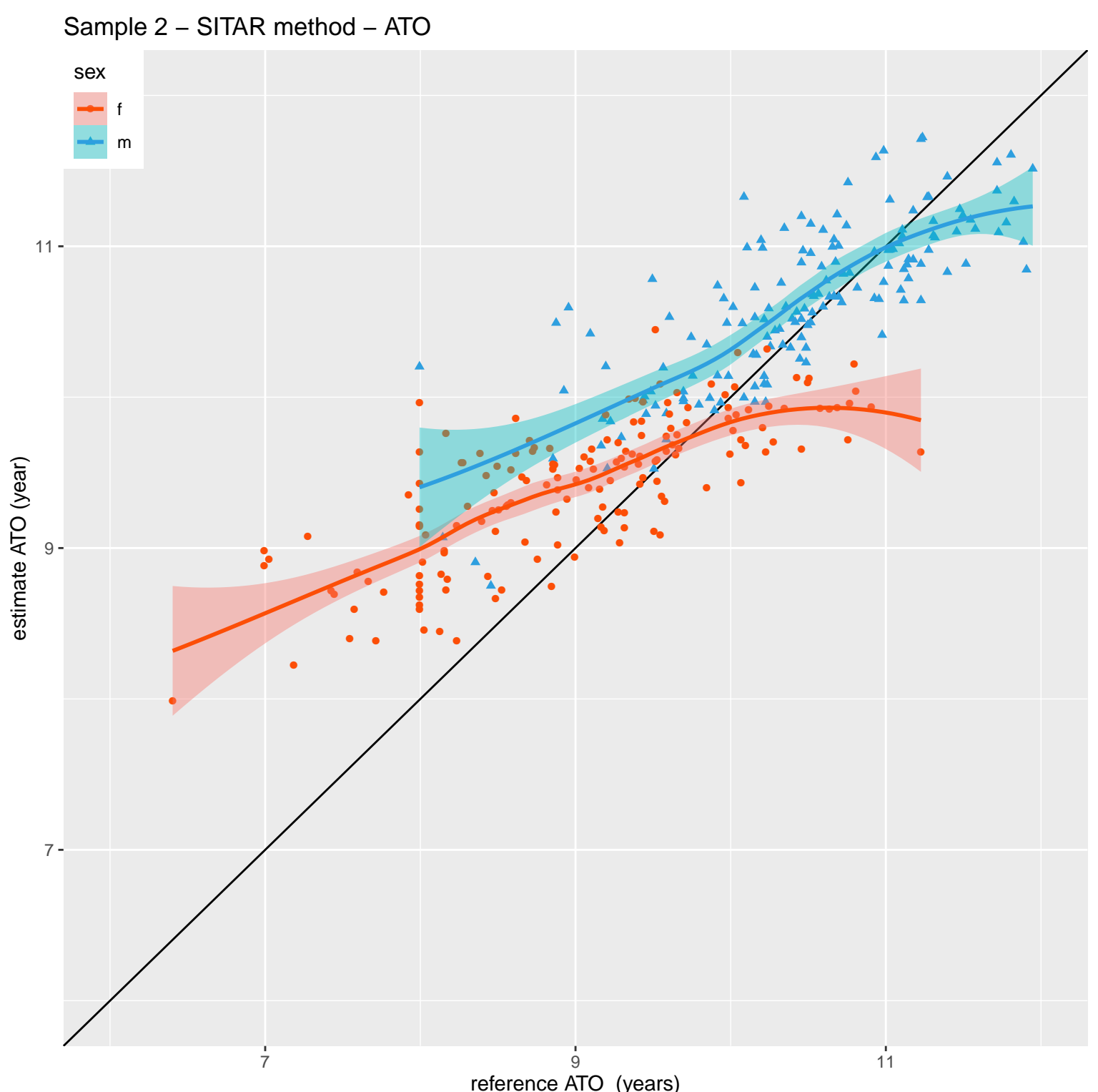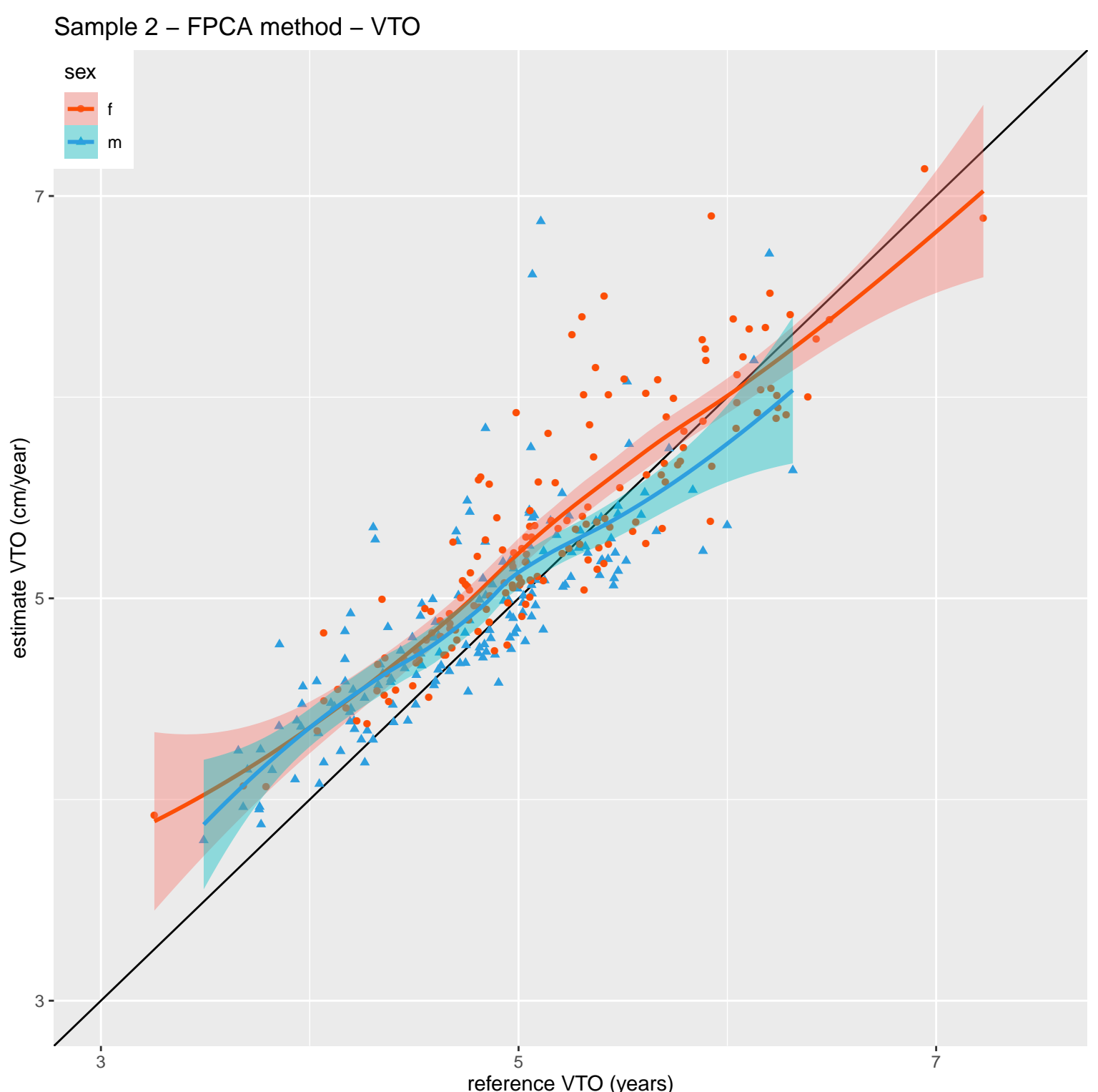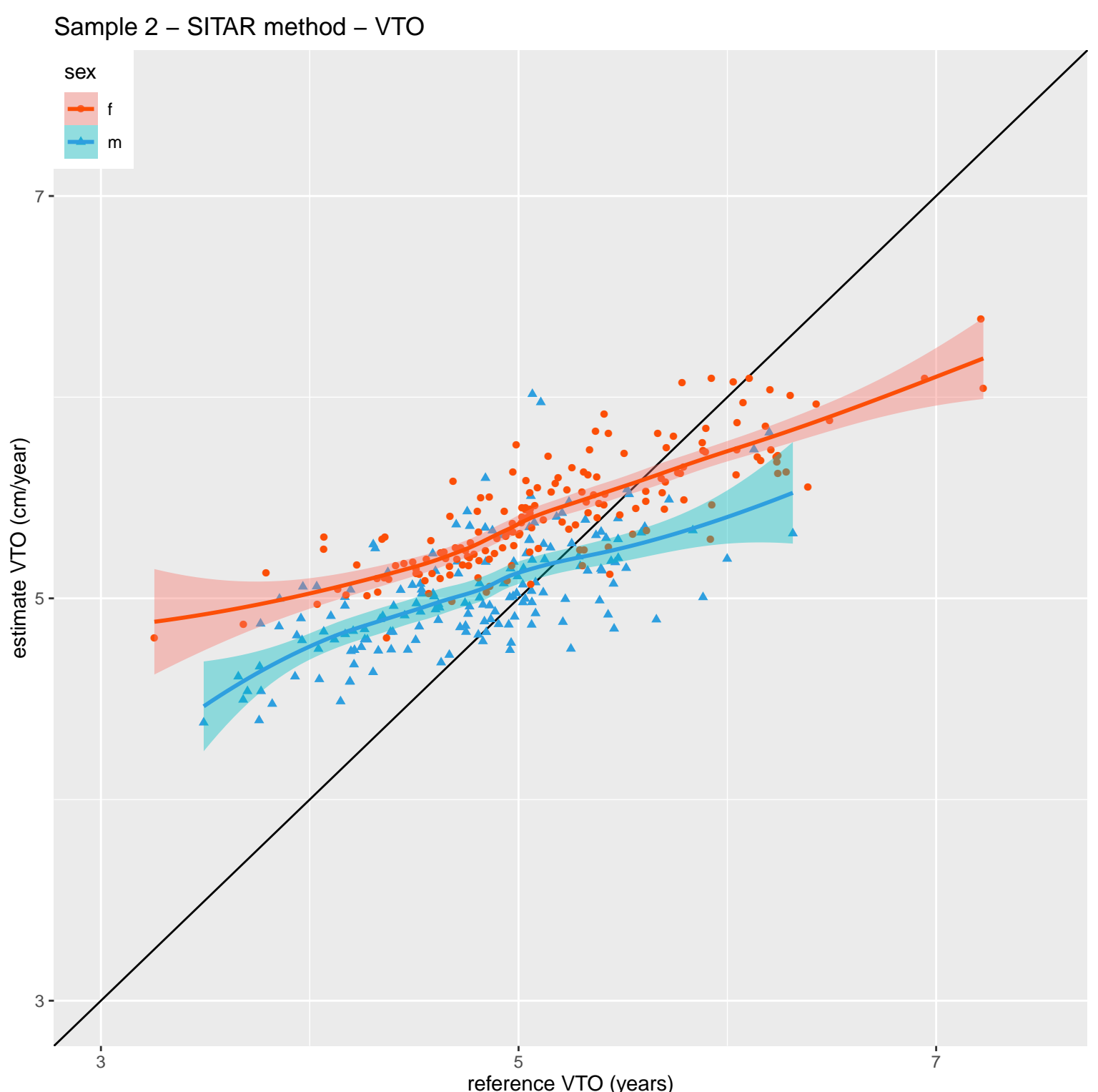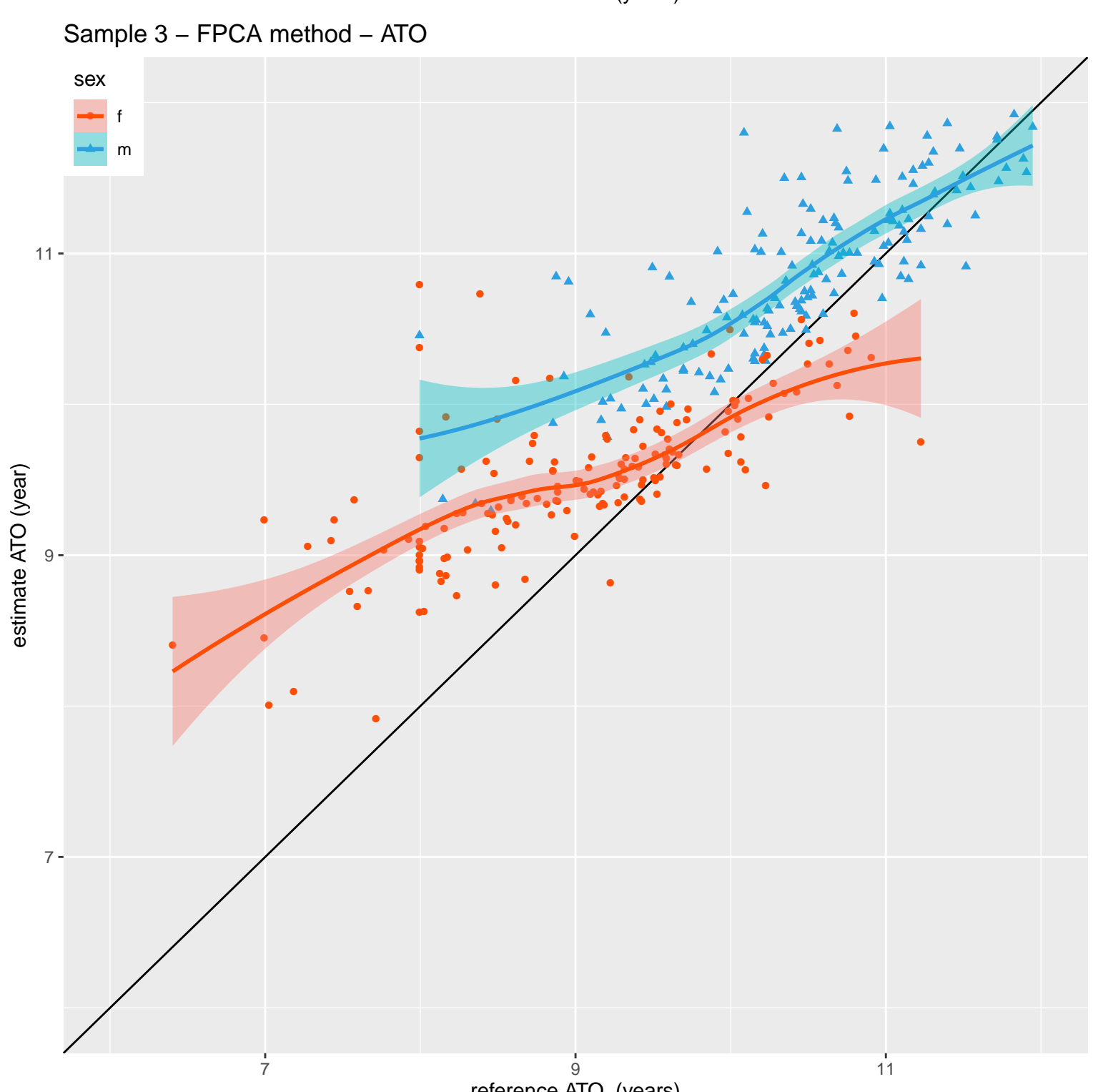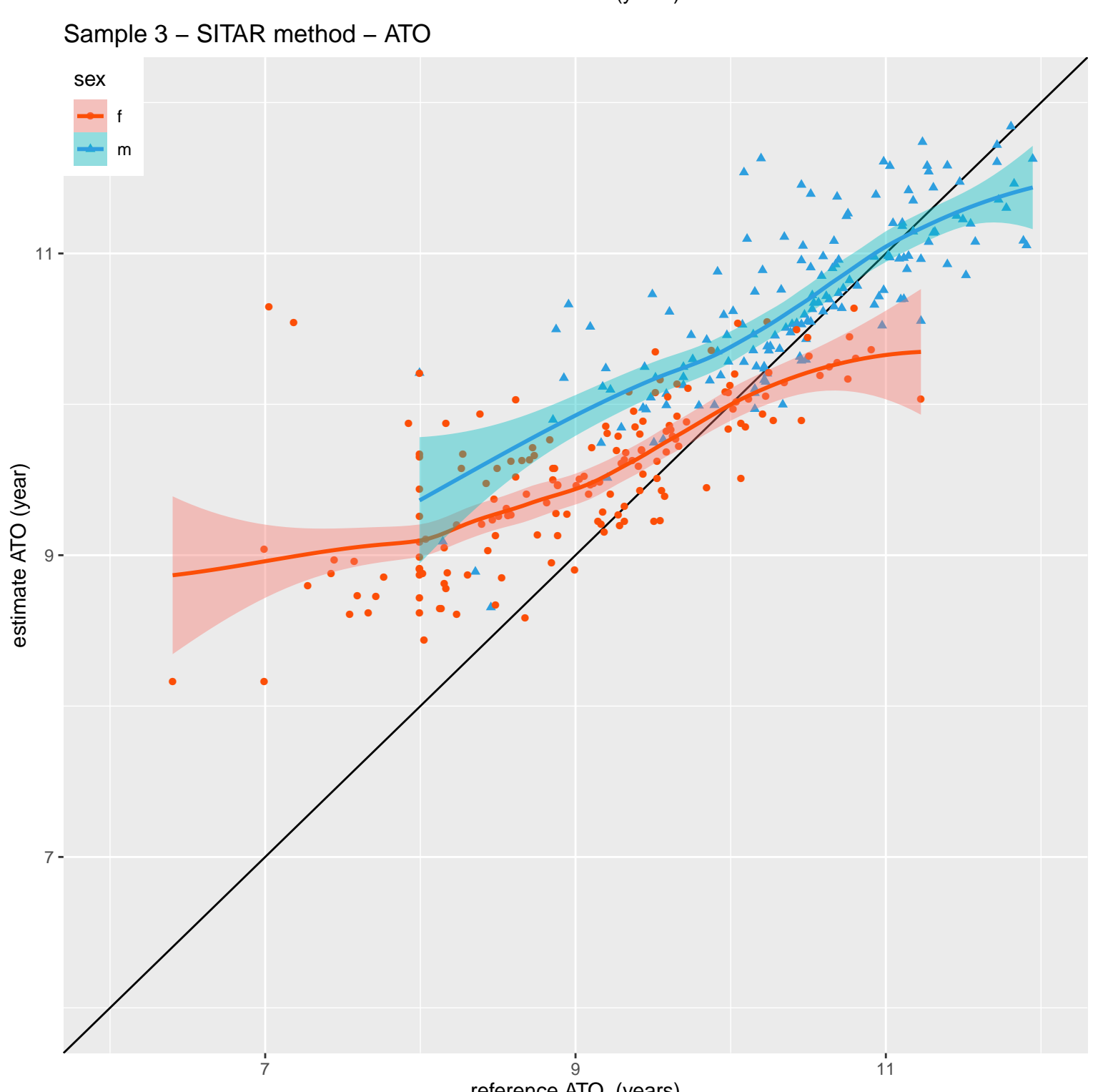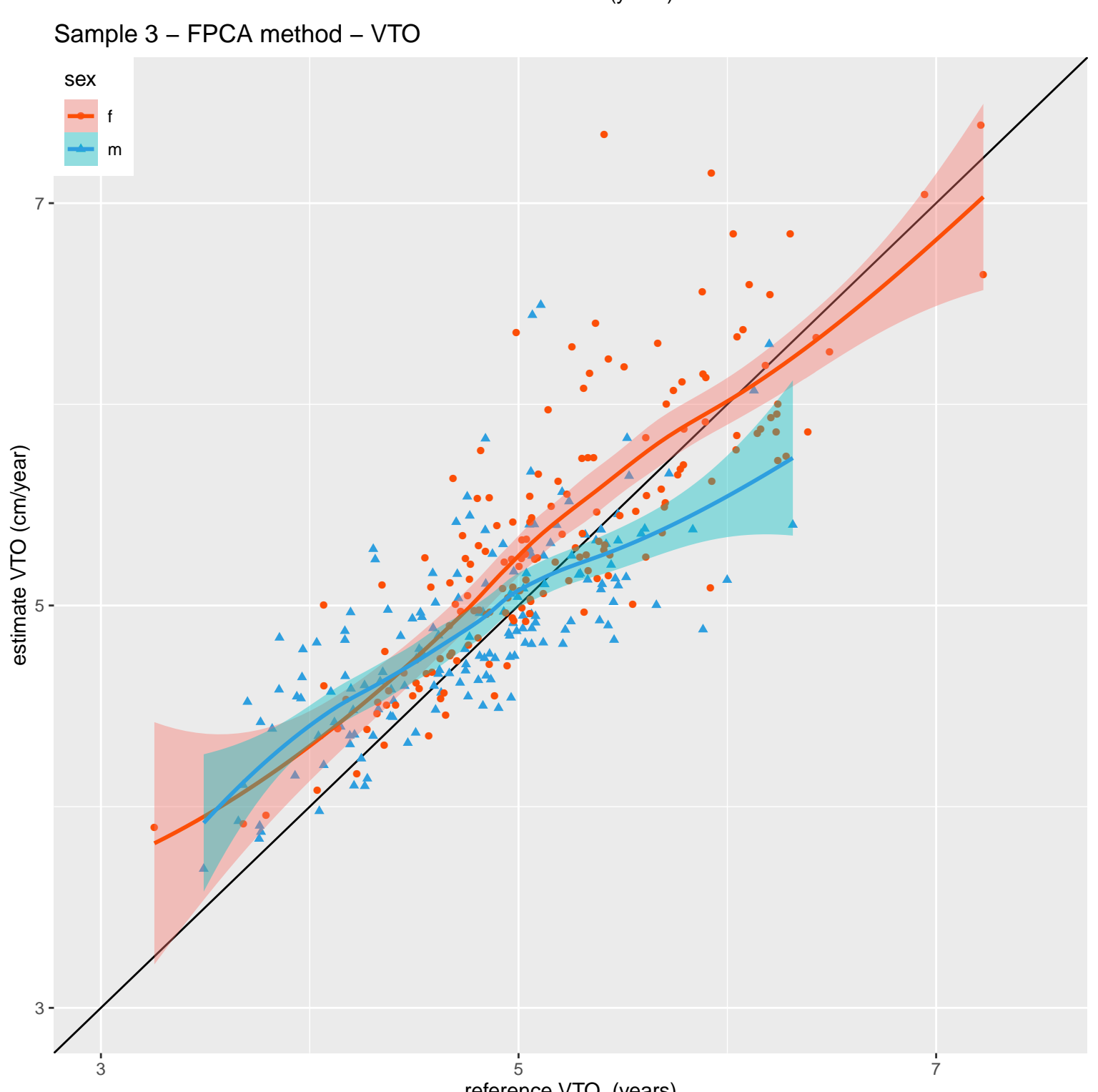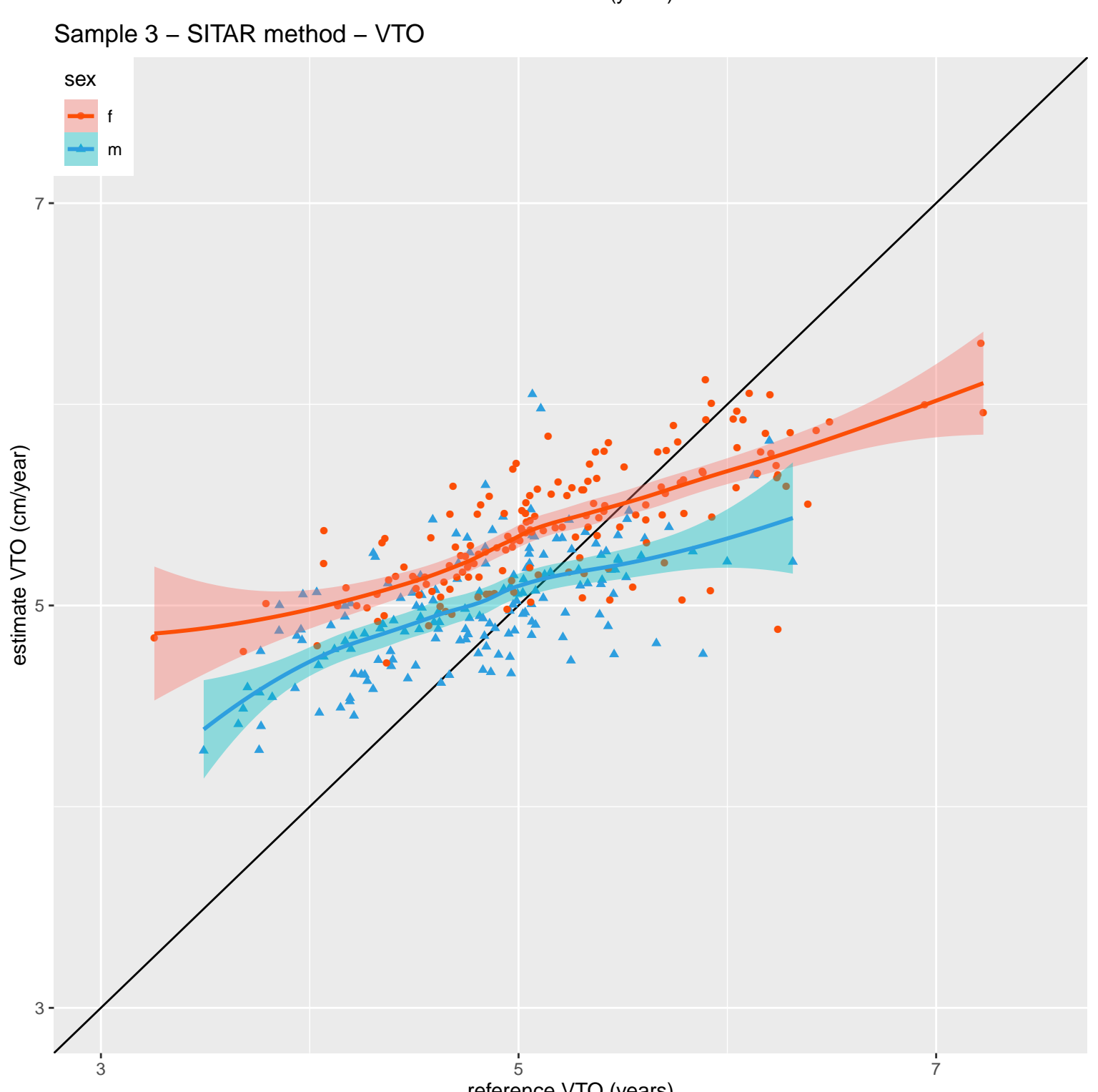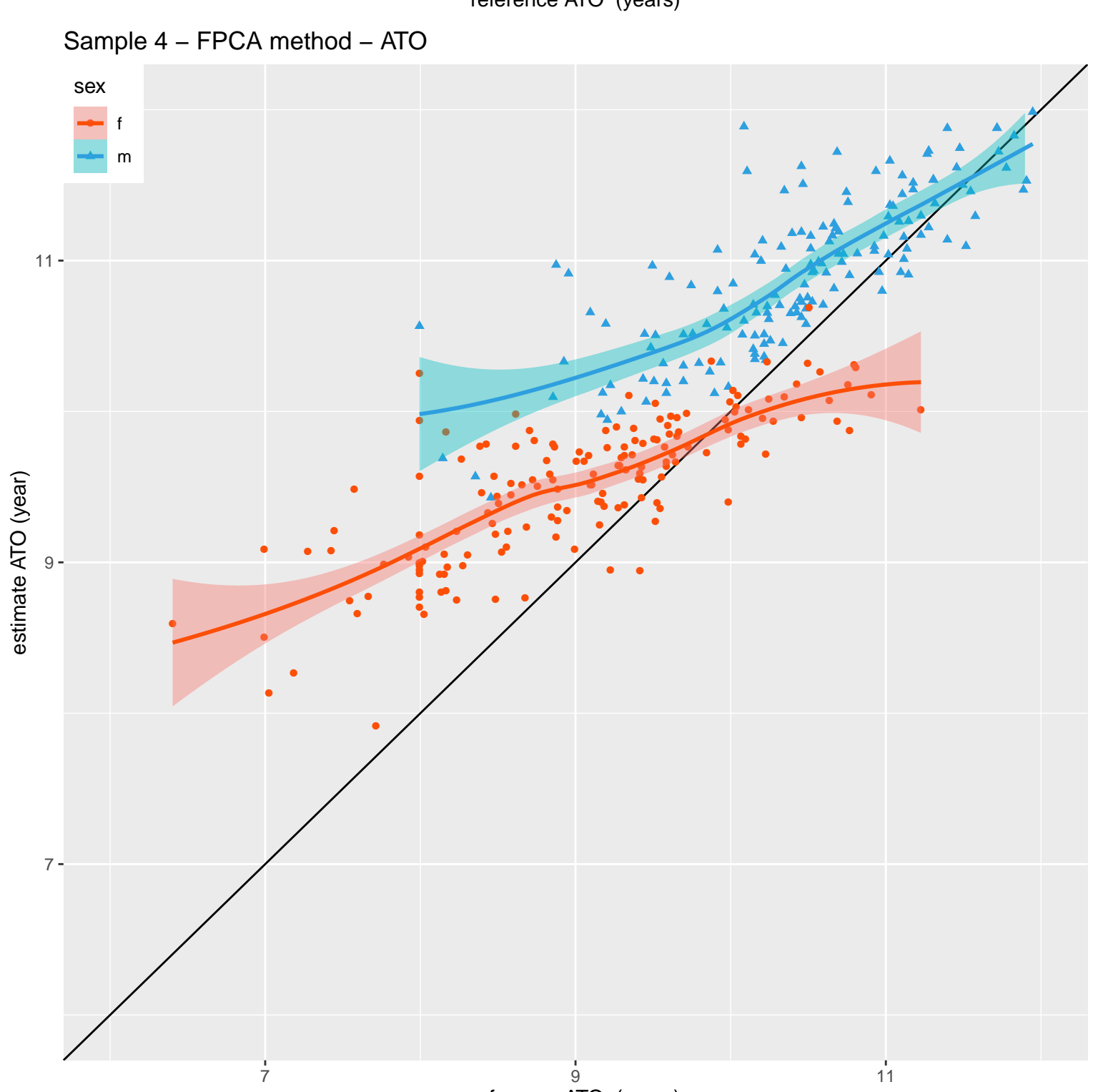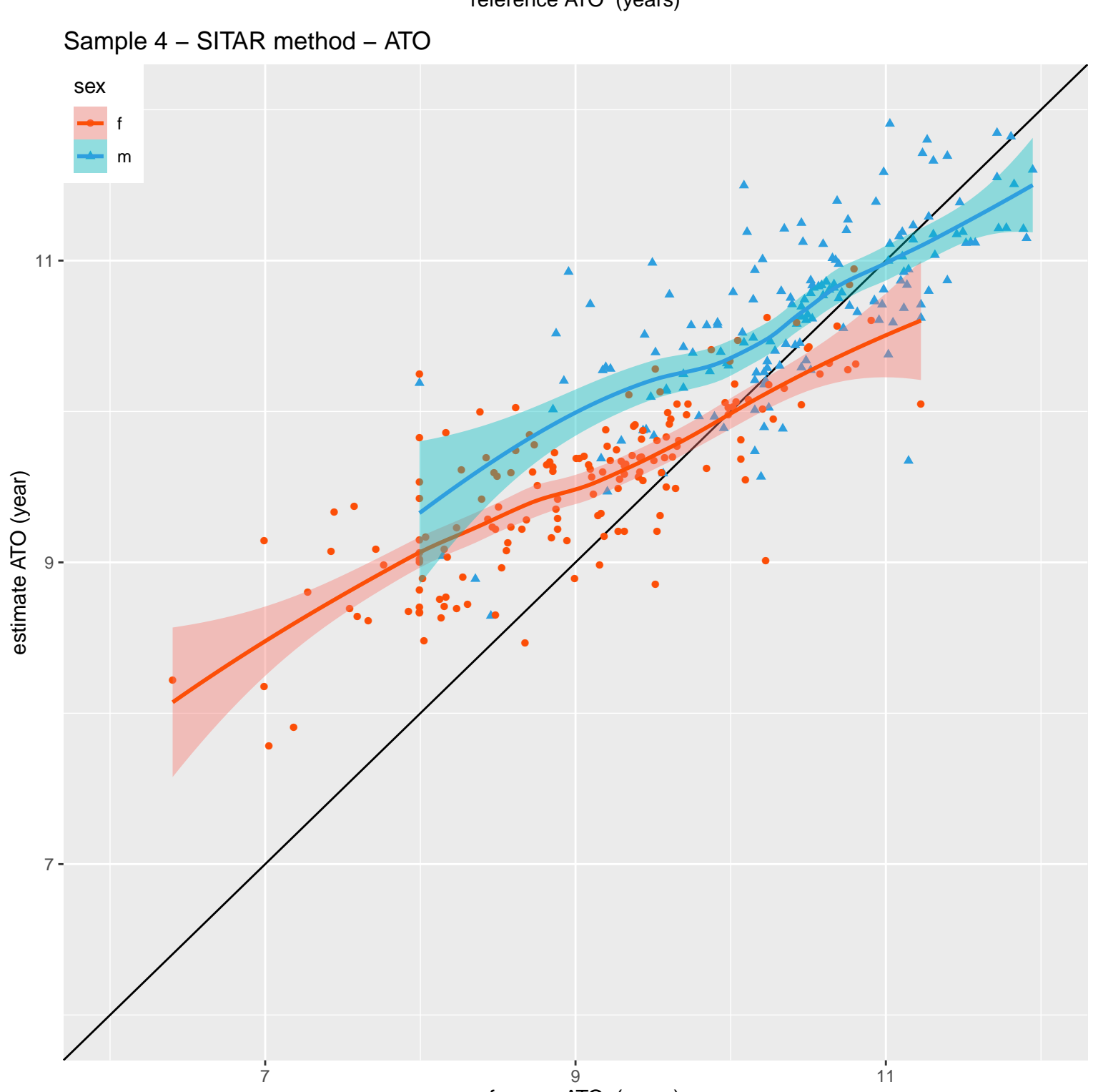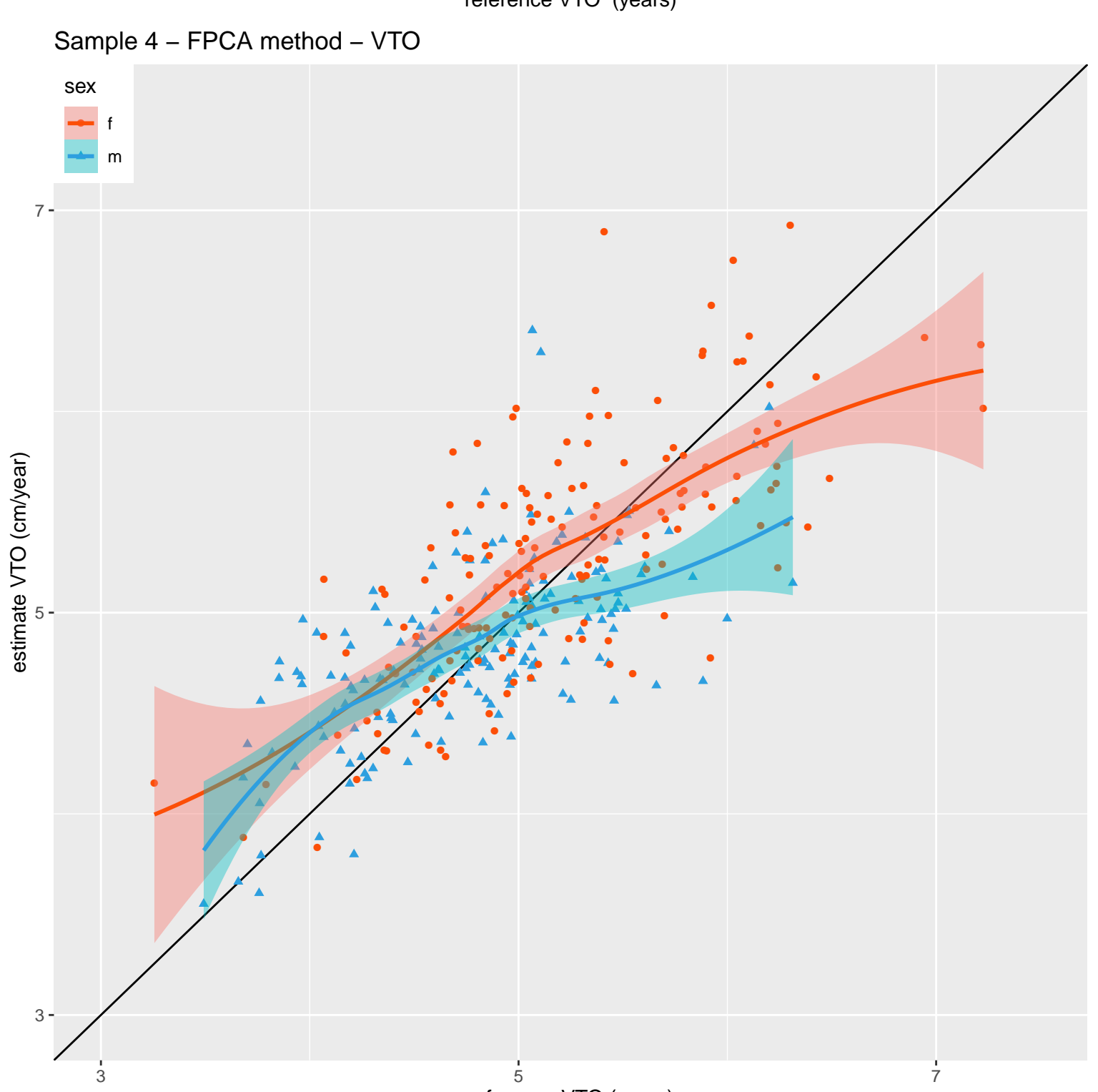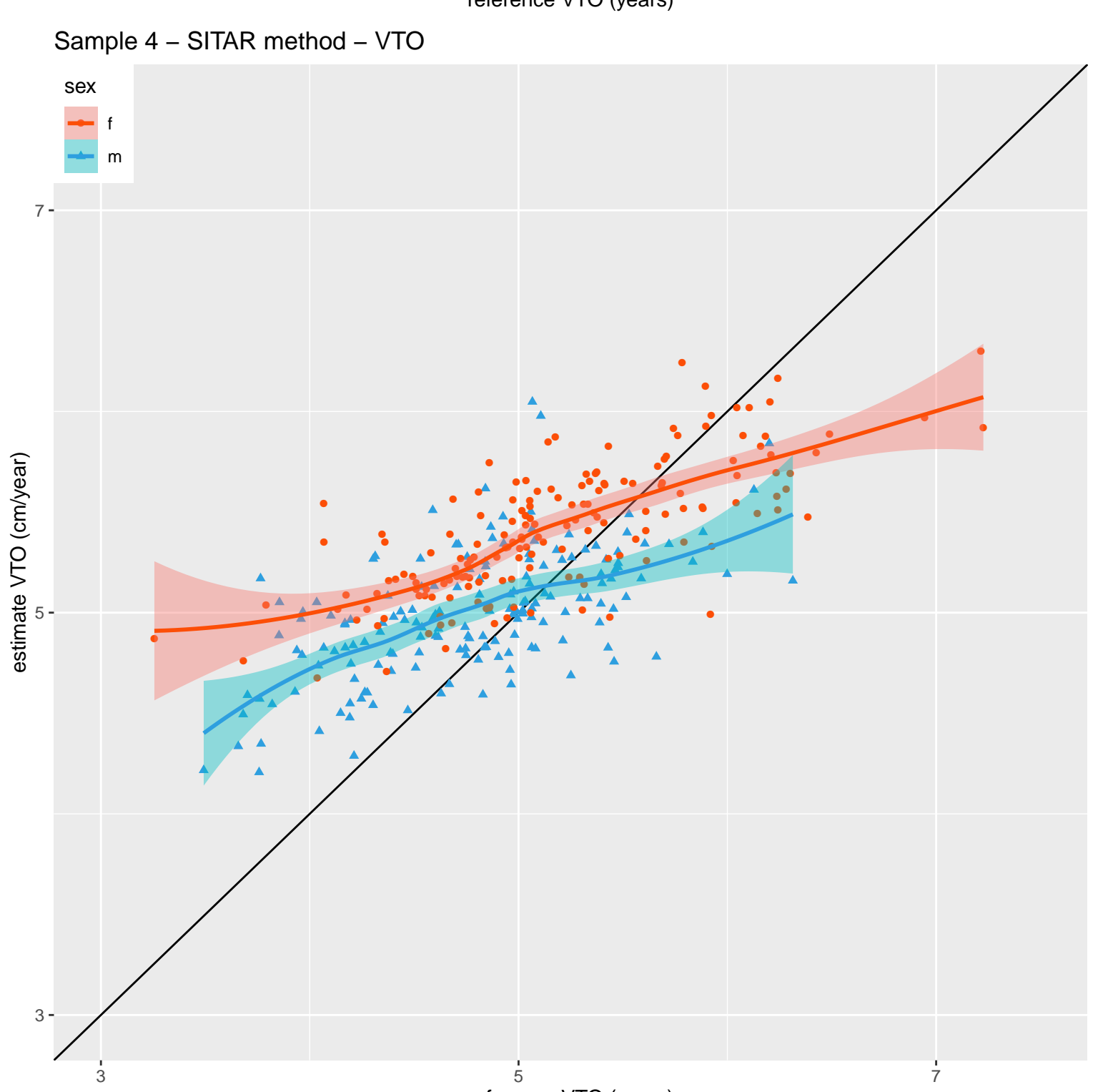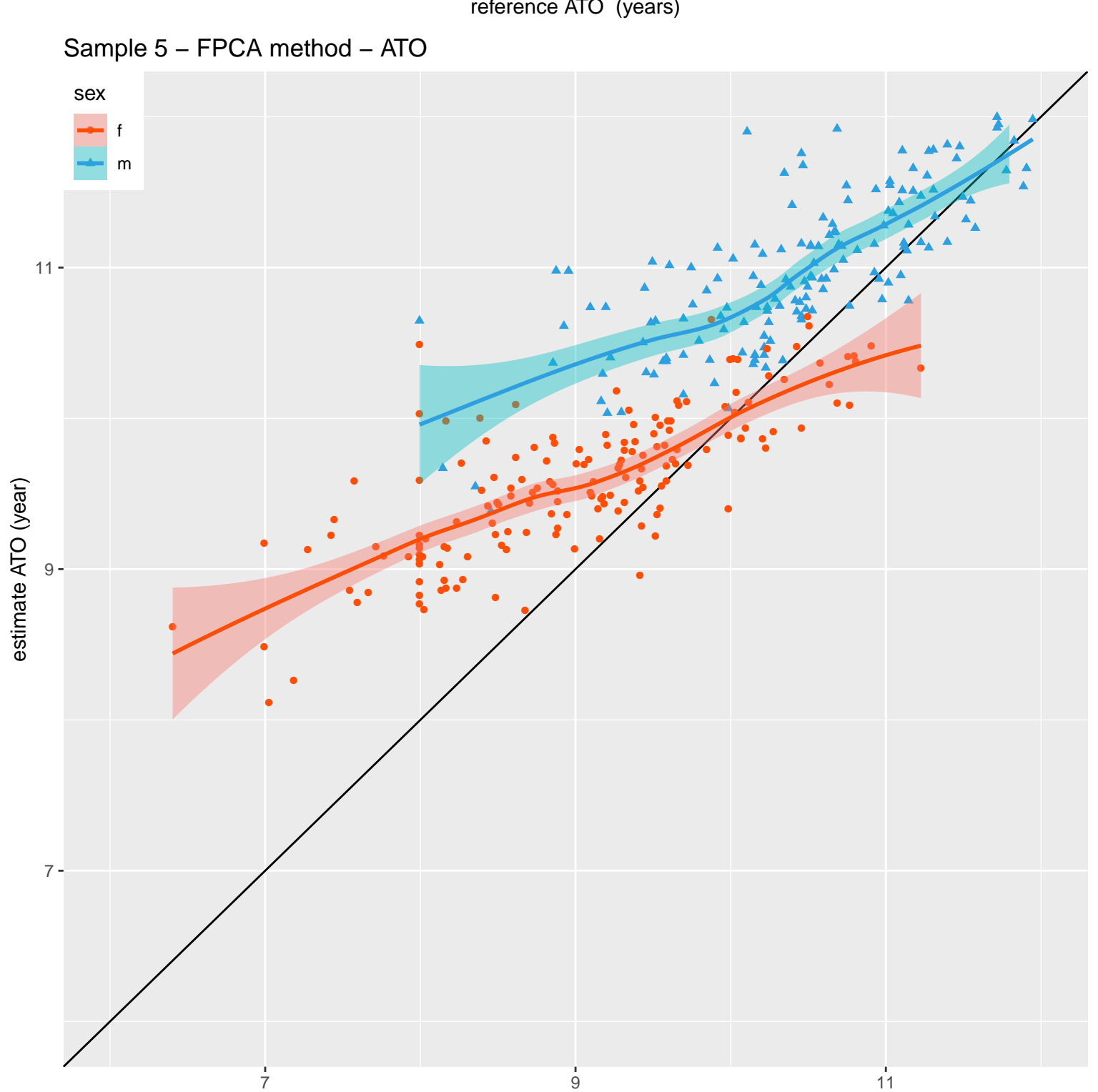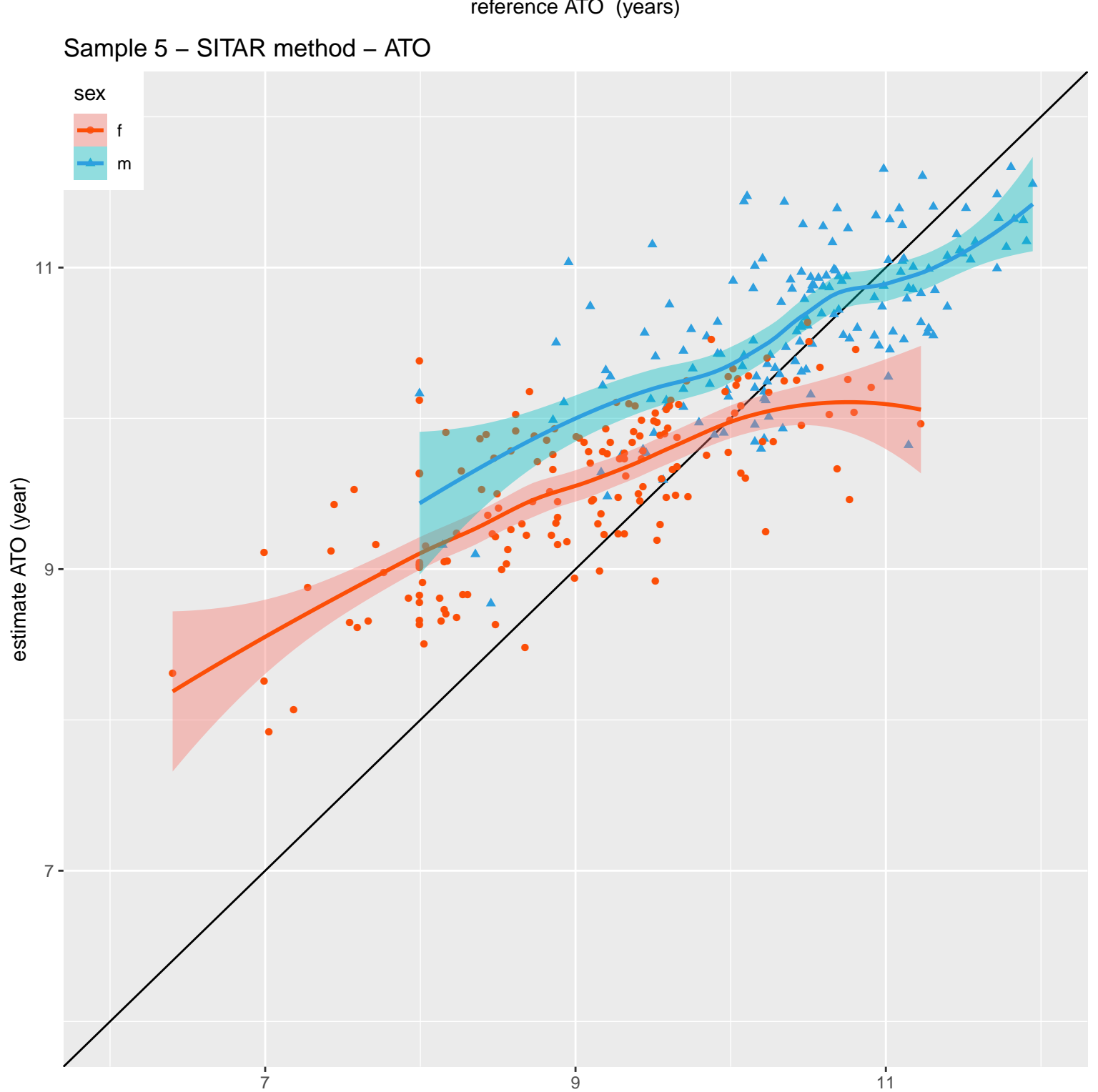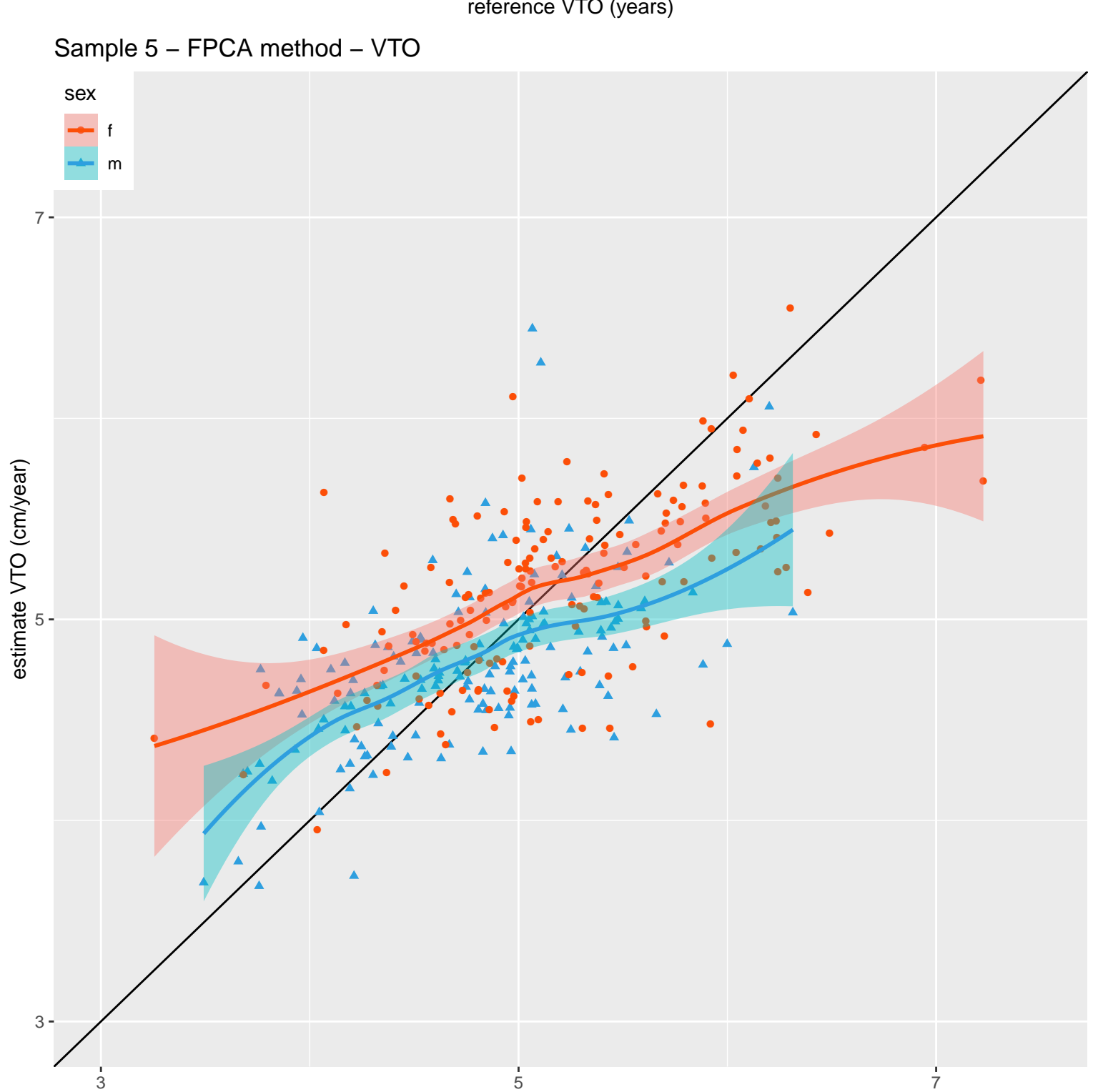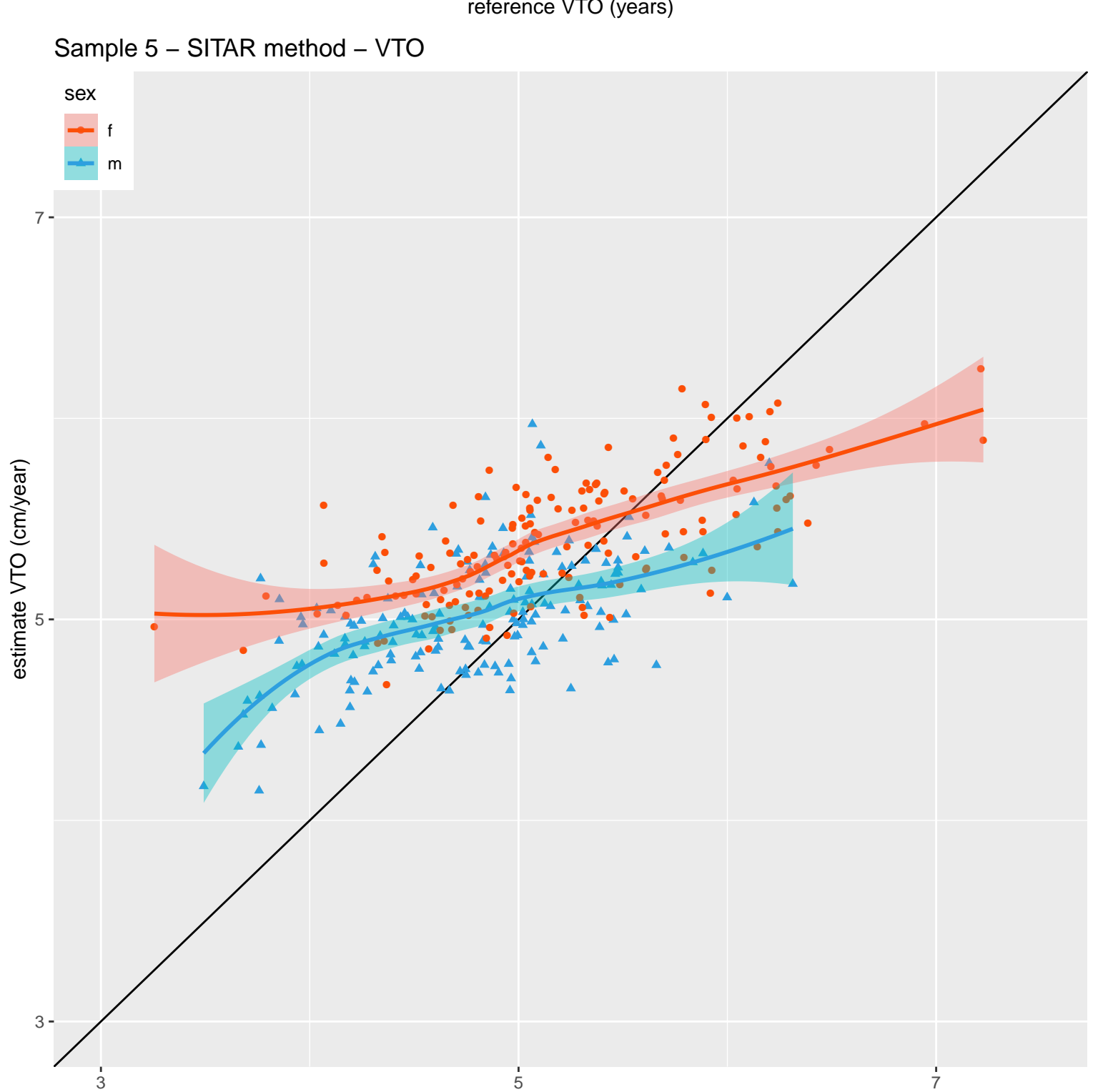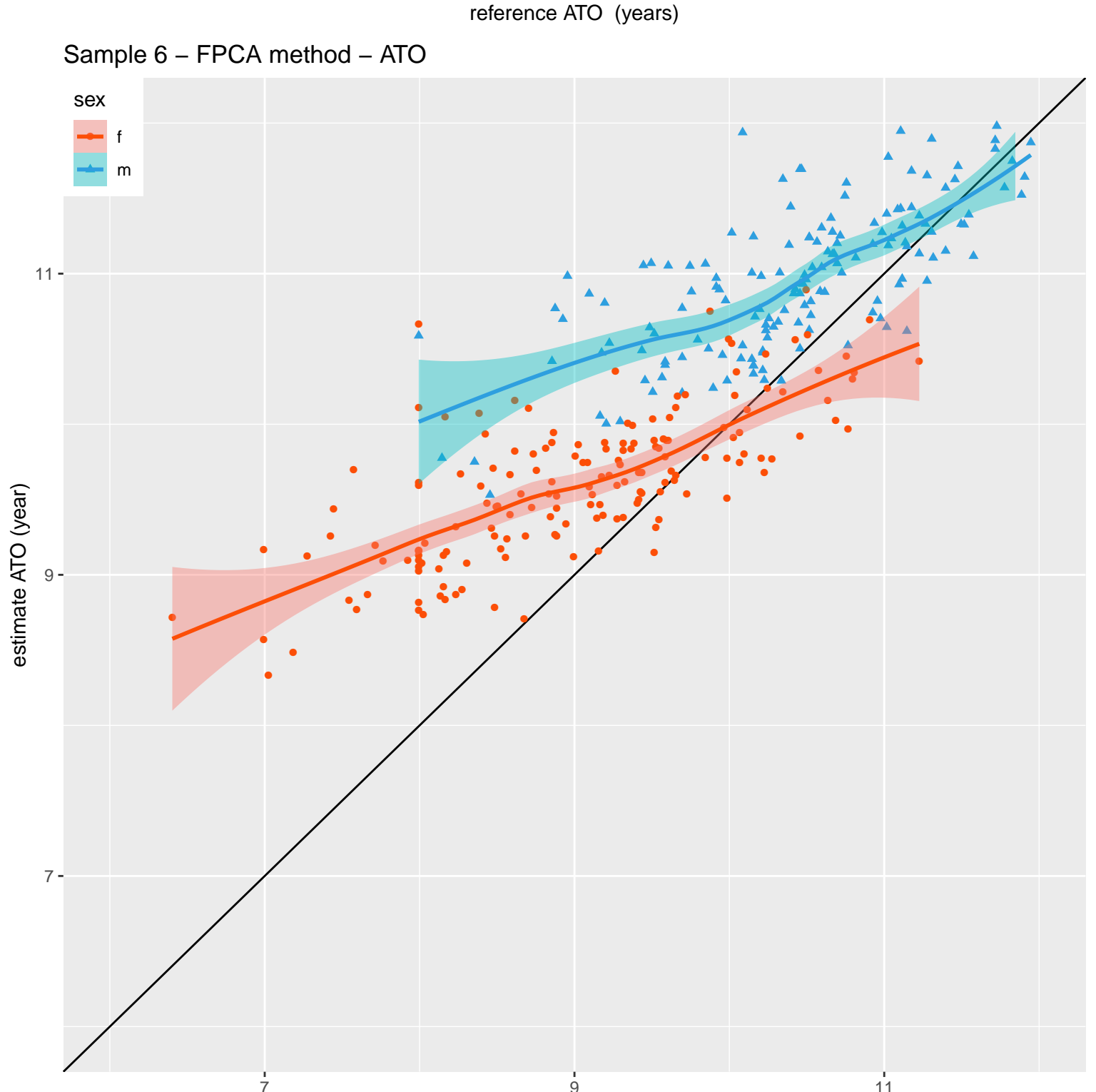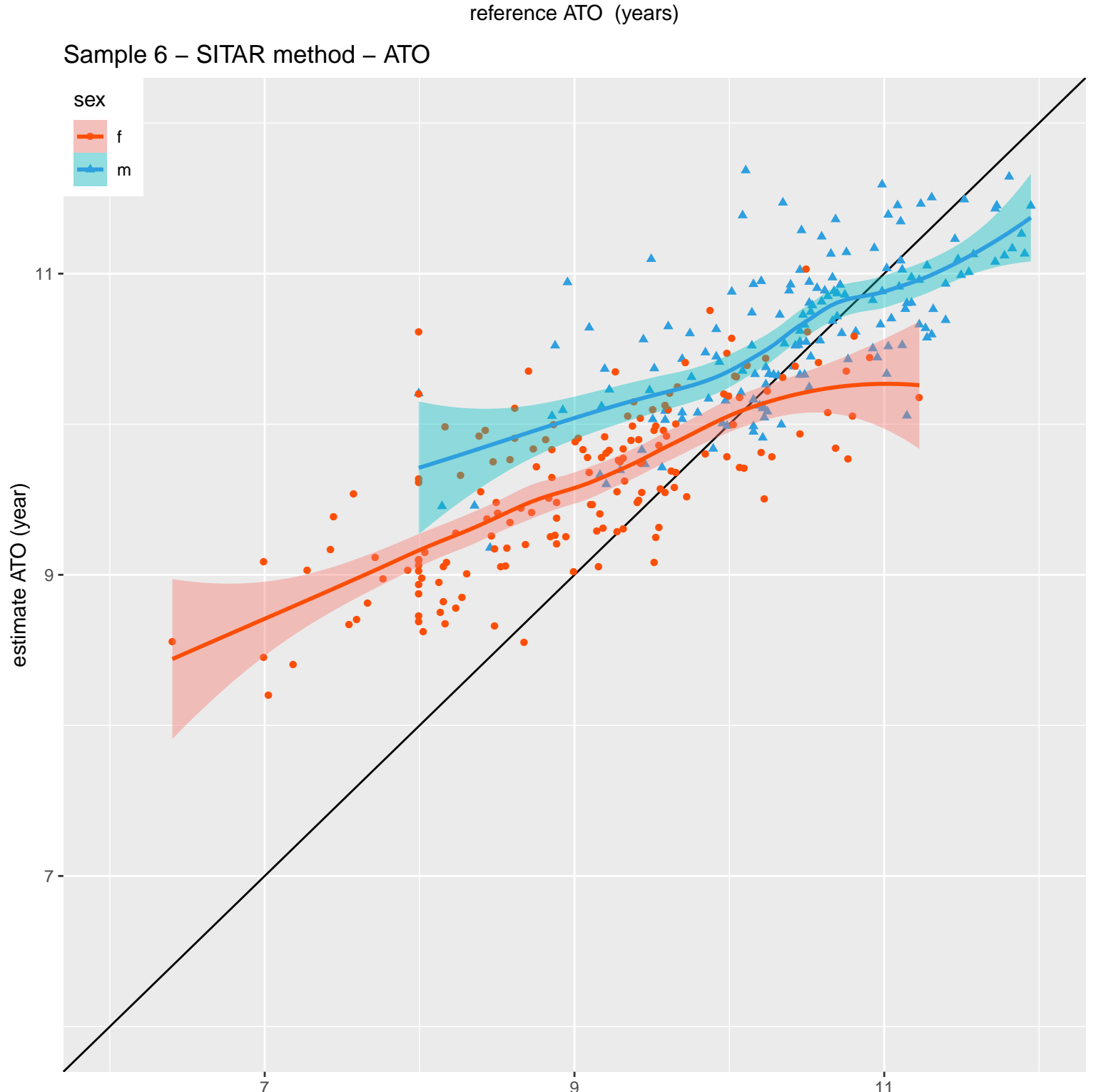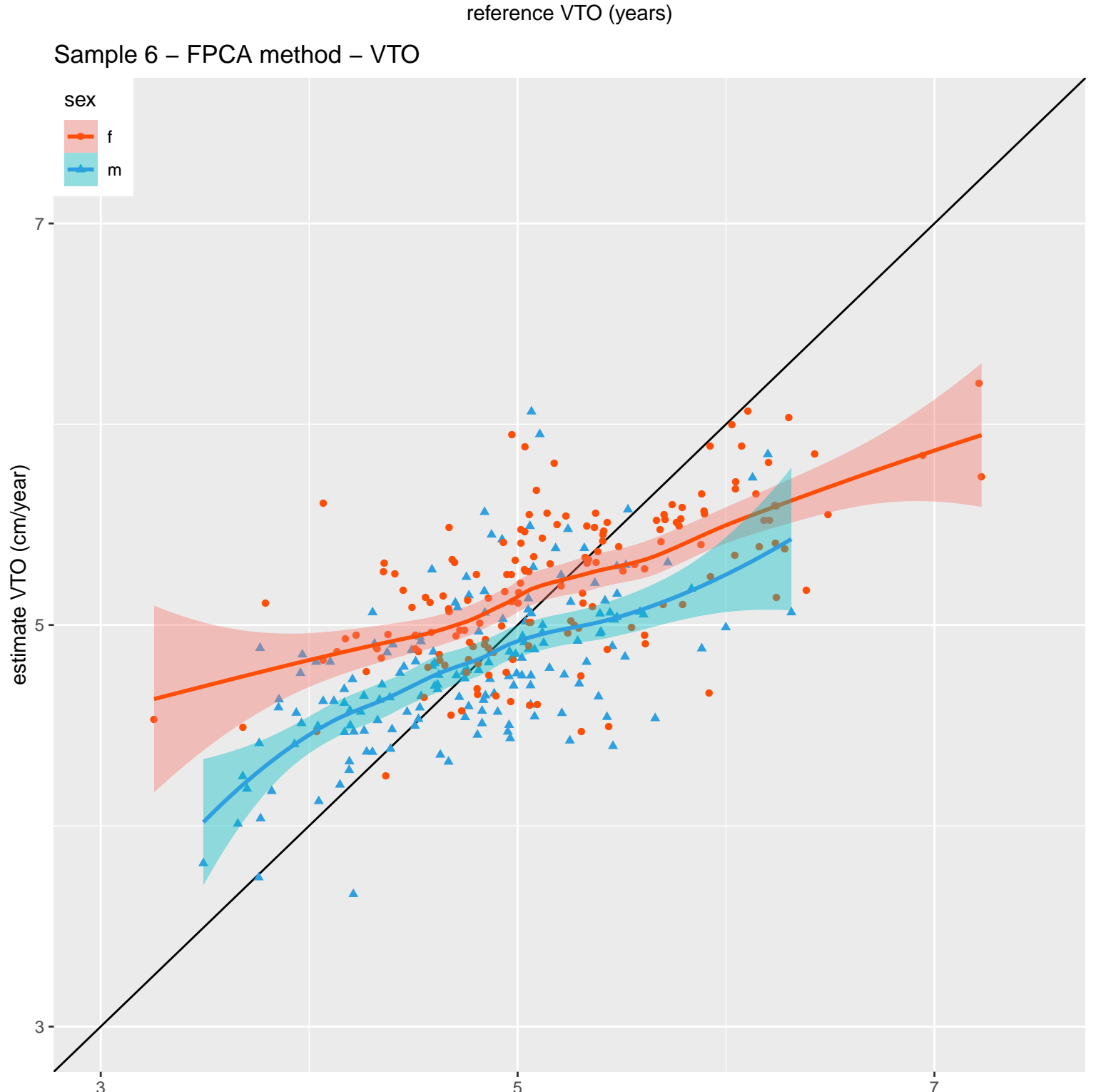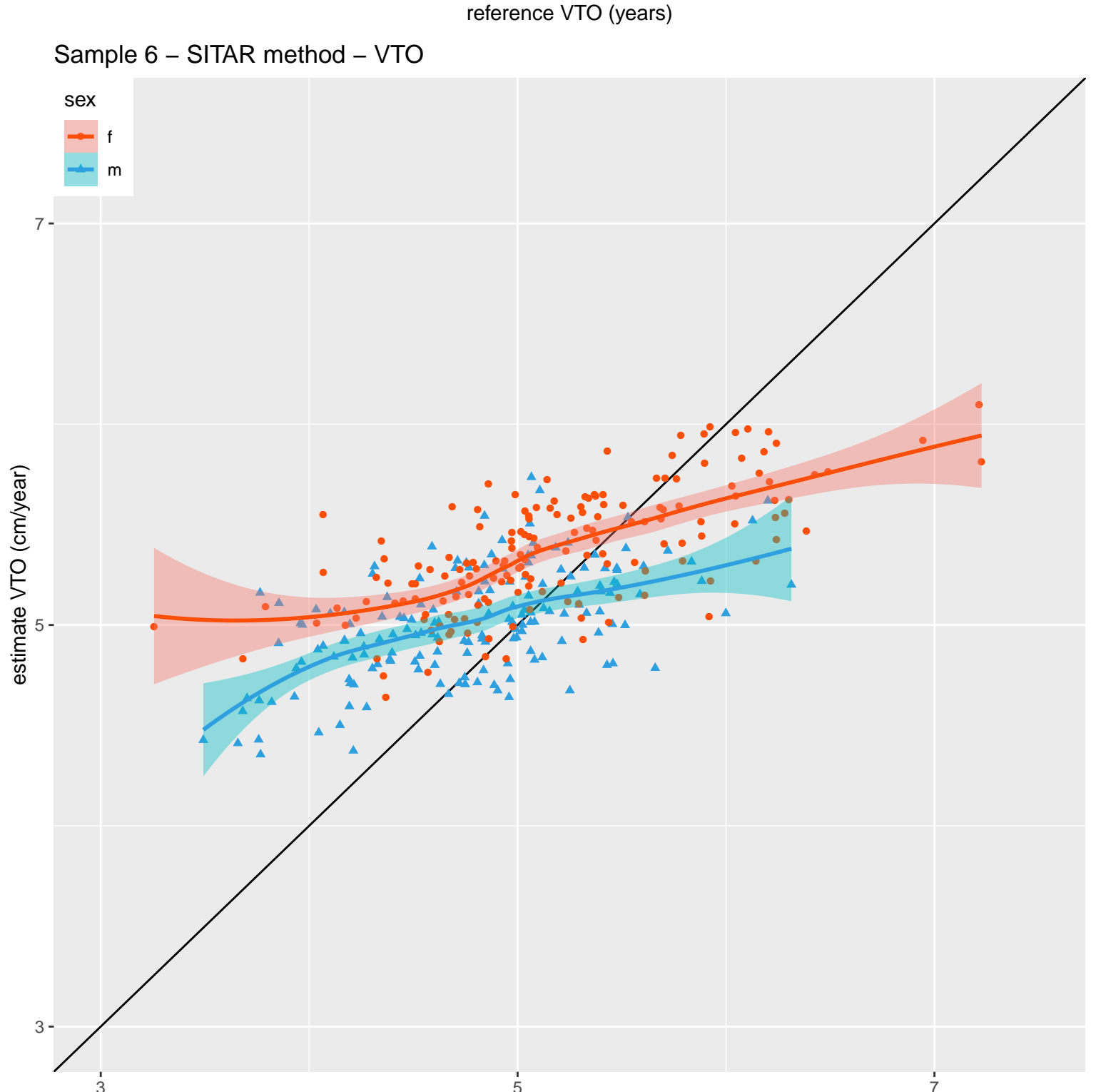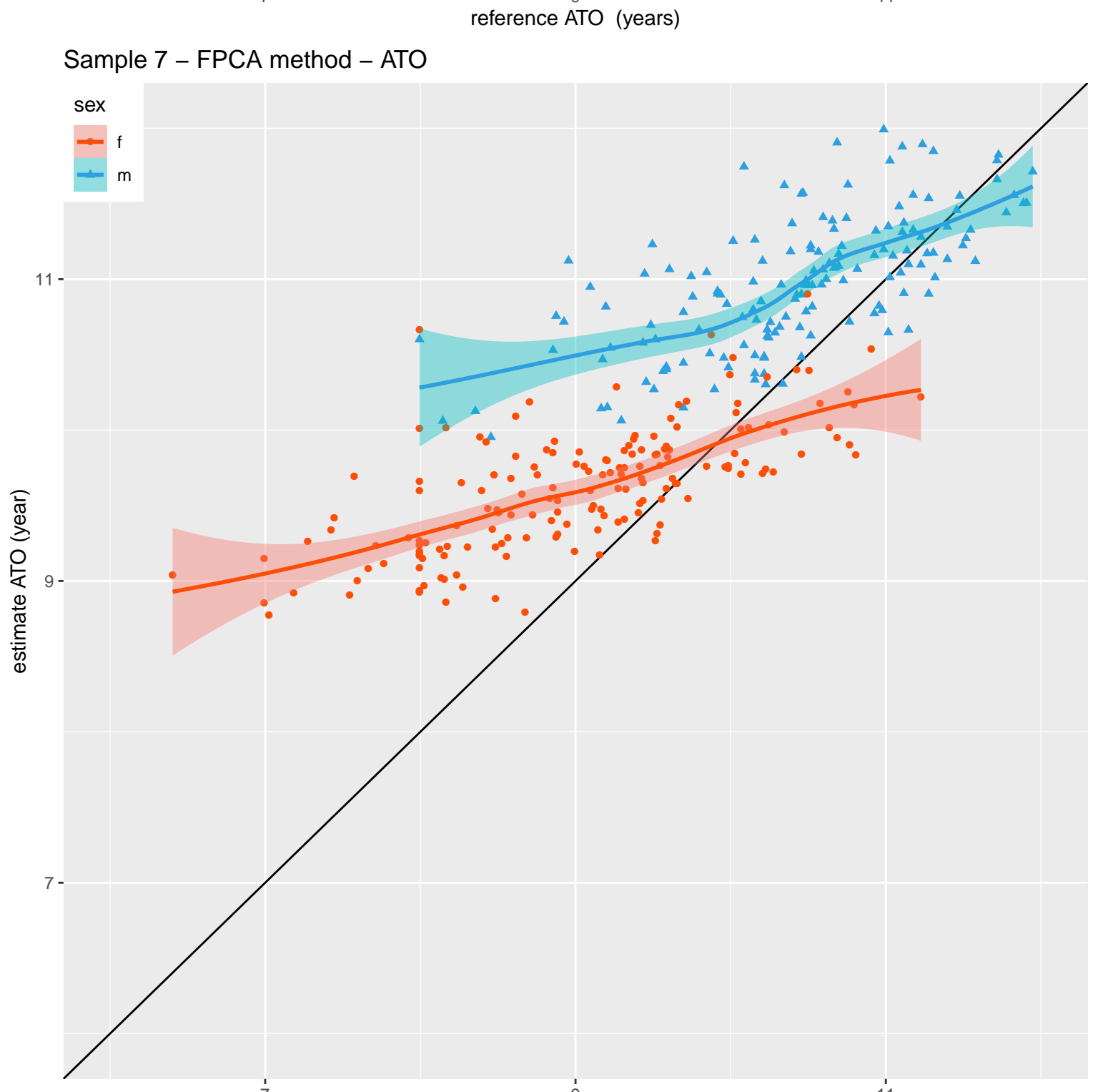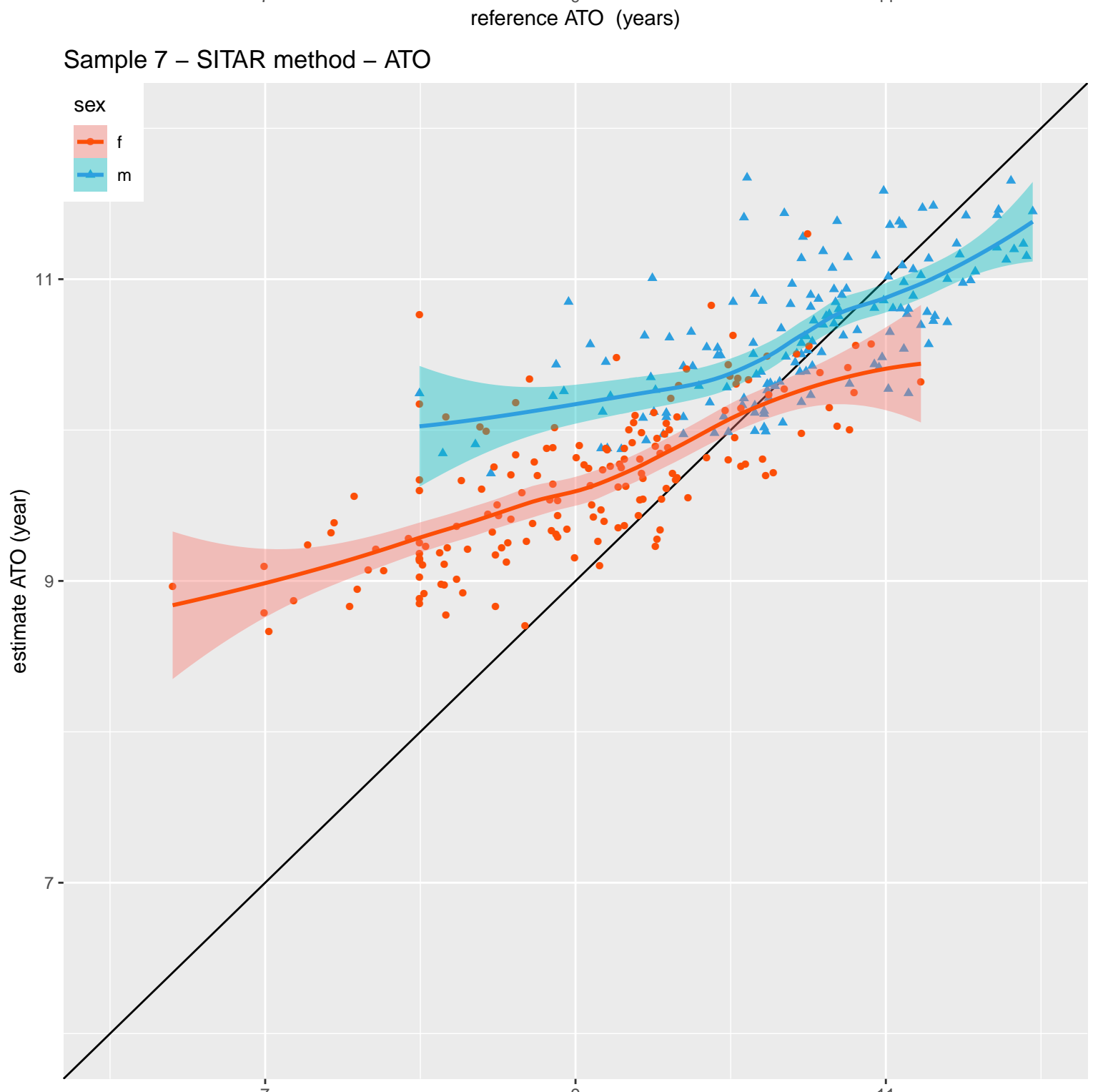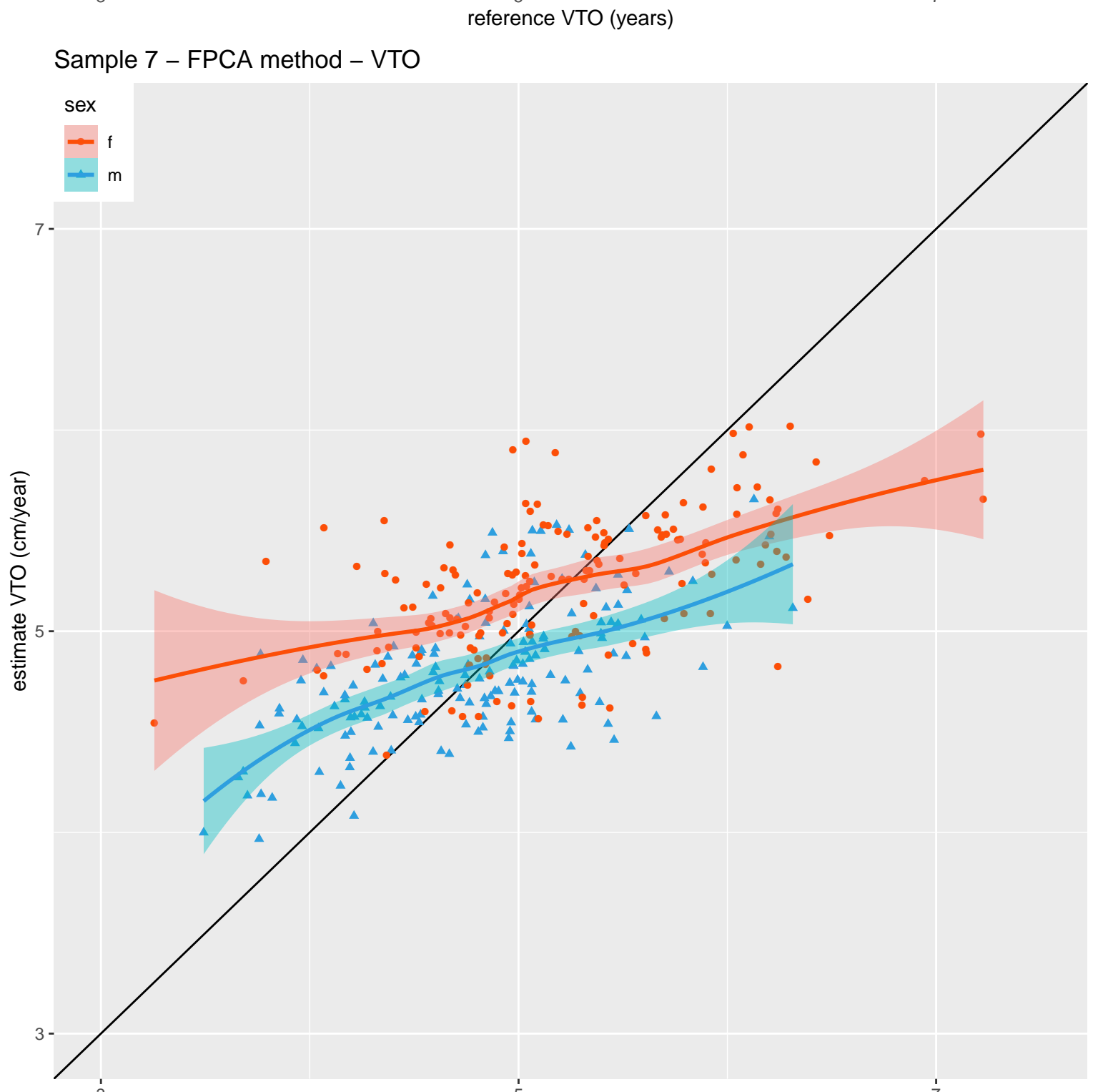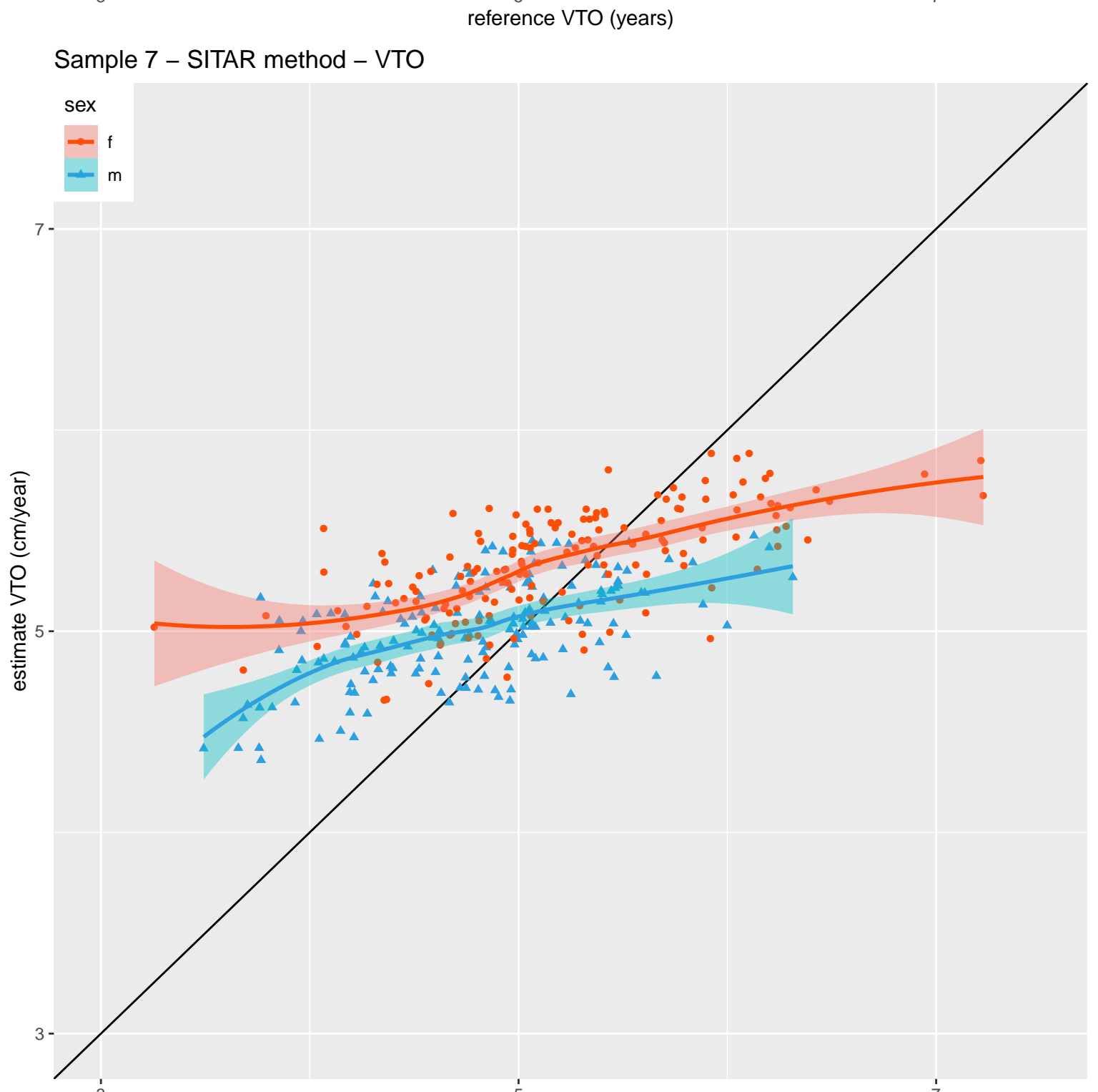

Supplement: Supplementary file 1 [file children-08-00934-s001.zip › Suplementary_materials/Figure_S09_ATO_Estimates_vs_reference.pdf]

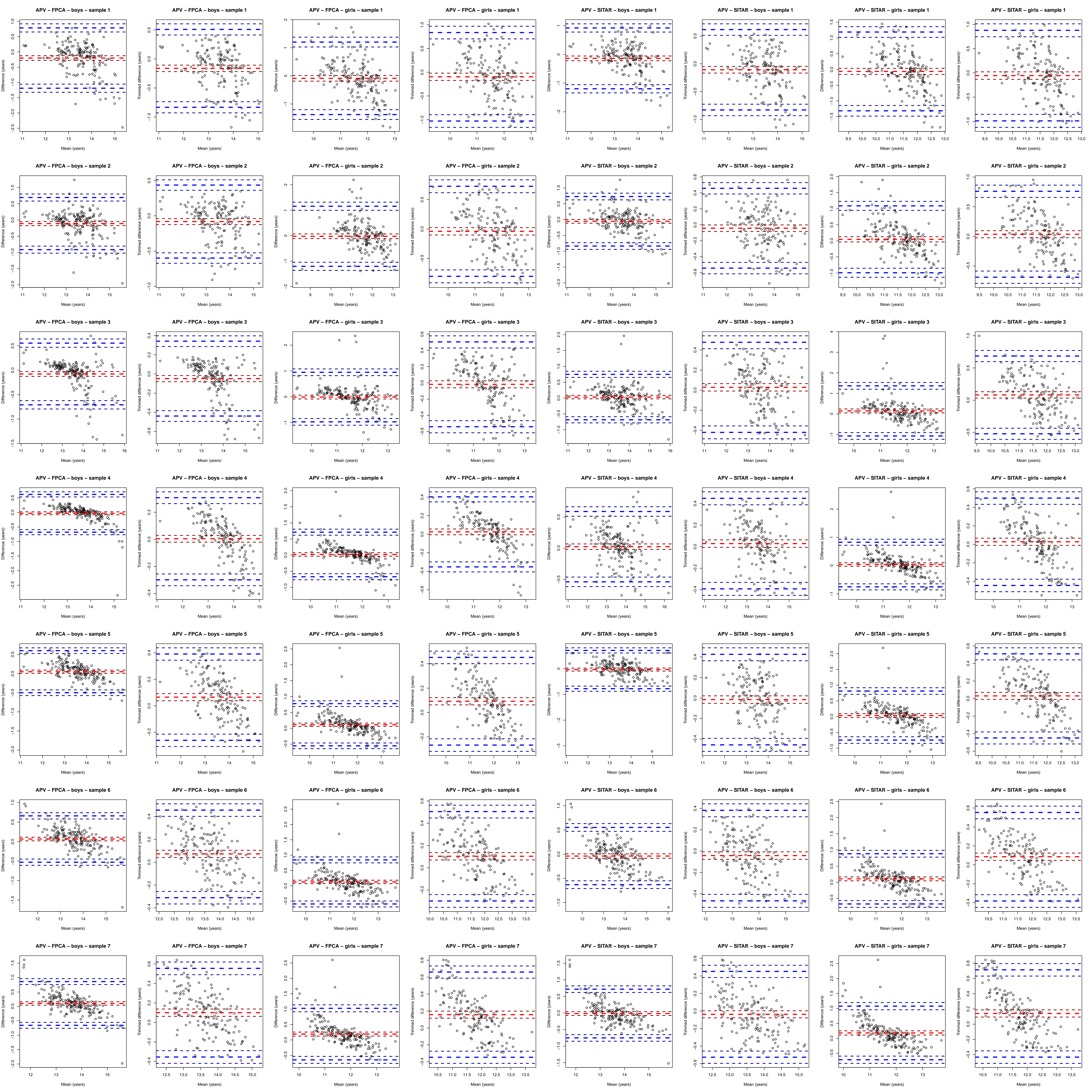

Supplement: Supplementary file 1 [file children-08-00934-s001.zip › Suplementary_materials/Figure_S10_Bland-Altman_APV.pdf]

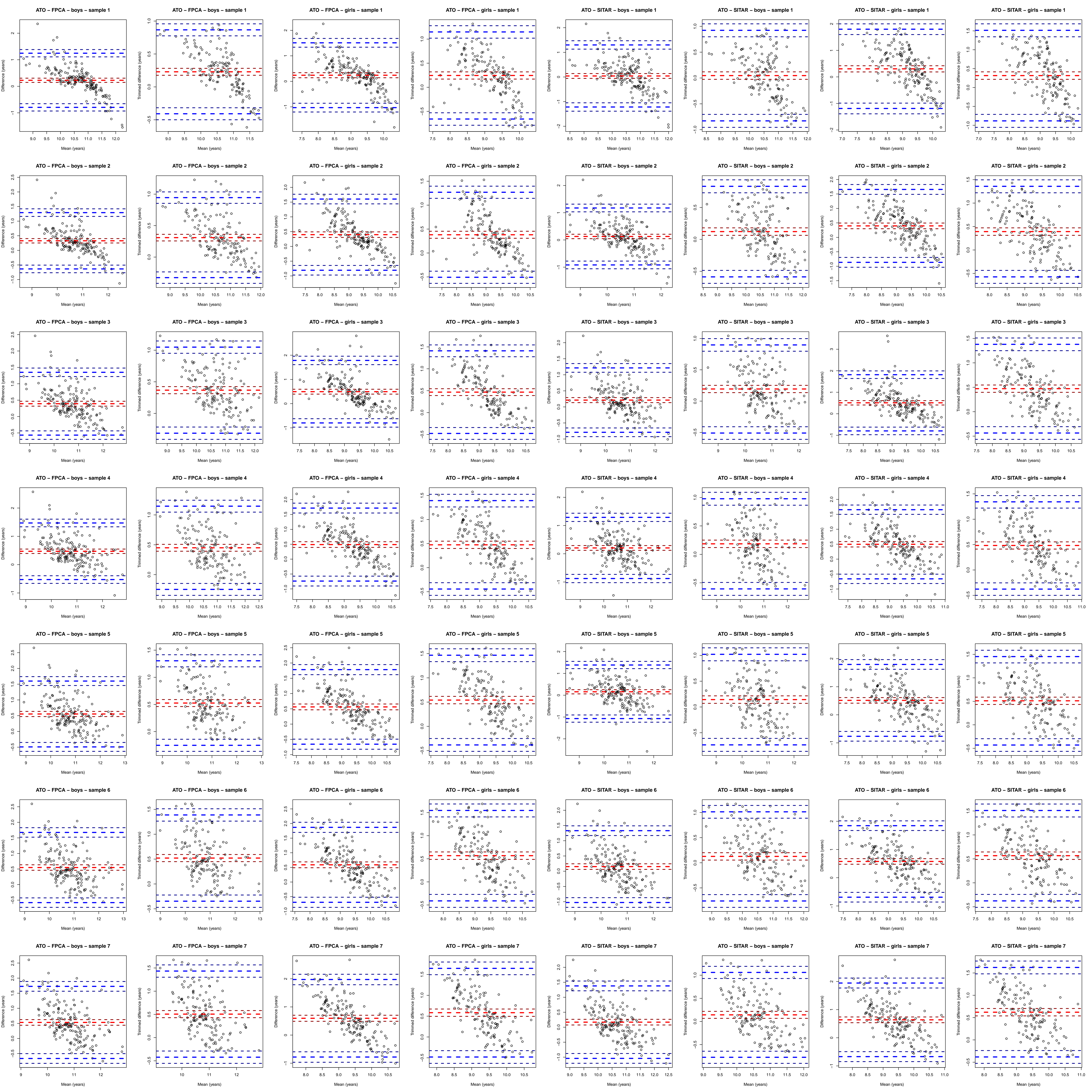

Supplement: Supplementary file 1 [file children-08-00934-s001.zip › Suplementary_materials/Figure_S11_Bland-Altman_ATO.pdf]
