# Supplementary material for: Estimating Growth in Height from Limited Longitudinal Growth Data Using Full-Curves Training Dataset: A Comparison of Two Procedures of Curve Optimization—Functional Principal Component Analysis and SITAR
Source: Children (Basel). 2021 Oct 18;8(10):934. doi: 10.3390/children8100934 (PMC8535004; doi:10.3390/children8100934)

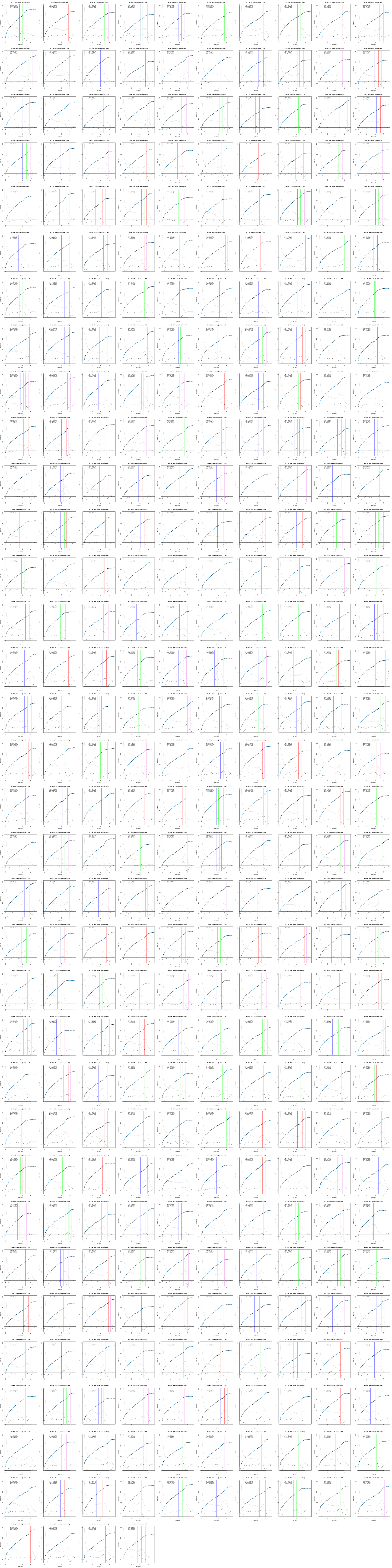

Supplement: Supplementary file 1 [file Figure_S03_BGS_reference_values_all.pdf › Suplementary_materials/Figure_S03_BGS_reference_values_all.pdf]
